# Truncated mini LRP1 transports cargo from luminal to basolateral side across the blood brain barrier

**DOI:** 10.1186/s12987-024-00573-1

**Published:** 2024-09-17

**Authors:** Laura Fritzen, Katharina Wienken, Lelia Wagner, Magdalena Kurtyka, Katharina Vogel, Jakob Körbelin, Sascha Weggen, Gert Fricker, Claus U. Pietrzik

**Affiliations:** 1 Molecular Neurodegeneration, Institute for Pathobiochemistry, University Medical Center of the Johannes Gutenberg-University of Mainz, Duesbergweg 6, 55099 Mainz, Germany; 2 Institute for Pharmacy and Molecular Biotechnology, University Heidelberg, Heidelberg, Germany; 3 Department of Neuropathology, Heinrich-Heine-University, Düsseldorf, Germany; 4 Department for Oncology and Hematology, University Medical Center Hamburg-Eppendorf, Hubertus Wald Cancer Center, Hamburg, Germany

**Keywords:** Low-density lipoprotein receptor-related protein 1 (LRP1), Blood-brain barrier (BBB), Drug delivery, Liposomes, γ-secretase modulator (GSM), Nanocarrier

## Abstract

**Background:**

The most crucial area to focus on when thinking of novel pathways for drug delivery into the CNS is the blood brain barrier (BBB). A number of nanoparticulate formulations have been shown in earlier research to target receptors at the BBB and transport therapeutics into the CNS. However, no mechanism for CNS entrance and movement throughout the CNS parenchyma has been proposed yet. Here, the truncated mini low-density lipoprotein receptor-related protein 1 mLRP1_DIV* was presented as blood to brain transport carrier, exemplified by antibodies and immunoliposomes using a systematic approach to screen the receptor and its ligands’ route across endothelial cells *in vitro.*

**Methods:**

The use of mLRP1_DIV* as liposomal carrier into the CNS was validated based on internalization and transport assays across an in vitro model of the BBB using hcMEC/D3 and bEnd.3 cells. Trafficking routes of mLRP1_DIV* and corresponding cargo across endothelial cells were analyzed using immunofluorescence. Modulation of γ-secretase activity by immunoliposomes loaded with the γ-secretase modulator BB25 was investigated in co-cultures of bEnd.3 mLRP1_DIV* cells and CHO cells overexpressing human amyloid precursor protein (APP) and presenilin 1 (PSEN1).

**Results:**

We showed that while expressed in vitro, mLRP1_DIV* transports both, antibodies and functionalized immunoliposomes from luminal to basolateral side across an in vitro model of the BBB, followed by their mLRP1_DIV* dependent release of the cargo. Importantly, functionalized liposomes loaded with the γ-secretase modulator BB25 were demonstrated to effectively reduce toxic Aß_42_ peptide levels after mLRP1_DIV* mediated transport across a co-cultured endothelial monolayer.

**Conclusion:**

Together, the data strongly suggest mLRP1_DIV* as a promising tool for drug delivery into the CNS, as it allows a straight transport of cargo from luminal to abluminal side across an endothelial monolayer and it’s release into brain parenchyma in vitro, where it exhibits its intended therapeutic effect.

**Supplementary Information:**

The online version contains supplementary material available at 10.1186/s12987-024-00573-1.

## Background

In the healthy brain, the blood brain barrier (BBB) protects the brain from exposure of exogenous and endogenous particles circulating in the blood that could be harmful to the brain [1]. The protection of the brain can be maintained by a functional interplay of different cell types including endothelial cells of the capillary wall, astrocytes and pericytes [2]. To fulfill the protection of the brain, the brain capillary endothelial cells (BCEC) distinguish from the rest of the peripheral endothelial cells by several characteristics [3]. Importantly, BCEC show an absence of fenestrae, an extremely low pinocytotic activity as well as the presence of tight junctions (TJ) and other junctional complexes of high electrical resistance. Together those characteristics provide an effective barrier against molecules and limit paracellular movement by sealing the space between two endothelial cells [4–7]. However, this organized machinery not only provides an effective barrier against molecules, but also regulates the entry as well as the outflow of molecules across the BBB by different transport mechanisms including paracellular diffusion, transport via solute carrier transporters (SLCs), transcellular diffusion, and receptor-mediated transcytosis (RMT) [8, 9]. In particular, RMT represents one of the most important transport mechanisms, as it allows a specific and controlled transport of molecules by different receptor and transporter expression on the luminal and abluminal side of endothelial cells [10, 11]. In this context, a disruption of BBB’s functionality contributes to pathology in a wide range of CNS disorders including multiple sclerosis, stroke, epilepsy, and Alzheimer’s disease (AD). AD is a chronic, neurodegenerative disease leading to dementia with impairment of cognitive and behavioral functions and failure to maintain activities of daily living [12]. AD neuropathology is characterized by intracellular neurofibrillary tangles consisting of the Tau protein and by extracellular amyloid ß (Aß) plaques, which consist of Aß peptides derived from the amyloid precursor protein (APP). The beta-secretase, e.g. BACE1, and the γ-secretase complex sequentially process APP, resulting in the generation of Aß peptides, which occur in a variety of isoforms ranging in length from 36 to 46 amino acids, with Aβ_42_ being the most toxic variant [13, 14]. The pathophysiology of AD is primarily caused by aberrant accumulation of Aβ_42_ peptides in the brain, likely due to a decreased clearance of cerebral Aß_42_ in AD patients compared to healthy individuals. Thus, several strategies including decreasing Aβ production, preventing its aggregation, or improving Aβ clearance from the brain are being considered to slow disease progression. However, curing of the most CNS disorders is mostly limited, as most developed drugs are not able to traverse the BBB and enter the brain. A key challenge in drug development is therefore to design not only therapeutics to treat the disease, but rather treatment strategies allowing a penetration of CNS-active drugs across the BBB. Targeting endogenous receptors, e.g. LRP1 or TfR-1 enabling RMT could be a promising opportunity for enhancing drug delivery across the BBB. RMT is based on endogenous receptors expressed on the luminal side of the BBB. These receptors use vesicular trafficking of ligand-receptor complexes to deliver macromolecule nutrients, such as iron-bound transferrin, insulin, and leptin, into the brain side. The RMT transport pathway necessitates binding to the receptor’s extracellular domain, followed by endocytosis and transcytosis to the capillary endothelium’s abluminal side into the interstitial space. In this context, the majority of research on protein and antibody delivery has focused on the transferrin receptor (TfR) or LRP1, due to their high expression level in BECs [15–18]. However, the use of endogenous receptors, like LRP1 or TfR, that are ubiquitously expressed in different cell types and tissues might induce severe side effects, as the drug delivery is not strictly limited to the CNS. On the one hand, targeting therapeutics could compete with natural ligands, which may perturb the normal biological functions of the receptor. In this context, clinical trials of trontinemab, a new version of the anti-amyloid monoclonal antibody gantenerumab, engineered to more easily cross the BBB by binding to TfR-1, reported some side effects in form anemia. Thereby, iron deficiency is the most frequent cause of anemia [19, 20]. Since trontinemab competes with the binding of natural ligands of TfR-1, e.g. iron-bound transferrin, the normal biological function of TfR, the transport of free iron from the serum into cells, is disrupted accordingly. Additionally, targeting systemically expressed endogenous receptors may induce adverse side effects due to the accumulation of therapeutics in other organs or tissues, as the delivery is not strictly limited to the CNS. Furthermore, in neurological and/or neurodegenerative disorders linked to vascular dysfunction, RMT deficit is anticipated to arise frequently, if not always, in the affected brain regions [21]. This could include deficiencies in TfR and/or LRP1 expression itself and/or anomalies in the endocytic and exocytotic molecular machinery leading to variances in brain shuttle effectiveness in the population afflicted by brain disorders [22].

For this reason, the generation of artificial receptors based on endogenous ones, but lacking their physiological ability to bind natural ligands, could serve as alternative targeting system, due to their similar sorting and transport behavior, but still unique expression to certain tissues. In this context, the use of adeno-associated virus (AAV) based gene therapy offers not only a specific infection of only desired tissues and cells, e.g. endothelial cells of the BBB, but also show a high transduction rate of target organs, low immunogenicity in *vivo*, and sustainable expression for month or years [23]. Besides targeting receptors at the BBB, the use of liposomes or nanoparticles (NPs) encapsulating different types of drugs seems to be a very sophisticated approach and has been successfully used for drug delivery across the BBB in vitro and in vivo [24–28]. Thereby, the usage of nanoparticles offers many benefits compared to a systemic drug delivery approach including high drug-loading capacity of the nanoparticles as well as the protection of encapsulated substances against enzymatic or chemical degradation while circulating in the blood, which might extend their half-life. Moreover, a modification of nanoparticles’ surfaces also allows them to be actively directed toward a specific tissue including the brain parenchyma following the route of RMT [27, 29]. To date, different targeting strategies can be employed, however the most common form of active targeting is the coupling of antibodies or antibody fragments to the surface of liposomes due to their high specificity. The so-called immunoliposomes are considered as the most promising classes for medical applications nowadays [30, 31]. In this context, the nanoparticulate nonsteroidal anti-inflammatory drug (NSAID) flurbiprofen, which is commonly used for treatment of pain, fever or other inflammatory conditions, was shown to reduce Aß_42_ levels in an in vitro BBB model [32–35]. Besides their intended use in the treatment of pain, NSAIDs, such as indomethacin, flurbiprofen or ibuprofen were considered for the treatment of AD due to their γ-secretase modulating (GSM) activity. GSMs are small molecules that reduce the levels of the amyloidogenic Aß_42_ peptides and promote the generation of shorter, less aggregation-prone Aß peptides like Aß_38_, and were shown to function as allosteric activators of γ-secretase activity [36, 37]. However, NSAIDs such as ibuprofen or flurbiprofen have pharmacological disadvantages, including low GSM activity and brain permeability [38]. More recently, GSMs with favorable pharmacological characteristics and nanomolar potency have been described. For example, after treatment of CHO cells with stable co-expression of human APP and presenilin-1 (PSEN1), the acidic GSM BB25 displayed typical GSM characteristics and decreased Aß_42_ levels with an IC_50_ value of 87 nM and a concomitant increase in Aß_38_ levels [39]. In general, embedding already approved drugs or compounds like GSMs into liposomal formulations that target receptors at the BBB seems to be a promising way enhancing drug delivery into the CNS. For this reason, we designed a proof-of concept study investigating the potential of an artificial truncated LRP1 variant for transport purposes across the BBB. In this proof-of-concept study, the use of the artificial mini LRP1 receptor mLRP1_DIV* as functional antibody and immunoliposome carrier into the CNS was validated. Besides internalization and transport of antibodies and functionalized immunoliposomes across an in vitro model of the BBB, the exact trafficking route of mLRP1_DIV* and corresponding cargo and their abluminal release into the brain parenchyma was demonstrated. Since the γ-secretase modulator BB25 was already shown to modulate Aß generation in vitro, the chemical compound was encapsulated into immunoliposomes. Finally, to confirm the artificial mRLP1_DIV* construct as cargo carrier across the BBB, the modulation of γ-secretase activity by transported liposomal BB25 across endothelial mLRP1_DIV* cells was confirmed in co-culture with CHO cells overexpressing APP and PSEN1. Furthermore, mLRP1_DIV*’s functional role as transport shuttle was explored in the in vivo situation with regard to its relevance for neurodegenerative diseases. To provide an in vivo evidence that LPR1-based receptors can be used as therapeutic strategy, an adeno-associated virus (AAV) that specifically infects only endothelial cells of the BBB was used. Due to the development of this highly specific AAV, only the BBB associated endothelium can be efficiently infected after intravenous injection [40]. mLRP1_DIV*’s expression in the endothelium after intravenous injection was validated by isolation of brain capillaries and endothelial cells from mice, followed by immunofluorescence and lysis of corresponding tissue and western blot analysis.

## Methods

### Antibodies

A list of antibodies used can be found in the Supplementary Data file 1.

### mLRP1_DIV* design and structure

**Fig. 1:**
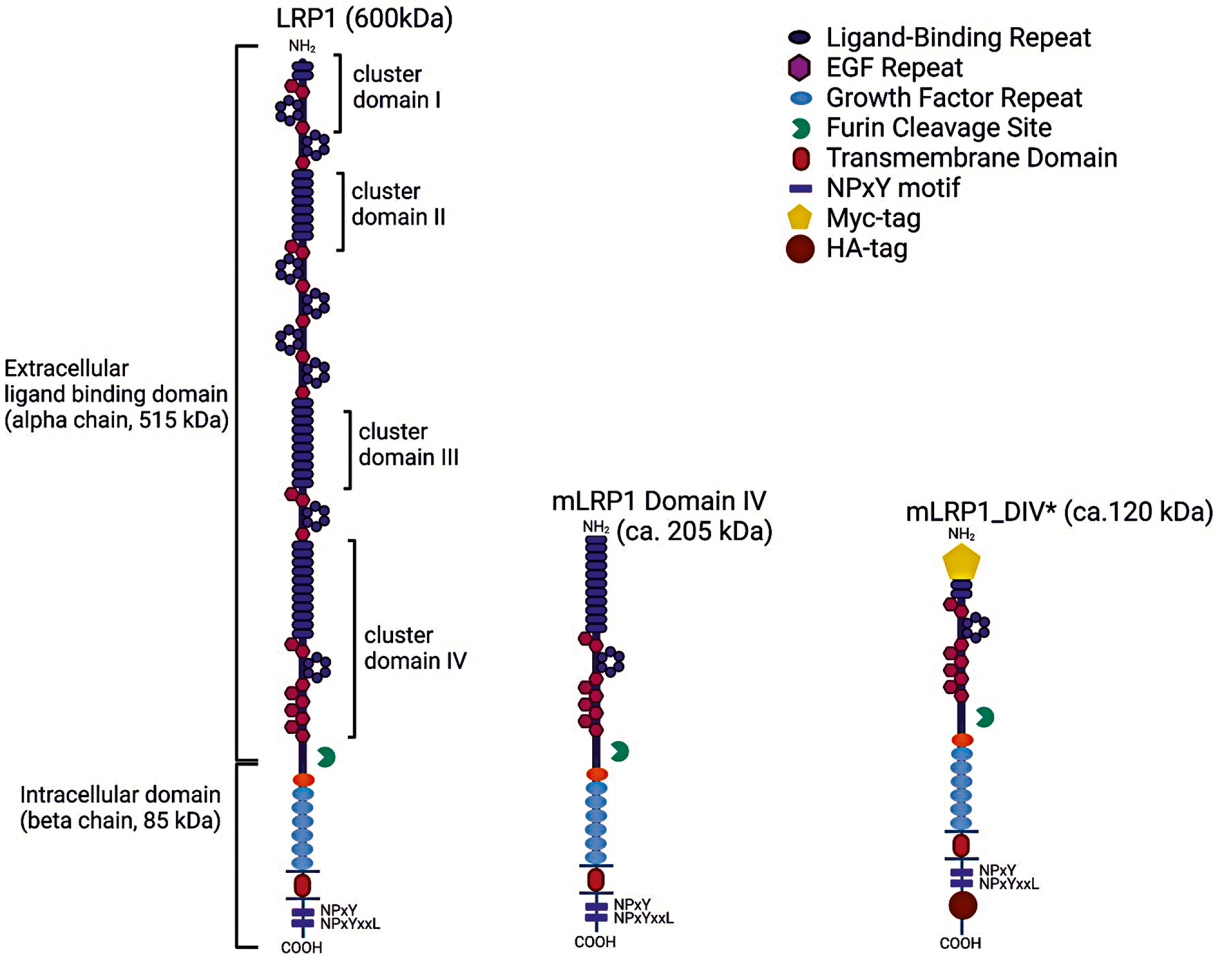
LRP1 construct variants. Full length LRP1 (600 kDa) consisting of the extracellular α-chain and the intracellular ß-chain was truncated to mLRP1 Domain IV (205 kDa). This construct is composed of a complete ß-chain as well as only cluster domain IV of the LRP1’s α-chain. mLRP1 Domain IV was processed to mLRP1_DIV* (120 kDa) by further truncating DIV and by the addition of a Myc- and HA tag at the N- and C-terminus, respectively

The mLRP1_DIV* transgene is a truncated version of the human LRP1 receptor. The mLRP1_DIV* mini-receptor contains a specific signal peptide (residues 1–19; amino acid sequence of the entry no. Q07954 in the UniProt database), the first five amino acids of the mature protein (*5AA linker sequence*; residues 20–24), a truncated ligand binding domain IV (residues: 3739–3778), C-terminus of 515 kDa subunit (α-chain; residues 3779–3943) and a full 85 kDa ß-subunit of human LRP1 receptor (β-chain; residues 3944–4544). Here, the generated mini LRP1 receptor construct mLRP1_DIV* consists of a truncated LRP1 DIV, a complete β-chain subunit as well as Myc- and HA tag at the N- and C-terminus, respectively (Fig. 1). Further details about mLRP1_DIV* can be found in the supplementary Data files 2 and 3.

### Cell culture

For transport experiments, the immortalized mouse brain capillary endothelial cell line bEnd.3 or the human brain microvascular endothelial cells hcMEC/D3 was used. For bEnd.3 cells, DMEM, high glucose (Lonza) containing 10% (v/v) fetal bovine serum, 100 U/ml penicillin and 100 µg/ml streptomycin (all from Gibco) was used. hcMEC/D3 cells were cultured in Endothelial Cell Basal Medium-2 (EBM^TM^-2) Bulletkit (Lonza). Epithelial CHO 13-5-1 mLRP1_DIV*cell line was used for uptake assays with 9E10. CHO PS70 cells overexpressing human wild type APP751 and human PSEN1 were used for co-culture experiments [41]. α-MEM (Lonza) supplemented with 10% (/v) fetal bovine serum, 100 U/ml penicillin and 100 µg/ml streptomycin was used for CHO cell cultivation. All cells were cultured at 37 °C and 5% C_2_.

### Transfection of endothelial cells

bEnd.3 and hcMEC/D3 cells were transfected with mLRP1_DIV* or pLBCX using Lipofectamine 3000 according to manufacturer’s instructions (L3000001, ThermoFisher). Thereby, pLBCX represents an empty vector backbone control without transgene insertion. Further details on pLBCX or mLRP1_DIV* can be found in the supplementary data files 2–5.

### Protein extraction, SDS-PAGE and immunoblotting

Cells were mechanically detached and lysed with cell lysis buffer [50 mmol/l Tris, 150 mmol/l NaCl, 0.02% (w/v) NaN_3_, 1% (v/v) Nonidet P-40 supplemented with EDTA-free protease inhibitor cocktail (cOmplete™, Roche Applied Science)]. Protein concentrations of the lysates were measured using a BCA assay according to the manufacturer’s protocol (23227, ThermoFisher). SDS-Page and immunoblotting were performed with 20 µg of protein. Densitometric analyses of immunoblotting signals were performed using the ImageJ software (Version 1.52 q) followed by protein level normalization by using β-Actin or α-tubulin signal intensities.

### Production of liposomes

#### Preparation of liposomes

For unloaded liposomes, stock solutions of EPC, Cholesterol, DSPE-PEG_2000_, 18:1 Liss Rhod PE and DSPE-PEG_2000_-Maleimide in CHCl_3_:MeOH (9:1 v/v) were mixed in molar ratios of 59:35:4,94:0,1:0,06. For BB25 immunoliposomes, 2 mg of BB25 were added to the lipid mixture. Organic solvents were removed at 50 °C under continuous nitrogen flow for 30 min and lipid films were dried under reduced pressure at room temperature for 1 h. After the addition of silica beads, lipid films were rehydrated with DPBS and centrifuged in a ZentriMix 380 R dual centrifuge (Hettich AG, Baech, Switzerland). The final lipid concentration was 100 mM.

#### Antibody thiolation & coupling

Thiolation of anti-Myc 9E10 antibody was performed for 30 min at RT using 10x molar excess of SATA. Detachment of the protection group was achieved with 0,5 M hydroxylamine-HCl over 2 h and the free protection group was removed using Zeba spin desalting columns (Thermo Fisher Scienctific, Zug, Switzerland). Deprotected antibodies were subsequently added to the previously prepared liposomes and coupling occurred over the course of 2 h at RT. Any remaining free maleimide groups were blocked with the addition of 10x molar excess of L-cysteine. For co-culture experiments, BB25–9E10 – IL or unloaded 9E10 – IL were purified directly before use using a Sepharose CL-4B filled Econo-Pac^®^ Chromatography column. The liposomes in this publication are named 9E10 functionalized immunoliposomes (9E10 - IL), unmodified immunoliposomes (unm. - IL), BB25 loaded 9E10 functionalized liposomes (BB25–9E10 - IL) or unloaded 9E10 functionalized liposomes.

(unloaded 9E10 - IL).

### In vitro transcytosis studies

Prior to examination of transcellular transport of anti-Myc antibodies or liposomes, transfected bEnd.3 or hcMEC/D3 cells were seeded into transparent membrane inserts (0.4/1 µm) coated with the coating solution. The next day, inserts were placed into the automated cell monitoring system cellZscope (NanoAnalytics) to monitor the transendothelial electrical resistance (TEER) and capacitance (CCI) of the cells. As CCI reached a value of ∼ 1 µF/cm^2^ or below, cells were stimulated with hydrocortisone (550nM) to enhance tight junction formation. When tight junction formation, barrier function and confluence of the cell monolayer was ensured (TEER > 30 Ω*cm^2^; CCI = ∼ 1 µF/cm^2^), the transport of anti-Myc antibodies was performed, approximately 72 h post transfection. Cells were incubated with the antibodies at 37 °C for 1 h. After incubation, cells were placed on ice to stop the transcellular transport. The medium of the abluminal compartment of all wells was collected and proteins within the abluminal and luminal medium were TCA precipitated. To detach the antibodies bound to the cell surface, cells were washed with acidic DPBS (pH 2) 2x for 5 min. The presence of anti-Myc antibodies in the abluminal compartment was analyzed using SDS-PAGE and immunoblotting.

Transport of 9E10 – IL or unm. – IL (3mM) was performed approximately 72 h post transfection for 2 h. As paracellular leakage marker 50 µg/ml of fluorescein isothiocyanate (FITC)-Dextran (3–4 kDa) was used. As a readout, the medium of the abluminal compartment of all wells was collected and analyzed using fluorescence spectroscopy of rhodamine or FITC by the Varioskan LUX multimode microplate reader (SkanIt Software 6.0.2.3) (Ex.: 540 nm / 495 nm, Em.: 591 nm/ 520 nm). For immunofluorescence, hcMEC/D3 cells were transfected with mLRP1_DIV* or control and seeded 24 h later into transparent membrane inserts coated with the coating solution. As CCI reached a value of ∼ 1 µF/cm^2^ or below, cells were stimulated as described above. Transport of 9E10, mIgG (30 µg/ml), 9E10 – IL or unm. - IL (3mM) was performed approximately 72 h post transfection for 1–2 h.

### In vitro transcytosis studies with Rab27a inhibitor

Prior to examination of transcellular transport of anti-Myc antibodies (Alexa Fluor™ 555 (9E10)) or 9E10 liposomes, transfected bEnd.3 or hcMEC/D3 cells were seeded into transparent membrane inserts (1 μm) coated with the coating solution. The next day, inserts were placed into the automated cell monitoring system cellZscope (NanoAnalytics) to monitor the transendothelial electrical resistance (TEER) and capacitance (CCI) of the cells. As CCI reached a value of ∼ 1 µF/cm^2^ or below, cells were stimulated with hydrocortisone (550nM) to enhance tight junction formation. When tight junction formation, barrier function and confluence of the cell monolayer was ensured (TEER > 30 Ω*cm^2^; CCI = ∼ 1 µF/cm^2^), mLRP1_DIV* transfected cells were incubated with the Rab27a inhibitor (10µM) (Nexinhib20; R&D Systems; REF 6089) for 2 h. The transport of cargo was performed, approximately 72 h post transfection with anti-Myc Alexa Fluor™ 555 antibodies (2µg/ml) or with 9E10 – IL (3mM) for 2 h. As paracellular leakage marker 50 µg/ml of fluorescein isothiocyanate (FITC)-Dextran (3–4 kDa) was used. As a readout, the medium of the abluminal compartment of all wells was collected and analyzed using fluorescence spectroscopy of rhodamine, FITC or Alexa Fluor 555 by the Varioskan LUX multimode microplate reader (SkanIt Software 6.0.2.3) (Ex.: 540 nm / 495 nm / 555 nm, Em.: 591 nm/ 520 nm / 568 nm).

### Immunofluorescence of endothelial cells

After transport, cells were washed twice with acidic DPBS and fixed in 4% PFA for 15 min. Afterwards, cells were washed with DPBS and membranes of cell culture inserts were cut out using a scalpel. Membranes were placed into a fresh 24 well plate, where cells were permeabilized in 0.1% Triton X-100 for 10 min and blocked under gentle agitation for 1 h at RT. Cells were incubated with primary antibodies on a shaker at 4 °C overnight. The next day, cells were rinsed in PBS 0.05% Triton X-100 and incubated with secondary antibodies for 1 h at RT. Next, cells were washed in PBS and dH_2_O and stained for nuclei using DAPI for 5 min. Inserts were transferred on superfrost microscope glass slides (#J1810AMNZ, ThermoFisher) and covered with coverslips using DAKO mounting solution (#83023, Dako). For abluminal immunostainings, confocal microscopy was performed using STELLARIS 8 FALCON (Leica Microsystems, Wetzlar, Germany) confocal system equipped with White Light Laser (WLL). Images were acquired with HC PL APO CS2 100x/1.40 OIL objective by using 1024 × 1024 pixel format with pixel sizes of 41 nm. Images were processed with LIGHTNING™ adaptive deconvolution (Leica) using default settings (embedding medium refractive index set to 1.47). All confocal images were prepared using Fiji distribution of ImageJ [42]. Microscope slides were stored at 4 °C.

### bEnd.3/ PS70 co-culture model

To confirm mLRP1_DIV* as transport carrier and its ability to transport the liposomal γ-secretase modulator BB25, a co-culture model was established. bEnd.3 cells were transfected with mLRP1_DIV* and seeded on coated cell culture inserts, followed by TEER and CCI monitoring. At the time of TJ stimulation, CHO cells overexpressing wild type human APP751 and human presenilin 1 (PS70 cells) were seeded on 24-well plates. bEnd.3 mLRP1_DIV* cells were divided into four groups similar in TEER and CCI values. The luminal culture media was supplemented with 50 µg/mL (FITC)-Dextran (3–4 kDa) for 24 h. To assess paracellular leakage across the endothelial monolayer and confirm a tight barrier, fluorescence intensities of FITC-dextran in the abluminal compartments were measured 24 h later as described before. After those 24 h, culture inserts with formed and stimulated bEnd.3 cell monolayer were transferred to the wells of the 24-well plates, resulting in a defined luminal compartment and abluminal compartment containing the PS70 cells. Luminal culture media was supplemented with 10µM free BB25, liposomal BB25 (BB25–9E10 - IL), unloaded 9E10 functionalized liposomes (unloaded 9E10 - IL) or DMSO. The administered concentration of the liposomes was adjusted to the free BB25. Transport was performed for 2 h. Afterwards, the amount of transported BB25–9E10 – IL or unloaded 9E10 - IL was investigated. Therefore, 100 µl medium of abluminal compartment was collected 2 h post transport and analyzed using fluorescence spectroscopy of rhodamine by the Varioskan LUX multimode microplate reader (SkanIt Software 6.0.2.3) (Ex.: 540 nm, Em.: 591 nm). After transport, luminal media was replaced by regular culture media. After 48 h, cell culture supernatants of abluminal cultured PS70 cells were collected. The γ-secretase activity was measured by determining the levels of Aβ_38_ and Aβ_42_ using a cell-based sandwich enzyme-linked immunosorbent assay (ELISA).

### Aß specific ELISA

Aβ_38_ and Aβ_42_ peptide levels in cell culture supernatants were quantified using a cell-based ELISA assay as described [39].

### Isolation of cerebral microvessels

Murine brain capillaries were isolated based on the dextran gradient centrifugation method followed by a cell-strainer filtration described elsewhere with some modifications [43, 44]. To begin with, cerebral cortices were isolated and devoid of leptomeninges by rolling on blotting paper (Whitman). Next, cortices were fragmentated in ice-cold homogenization buffer (DPBS; 2.5 mM CaCl2; 1.2 mM MgSO4; 15 mM HEPES; 25 mM NaHCO3; 10 mM glucose; 1 mM sodium pyruvate) using a Dounce tissue grinder and centrifuged at 1,000 g for 10 min at 4 °C. Resulting pellet was then thoroughly resuspended in 18% Dextrn/PBS solution (70 kDa, Sigma). The samples were centrifuged at 4000 g for 20 min at 4 °C. Red capillary pellet at the bottom of the tube was collected and filtered through.

40-µm cell nylon-mesh strainer (#352340, Corning). After through washing with ice-cold PBS, vessels remaining on the top of the mesh were collected in 1% BSA/PBS solution and pelleted by centrifugation at 4000 g for 12 min at 4 °C. Samples were lysed in microvessels lysis buffer (50 mM HEPES, pH 7.5; 1% (vol/vol) Triton X-100; 0.5% (wt/vol) sodium deoxycholate; 0.1% (wt/vol) sodium dodecyl sulphate (SDS); 500 mM NaCl; 10 mM MgCl2; 50 mM β-glycerophosphate; 1x protease inhibitor cocktail (cOmplete™); 1x phosphatase inhibitor cocktail (PhosStop™) and used for immunoblot analysis. Alternatively, for immunohistochemistry analysis, capillaries collected at the mesh were fixed with 4% Roti^®^ Histofix for 10 min and collected in 1% BSA/PBS by centrifugation at 4000 g for 20 min at 4 °C. Next, vessels were permeabilized and probed with appropriate primary and Alexa-conjugated secondary antibodies, as described previously [44].

### Immunofluorescence (IF) assay - free-floating sections

PFA-perfused mouse brains were embedded in Tissue Freezing Medium (Leica) followed by sectioning using CM3050S cryostat (Leica). 40 µm- thick slices were transferred into 48-well plates filled with cryoprotective solution (25% glycerol, 25% polyethylene glycol, 50% PBS) and stored at 4°C until further use. For IF assay, sections were first transferred to 24-well plate and washed 3 × 5 min with TBS. For mLRP1_DIV* and neurovascular unit components staining, sections were treated with 90% formic acid for 2–5 min and immediately transferred to wells filled with 0.3% Triton X-100/TBS (TBS-Tx) solution for permeabilization. Next, unspecific binding sites were blocked using a 5% (w/v) BSA in TBS-Tx for 1 h at RT. Sections were then incubated with primary antibody overnight at 4°C. On the next day, slices were washed 3 × 5 min in TBS-Tx and subjected to 1 h incubation with fluorophore-conjugated secondary antibody at RT, protected from light. Afterwards, slices were washed 1 × 5min in TBS and nuclei were counterstained with 4’,6-diamidino-2-phenylindole (DAPI) (0.2 µg/mL). Finally, sections were mounted on Superfrost Plus microscope slides (#J1810AMNZ, ThermoFisher) using fluorescent mounting medium (#83023, Dako) and dried for at least 3 h at RT before image analysis.

### AAV production

For AAV preparation, pAAV-CMV-teto2-LUC expression cassette containing luciferase reporter gene under control of the cytomegalovirus (CMV) promoter and SV40 poly-A signal embedded between two modified AAV2 Internal Terminal Repeats (ITR) was used as a vector backbone [40]. Briefly, luciferase reporter gene was removed by restriction digestion with PmeI and XbaI restriction enzymes. Subsequently, restriction sites PmeI and XbaI were introduced upstream and downstream of mLRP1_DIV*, respectively, during the PCR amplification to enable ligation. Lastly, a short phosphorylated ssDNA oligomer encoding for the hemagglutinin (HA) or Myc epitope was inserted downstream or upstream of the mLRP1_DIV* construct between last base pair (bp) of LRP1’s cytoplasmatic tail and the stop codon or after the leader sequence N-terminal using HiFi DNA Assembly Master Mix (NEB). DNA was transformed into chemically competent NEB^®^ Stable Competent E. coli (#C3040I, NEB) according to the manufacturer’s protocol. Further details on AAV production can be found in the supplementary Data files 1, 6–9.

### AAV expression in mice

To express the mLRP1_DIV* construct into the brain endothelium, 2-month-old 5xFAD *wt* females were injected intravenously with AAV(BR1)mLRP1_DIV* virus (1 × 10^11^ genomic particles per mouse). Virus titer was determined by quantitative real-time PCR after purification as described previously using CMV-specific primers: CMV forward 5’ GGG ACT TTC CTA CTT GGC A 3’; CMV reverse 5’ GGC GGA GTT ACG ACA T 3’ [40]. Animals were of similar weight, randomly allocated to treated and control groups. Briefly, a mouse was placed in a restrainer and the tail was warmed up with infrared lamp for several minutes to facilitate vasodilatation of the tail veins. Recombinant AAV aliquots (max vol = 100 µL) were administered into left or right tail vein. Animals were kept under daily supervision for the next two weeks and were sacrificed after 12–16 weeks.

### Statistical analysis

For statistical analysis GraphPad Prism (8.4.3) was used. Means and standard error of the mean (SEM) were calculated for all groups and presented graphically. For the evaluation of the statistical significance unpaired t-test or one-way ANOVA followed by Tukey’s multiple comparison test were used, whereby significance limit was *p* < 0.05. Significant differences are marked. For all experiments, all technical replicates from *n* = 3 independent experiments were used for statistical analysis and are presented graphically. Images from LSM710 or 8 Falcon stellaris microscopes were arranged and adjusted using the ImageJ software (Version 2.1.0/1.53c) and Microsoft PowerPoint 365. Final images were arranged with CorelDraw2024. Schematic illustrations were created with bioRender.com.

## Results

Here, the truncated mini low-density lipoprotein receptor-related protein 1 mLRP1_DIV* is presented as blood to brain transport carrier, exemplified by antibodies and immunoliposomes using a systematic approach to screen the receptor and its ligands’ route across endothelial cells *in vitro.* The use of mLRP1_DIV* as liposomal carrier into the CNS was validated based on transport assays across an in vitro model of the BBB using hcMEC/D3 and bEnd.3 cells. Trafficking routes of mLRP1_DIV* and corresponding cargo across endothelial cells were analyzed using immunofluorescence. The transport of liposomes loaded with the GSM BB25 across bEnd.3 mLRP1_DIV* cells and their ability to modulate γ-secretase activity was investigated in co-culture with CHO cells overexpressing APP and PSEN1.

(PS70 cells).

### mLRP1_DIV* mediated transcellular transport of anti-myc antibodies across an in vitro model of the BBB

**Fig. 2:**
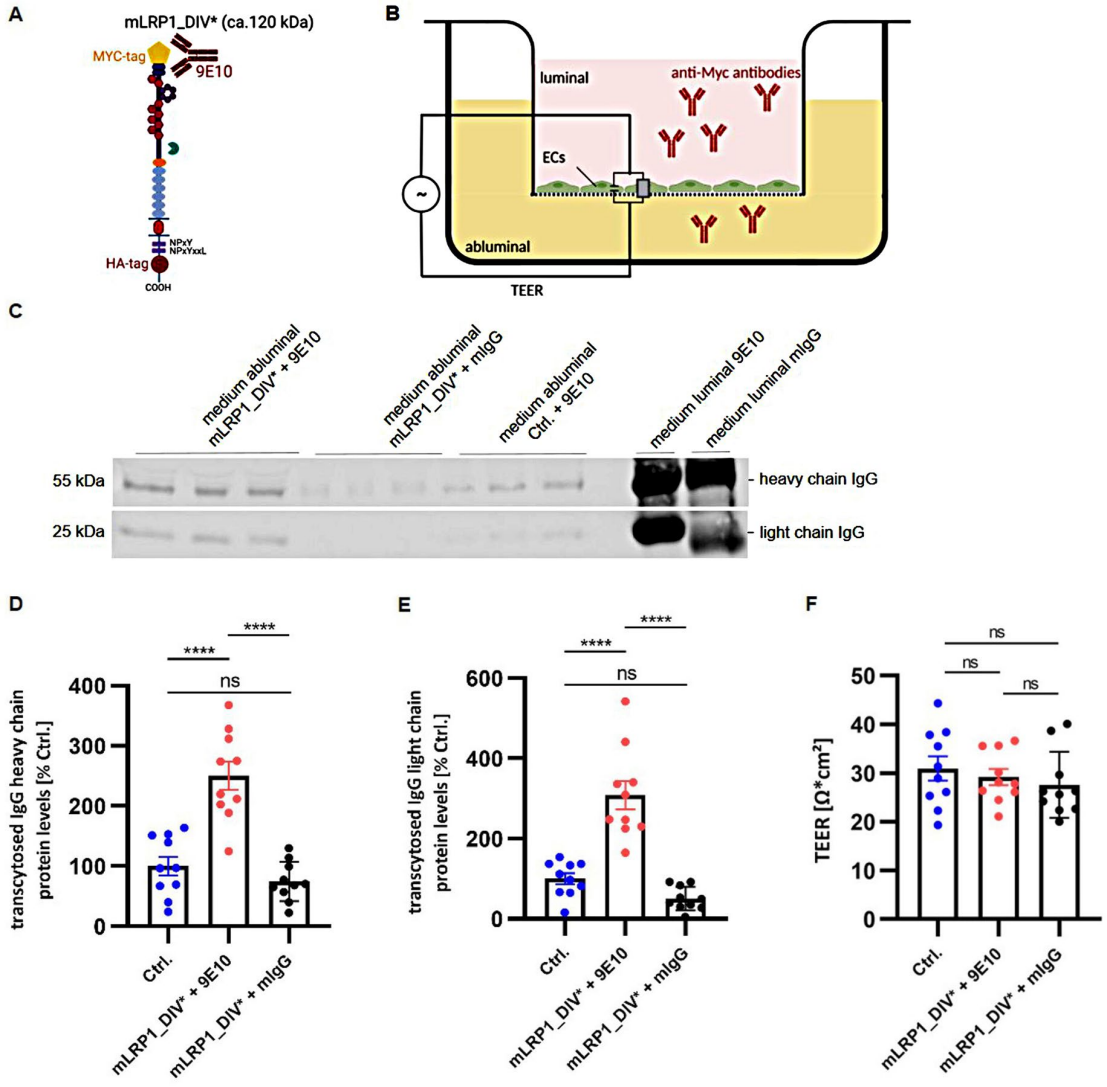
mLRP1_DIV* mediated transcellular transport of anti-Myc antibodies across hcMEC/D3 cells. **(A)** Schematic illustration of mLRP1_DIV* **(B)** Schematic illustration of the experimental setup. Representative immunoblotting for **(C)** luminal and abluminal medium of hcMEC/D3 being transiently transfected with mLRP1_DIV* or pLBCX. At confluence, cells were incubated with 30 µg/ml anti-Myc antibodies (9E10) or unspecific mIgG for 1 h. **(D, E)** Heavy and light chain protein levels were quantified by densitometric analysis after immunoblotting and normalized to luminal medium saved before transport. The intensity of 9E10 in abluminal medium of hcMEC/D3 pLBCX cells were defined as 100%. **(F)** TEER at the time of transport was measured by impedance spectroscopy. Data represent the mean ± SEM of ten individual replicates from *n* = 3 independent experiments. One-way ANOVA followed by Tukey’s multiple comparison test was used for statistical analysis

In our first studies mLRP1_DIV* (Fig. 2A) was demonstrated to specifically internalize anti-Myc antibodies in CHO 13-5-1 cells (Supplementary Data 1, Figure S3). Therefore, the mLRP1_DIV* construct was analyzed in the human derived endothelial cell line hcMEC/D3, as these cells consists of a luminal and abluminal polarization. For examination of mLRP1_DIV*’s functionality, an in vitro model of the BBB, which enables to monitor tight junction and barrier formation of the endothelial cells, was used.

Cells were transfected with mLRP1_DIV* or pLBCX and 30 µg/ml of 9E10 or unspecific mouse IgG (mIgG) were luminally applied to the cell monolayer in the in vitro BBB model for 1 h (Fig. 2B). Respective protein amounts were analyzed in the abluminal medium. Regarding transport assay in hcMEC/D3 cells, two bands at about 50 kDa and 25 kDa, corresponding to IgG, became visible in abluminal and luminal medium of cells being transfected with pLBCX or mLRP1_DIV* (Fig. 2C). According to transport of 9E10 in hcMEC/D3 cells, cells transfected with mLRP1_DIV* showed an average transport of 9E10’s heavy chain of 15% after 60 min whereas hcMEC/D3 pLBCX cells showed an average transport of 9E10’s heavy chain of 5.8%. Transport of unspecific mIgG in cells transfected with mLRP1_DIV* averaged out at 4.2% (Fig. 2D). Thereby, a significant higher transcytosis.

(2.5-fold/ 3.3-fold) of 9E10’s heavy chain in hcMEC/D3 mLRP1_DIV* cells could be observed compared to control or unspecific mIgG (*p* < 0.0001 / *p* < 0.0001). The transport of the light chain of 9E10 averaged out at 8% after 60 min using hcMEC/D3 mLRP1_DIV* cells and 2.4% using hcMEC/D3 pLBCX cells. mLRP1_DIV* transfected cells showed a transport of unspecific mIgG of around 1.3% (Fig. 2E). Cells expressing the mLRP1_DIV* receptor showed a significant higher transport (3-fold/ 6-fold) of 9E10’s light chain compared to control or mIgG (*p* < 0.0001 / *p* < 0.0001). Notably, no significant differences could be observed between transport of 9E10 in control cells and transport of mIgG in hcMEC/D3 mLRP1_DIV* cells (*p* = 0.55 / *p* = 0.29). Moreover, no difference in the BBB’s integrity could be observed between the experimental groups, as the transendothelial electrical resistance of hcMEC/D3 pLBCX was about 30.9 Ω*cm^2^ and 28.4 Ω*cm^2^ using hcMEC/D3 mLRP1_DIV* cells (*p* = 0.83 / *p* = 0.52 / *p* = 0.85) (Fig. 2F). Together, data clearly demonstrate mLRP1_DIV* mediated transport of 9E10 across an in vitro model of the BBB.

### Luminal to abluminal transport of anti-myc antibodies across hcMEC/D3 cells

**Fig. 3:**
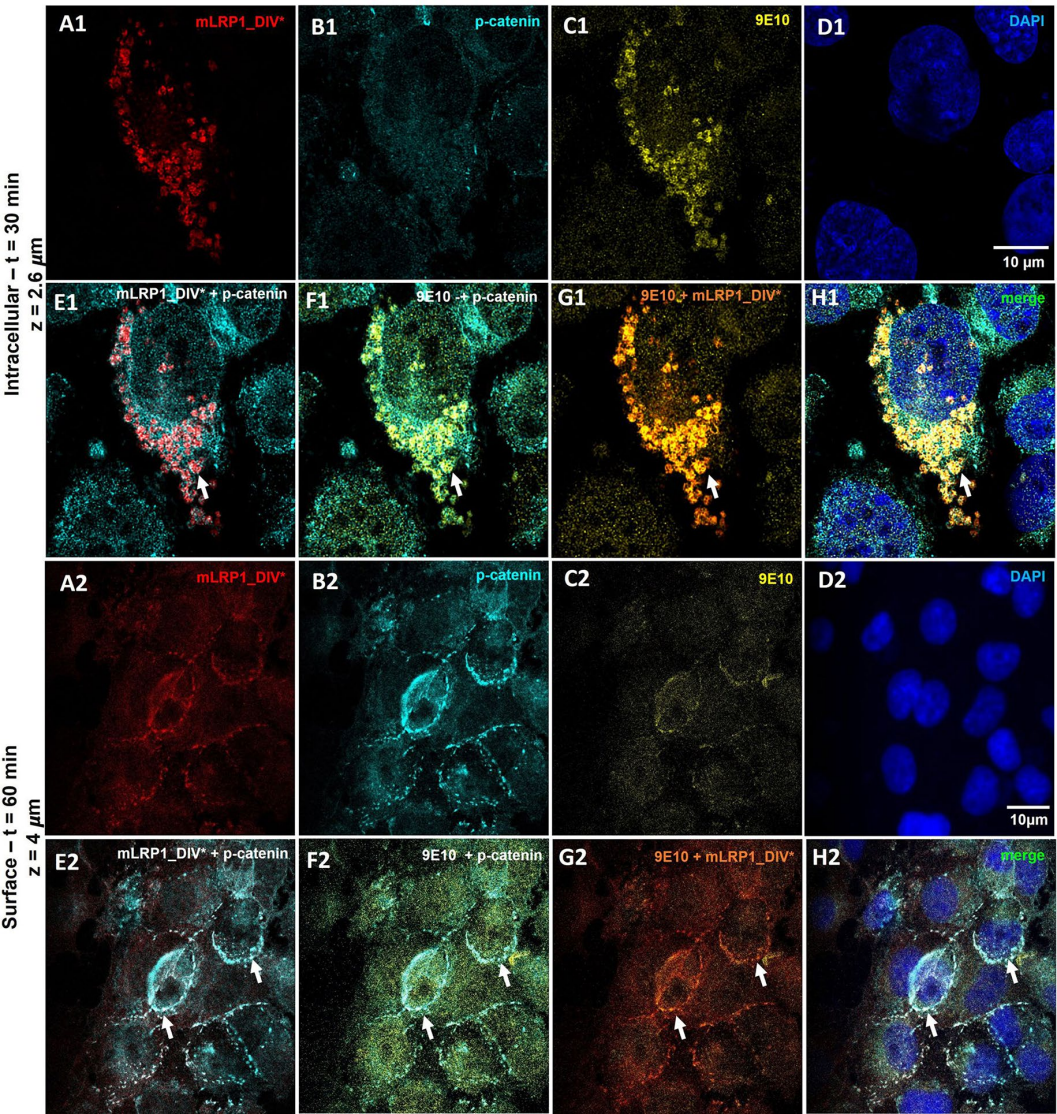
Basolateral sorting of 9E10/mLRP1_DIV* complex in co-stainings with p-catenin during transport across hcMEC/D3 cells. Cells were transfected with mLRP1_DIV* and transport of 9E10 was performed 72 h post transfection. Representative confocal images of **(A)** mLRP1_DIV*, **(B)** abluminal marker p-catenin, **(C)** 9E10, **(D)** nuclei and co-localization **(E - H)** 30 min (1) and 60 min (2) after incubation with AlexaFluor488-9E10. Cells were washed with acidic PBS, fixed with 4% PFA, permeabilized and stained for mLRP1_DIV* and p-catenin. Images were taken with the Stellaris 8 Falcon confocal laser scanning microscope using a laser at a wavelength of (mLRP1_DIV*) 647 nm, (9E10) 488 nm, (p-catenin) 568 nm and (nuclei) 350 nm. mLRP1_DIV* is depicted in red, 9E10 in yellow, p-catenin in cyan and nuclei in blue. Co-localizations were investigated by **(E)** merge of A and B, **(F)** merge of B and C, **(G)** merge of A and C. Scale bar = 10 μm, z = depth in the cell

As demonstrated above, 9E10 antibodies are transported via the truncated mini LRP1 receptor across human endothelial cells. In the following, the vesicular trafficking route of anti-Myc antibodies (9E10) was further investigated. Cells were co-stained for mLRP1_DIV*, the endocytosis marker Clathrin and Caveolin-1, the early endosome using EEA-1, the lysosome via Lamp-1, recycling endosomes using TfR-1 and for the exocytosis marker Rab27a [45–48]. After entering the cell via a Clathrin – and caveolin-mediated endocytosis, the receptor-antibody complex seems to be routed directly across the cells into recycling endosomes followed by exocytosis via Rab27a positive vesicles (Supplementary Data 1, Figure S4). However, co-localization with Rab27a-positive structures only indicates exocytosis of antibodies in general but cannot allow further specification regarding the side of the release. As 9E10 antibodies should be fused to liposomes as tool for drug delivery into the CNS, a release of the antibodies on the basolateral side must be ensured. Therefore, abluminal exocytosis of 9E10 was further validated by immunofluorescence. Like experiments before, mLRP1_DIV* was transfected into hcMEC/D3 cells, which were then grown in transmembrane inserts until a monolayer was formed. By exposing the luminal side of the cells to 9E10 for different time periods, the abluminal location and release of 9E10 and mLRP1_DIV* was investigated by a co-localization with p-catenin. Besides tight junctions, a different class of junction proteins known as adherens, including cadherins, catenins and junctional adhesion molecules (JAMs) are involved in the formation, stabilization, and organization of the intercellular junctions at the endothelium, mostly at the basolateral membrane [49, 50]. Regarding immunostainings, only a co-localization between mLRP1_DIV* and 9E10, not between mLRP1_DIV* and p-catenin or 9E10 and p-catenin, could be shown within the cell following a 30-minute incubation of hcMEC/D3 mLRP1_DIV* cells with 9E10 antibodies (Fig. 3E1- G1). However, mLRP1_DIV* and 9E10 antibodies were visible at the cell surface along with p-catenin after a 60-minute incubation (Fig. 3E2 - H2). Co-localization of both, mLRP1_DIV* and 9E10 with p-catenin confirms mLRP1_DIV* dependent release of 9E10 on the abluminal side of hcMEC/D3 cells.

### mLRP1_DIV* mediated transcellular transport of 9E10 functionalized immunoliposomes across an in vitro model of the BBB

**Fig. 4:**
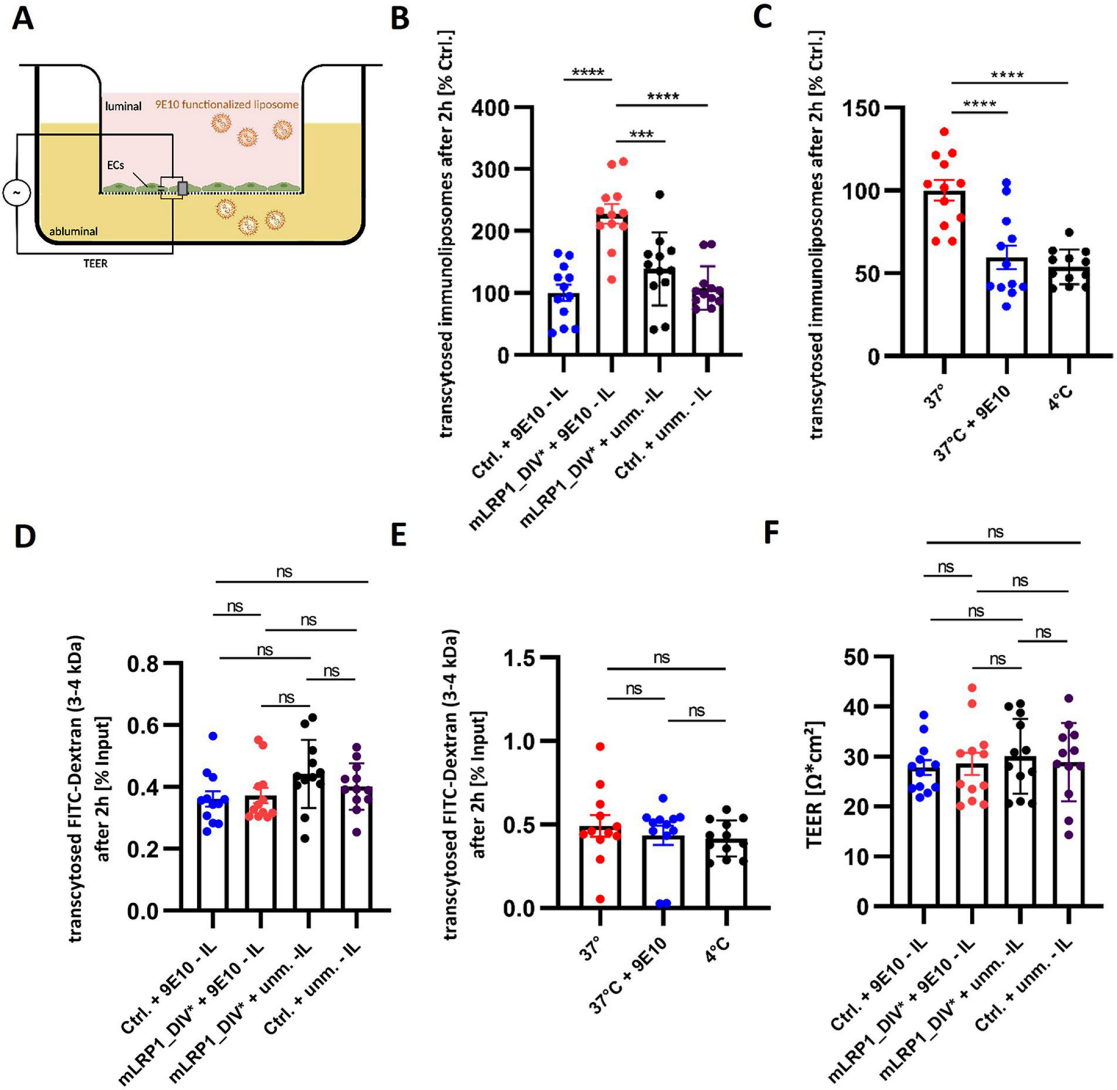
mLRP1_DIV* mediated transcellular transport of 9E10 - IL across hcMEC/D3 cells. **(A)** Schematic illustration of the in vitro BBB model. **(B)** Cells were transiently transfected with mLRP1_DIV* or pLBCX. At confluence, cells were incubated with 3mM 9E10 functionalized or unmodified liposomes for 2 h. **(C)** Transcytosis of 3mM 9E10 functionalized liposomes in hcMEC/D3 mLRP1_DIV* cells after 2 h at 37 °C with or without 9E10 antibodies or at 4 °C. **(D** and **E)** Paracellular leakage of 50 µg/ml FITC-Dextran (3–4 kDa) in corresponding experimental groups. **(B** - **E)** Amount of transcytosed immunoliposomes and FITC-Dextran were analyzed by fluorescence measurement of rhodamine or FITC in the abluminal medium. To quantify the transport of liposomes, a calibration curve for both liposomes was generated. Transported amount of liposomes was calculated according to the calibration curve. Transcytosed amount of FITC-Dextran was calculated percentual to the input saved before the transport. **(B)** hcMEC/D3 pLBCX cells or **(C)** hcMEC/D3 mLRP1_DIV* incubated with 9E10 functionalized immunoliposomes at 37 °C were defined as 100%. **(F)** TEER at the time of transport was measured by impedance spectroscopy. Data represent the mean ± SEM of twelve individual replicates of *n* = 3 independent experiments. One-way ANOVA followed by Tukey’s multiple comparison test

Based on the results obtained in hcMEC/D3 cells concerning internalization of liposomes, mLRP1_DIV*’s functionality of transcytosing liposomes was analyzed (Supplementary Data 1, Figs. S6 – S7). Therefore, the in vitro model of the BBB, which enables to monitor tight junction, barrier formation and confluence of the endothelial cells, was used. Cells were transfected with mLRP1_DIV* or pLBCX and immunoliposomes were luminally applied for 2 h, 72 h after transfection. The amount of transported liposomes was investigated by fluorescence intensity of rhodamine in the medium of the abluminal compartment. The experimental setup is depicted in Fig. 4A. Thereby, cells expressing mLRP1_DIV* showed a significant higher transcytosis (2.28-fold, 1.65-fold, 2.1-fold) of 9E10 functionalized liposomes compared to cells lacking mLRP1_DIV* (*p* < 0.0001) and compared to an application of unmodified liposomes to mLRP1_DIV* transfected cells (*p* = 0.0003) or to control cells (*p* < 0.0001) (Fig. 4B). The transcytosis of 9E10 functionalized liposomes in mLRP1_DIV* cells was further confirmed by an incubation at 37 °C with or without 9E10 antibodies or at 4 °C to compete or inhibit the transcytosis process. Thereby, a significant increased transcytosis of ∼ 50% of 9E10 functionalized liposomes at 37 °C compared to an incubation with 9E10 antibodies (*p* < 0.0001) or at 4 °C (*p* < 0.0001) was observed (Fig. 4C). In both transcytosis experiments the integrity of the in vitro BBB was confirmed using FITC-Dextran (3–4 kDa) as permeability marker. For all experimental groups, diffusion of FITC-Dextran was less than 0.5% indicating an intact barrier (Fig. 4D and E). Moreover, no difference in the BBB’s integrity could be observed between the experimental groups, as the transendothelial electrical resistance of hcMEC/D3 pLBCX was about 30.4 Ω*cm^2^ and 29.7 Ω*cm^2^ using hcMEC/D3 mLRP1_DIV* cells (Fig. 4F).

### mLRP1_DIV* mediated luminal to abluminal transport of 9E10 functionalized liposomes across hcMEC/D3 cells

**Fig. 5:**
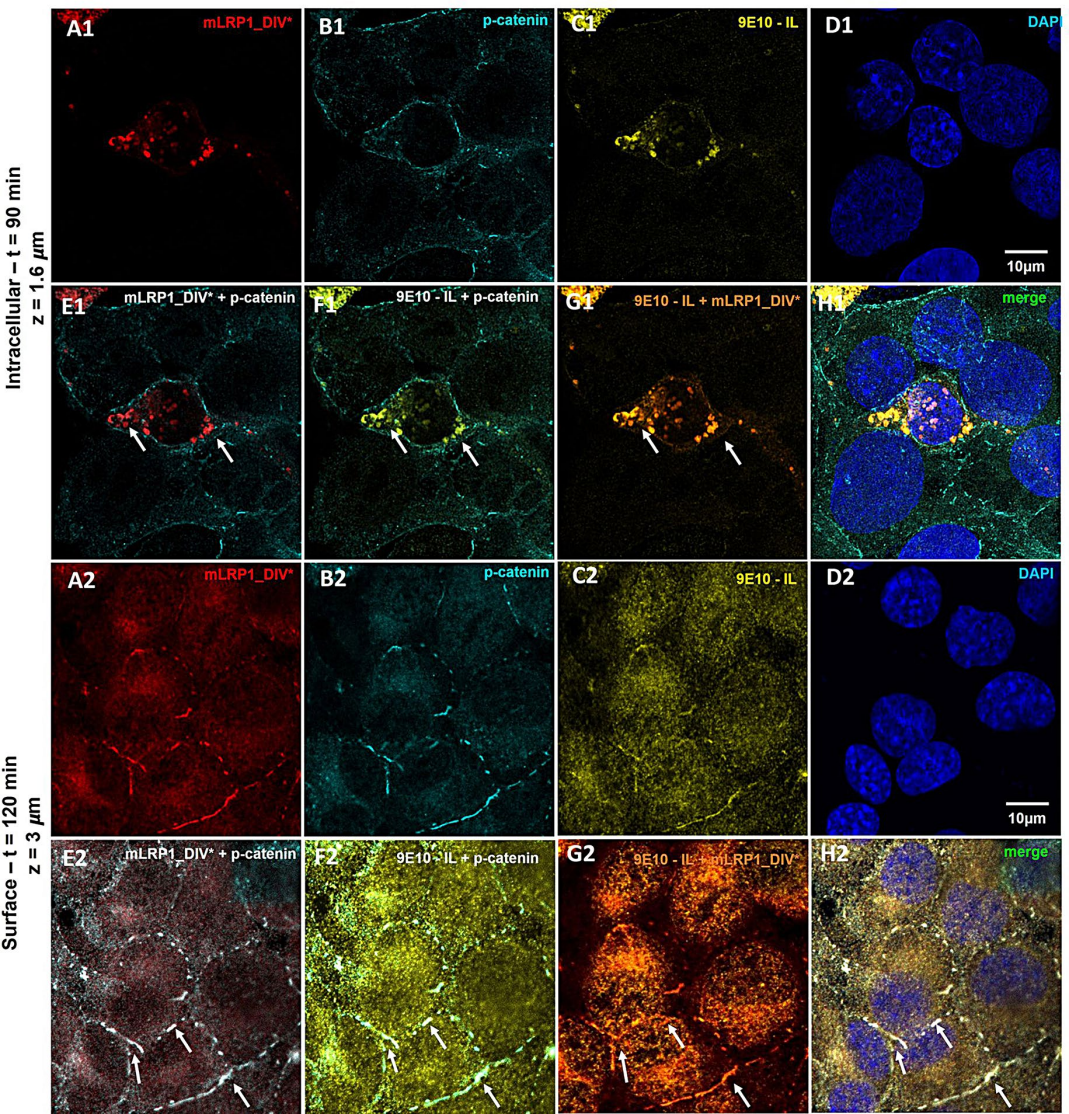
Basolateral sorting of 9E10 - IL/mLRP1_DIV* complex in co-stainings with p-catenin during transport across hcMEC/D3 cells. Cells were transfected with mLRP1_DIV* and transport of 9E10 - IL was performed 72 h post transfection. Representative confocal images of **(A)** mLRP1_DIV*, **(B)** abluminal marker p-catenin, **(C)** 9E10 - IL, **(D)** nuclei and co-localization **(E** - **H)** for (1) 90 min or (2) 120 min after incubation with the liposomes. Cells were washed with acidic PBS, fixed with 4% PFA, permeabilized and stained for mLRP1_DIV* and p-catenin. Images were taken with the Stellaris 8 Falcon confocal laser scanning microscope using a laser at a wavelength of (mLRP1_DIV*) 647 nm, (rhodamine) 540 nm, (p-catenin) 488 nm and (nuclei) 350 nm. mLRP1_DIV* is depicted in red, 9E10 - IL in yellow, p-catenin in cyan and nuclei in blue. Co-localizations were investigated by **(E)** merge of A and B, **(F)** merge of B and C, **(G)** merge of A and C, **(H)** merge of all. Scale bar = 10 μm, z = depth in the cell

As mentioned previously, the truncated mini LRP1 receptor allows 9E10 functionalized liposomes to pass through human endothelial cells (Supplementary Data 1; Figure S8-S10). After entering the cell by Clathrin- and Caveolin-mediated endocytosis, the receptor-liposome complex appears to be transported straight across the cells into recycling endosomes followed by exocytosis via Rab27a positive vesicles. Here, the basolateral sorting of 9E10 functionalized immunoliposomes was further explored by exposing the luminal side of the cells to 3 mM of 9E10 functionalized liposomes for different time periods. Co-localization of 9E10 functionalized liposomes and mLRP1_DIV* with the abluminal marker p-catenin allowed for an investigation of their abluminal location and release. After incubation of hcMEC/D3 mLRP1_DIV* cells with 9E10 functionalized liposomes for 90 min, only a co-localization between mLRP1_DIV* and liposomes could be detected within the cell, but not between mLRP1_DIV* and p-catenin or 9E10 - IL and p-catenin (Fig. 5E1 - G1). However, after an incubation of 120 min, it was possible to detect mLRP1_DIV* and 9E10 functionalized liposomes at the cell surface, together with p-catenin (Fig. 5E2 - H2). Immunofluorescent stainings clearly demonstrate mLRP1_DIV* dependent transport of 9E10 – IL across hCMEC/D3 cells, followed by their abluminal release.

### mLRP1_DIV*/cargo complex is released Rab27a dependent on the abluminal side of endothelial cells

**Fig. 6:**
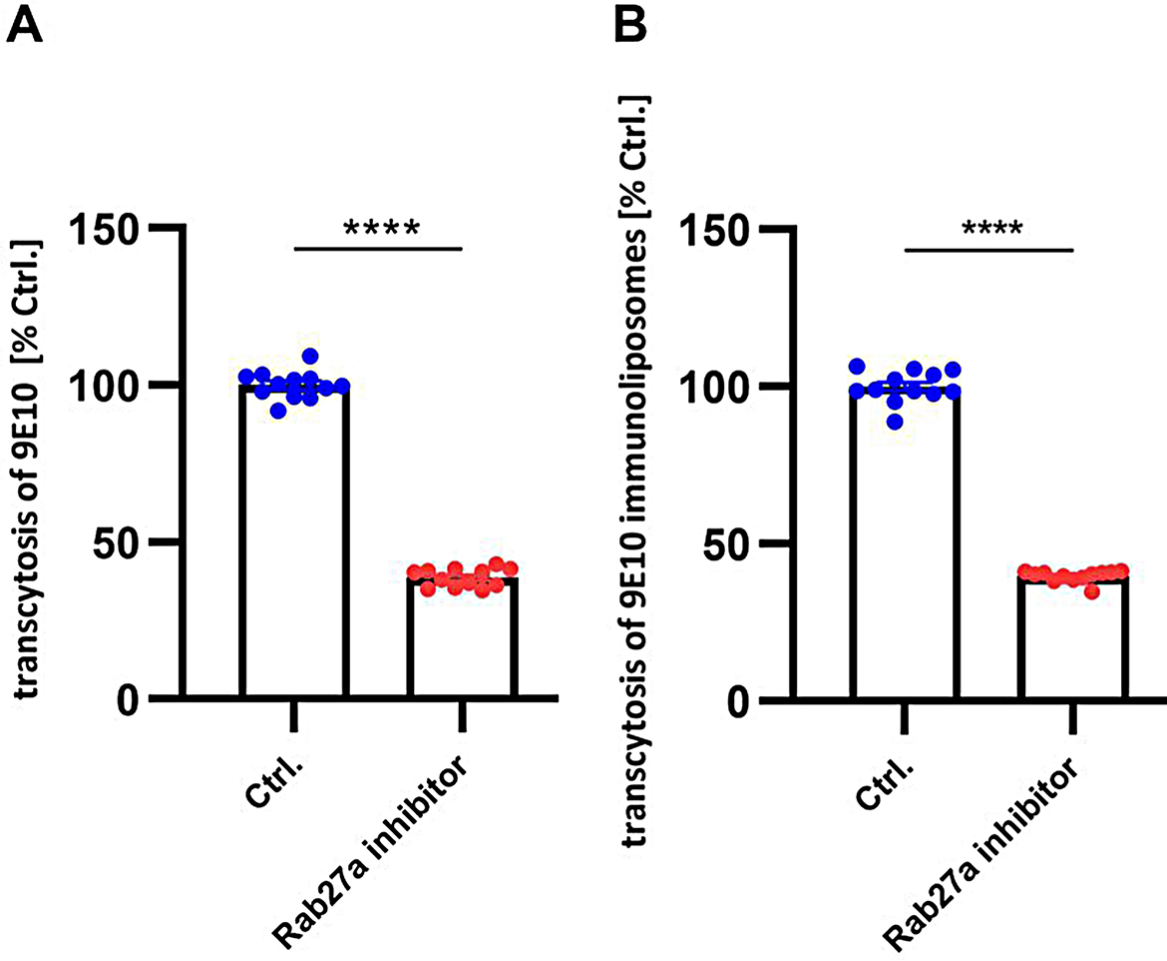
Transcytosis of 9E10 or 9E10 immunoliposomes across endothelial cells upon Rab27a inhibition. **(A)** hcMEC/D3 or **(B)** bEnd.3 cells were transiently transfected with mLRP1_DIV* and cultured in cell culture inserts in an in vitro BBB model. At confluence, cells were incubated with 10µM Rab27a inhibitor or DMSO control and transport of **(A)** 2 µg/ml of anti-Myc Alexa Fluor™ 555 or **(B)** 3mM 9E10 immunoliposomes was performed after 2 h for 2 h. Amount of transcytosed Alexa Fluor™ 555 9E10 or 9E10 immunoliposomes was analyzed using fluorescence spectroscopy. To quantify the transport of liposomes, a calibration curve for the liposomes was generated. Transported amount of liposomes was calculated according to the calibration curve. Transcytosed amount of anti-Myc Alexa Fluor™ 555 was calculated percentual to the input saved before the transport. Cells incubated with DMSO control were defined as 100% (Ctrl.). Data represent the mean ± SEM of twelve individual replicates of *n* = 3 independent experiments. Unpaired t- test was used for statistical analysis

To further explore the mechanism by which the mLRP1_DIV*/cargo complex is exocytosed on the basolateral side of endothelial cells in vitro, a transcytosis of 9E10-immunoliposomes across bEnd.3 mLRP1_DIV* cells or of 9E10 across hcMEC/D3 mLRP1_DIV* was performed with or without the Rab27a inhibitor Nexinhib20 (Fig. 6). Formed and stimulated bEnd.3/hcMEC/D3 mLRP1_DIV* cell monolayer was treated with or without 10µM Rab27a inhibitor for 2 h before and during the transcytosis of 9E10 or 9E10 immunoliposomes. Transport of both, 9E10 or 9E10 immunoliposomes was determined using fluorescence spectroscopy. Thereby, hcMEC/D3 mLRP1_DIV* cells show a 61.3% reduction in transcytosis of 9E10, when cells were incubated with the Rab27a inhibitor compared to DMSO-treated hcMEC/D3 mLRP1_DIV* cells (*p* < 0.0001) (Fig. 6A). Similar, bEnd.3 mLRP1_DIV* cells also show a 60.2% reduction in transcytosis of 9E10 immunoliposomes when cells were incubated with the Rab27a inhibitor compared to DMSO-treated bEnd.3 mLRP1_DIV* cells (*p* < 0.0001) (Fig. 6B). Importantly, no difference in the BBB’s integrity could be observed between the experimental groups, as the transendothelial electrical resistance of hcMEC/D3 was about 44.8 Ω*cm^2^ for both groups and of bEnd.3 cells about 44.7 Ω*cm^2^ for both treated and non-treated group. For all experimental groups, diffusion of the paracellular leakage marker FITC-Dextran was less than 0.7% indicating an intact barrier (Data not shown). Together data in bEnd.3 or hcMEC/D3 cells clearly demonstrate that the 9E10/mLRP1_DIV* or 9E10 immunoliposomes/ mLRP1_DIV* complex is exocytosed Rab27a dependently.

### mLRP1_DIV* mediated transport of the liposomal γ-secretase modulator BB25

#### Preparation and characterization of liposomes

Previous studies reported the small molecule BB25 as a promising γ-secretase modulator (GSM) with nanomolar potency. Following treatment of CHO cells with stable co-expression of human APP751 and PSEN1 (PS70 cells), BB25 displayed the typical characteristics of a GSM with a dose-dependent decrease in Aß_42_ levels and a concomitant increase in Aß_38_ levels [39]. To investigate the novel approach to deliver potential drugs across the BBB using the artificial mRLP1_DIV* construct, an in vitro BBB co-culture model was established. Thereby, the transport and functionality of the GSM BB25 embedded in 9E10 functionalized liposomes was analyzed. The liposomes were prepared by a thin film hydration method. The liposomes had a size between 118 and 145 nm. The amount of incorporated BB25 was approx. 4.2 mM (Table 1). The increase in size of BB25 immunoliposomes when compared to unloaded immunoliposomes likely shows the incorporation of BB25 into the liposomal membrane. However, all liposomal formulations tested in this study meet the requirements for BBB targeting nanocarriers regarding size and PDI.

**Table 1:** Physiochemical characteristics of liposomes used

Formulation	Mean particle diameter (nm)	Polydispersity index (PDI)	BB25 loading (mM)
**BB25–9E10 - IL**	144.6 nm	0.165	4.2
**unloaded 9E10 - IL**	118.1 nm	0.155	-

#### Liposomal BB25 modulated Aß peptide generation in PS70 cells after transport across bEnd.3 in a co-culture model

**Fig. 7:**
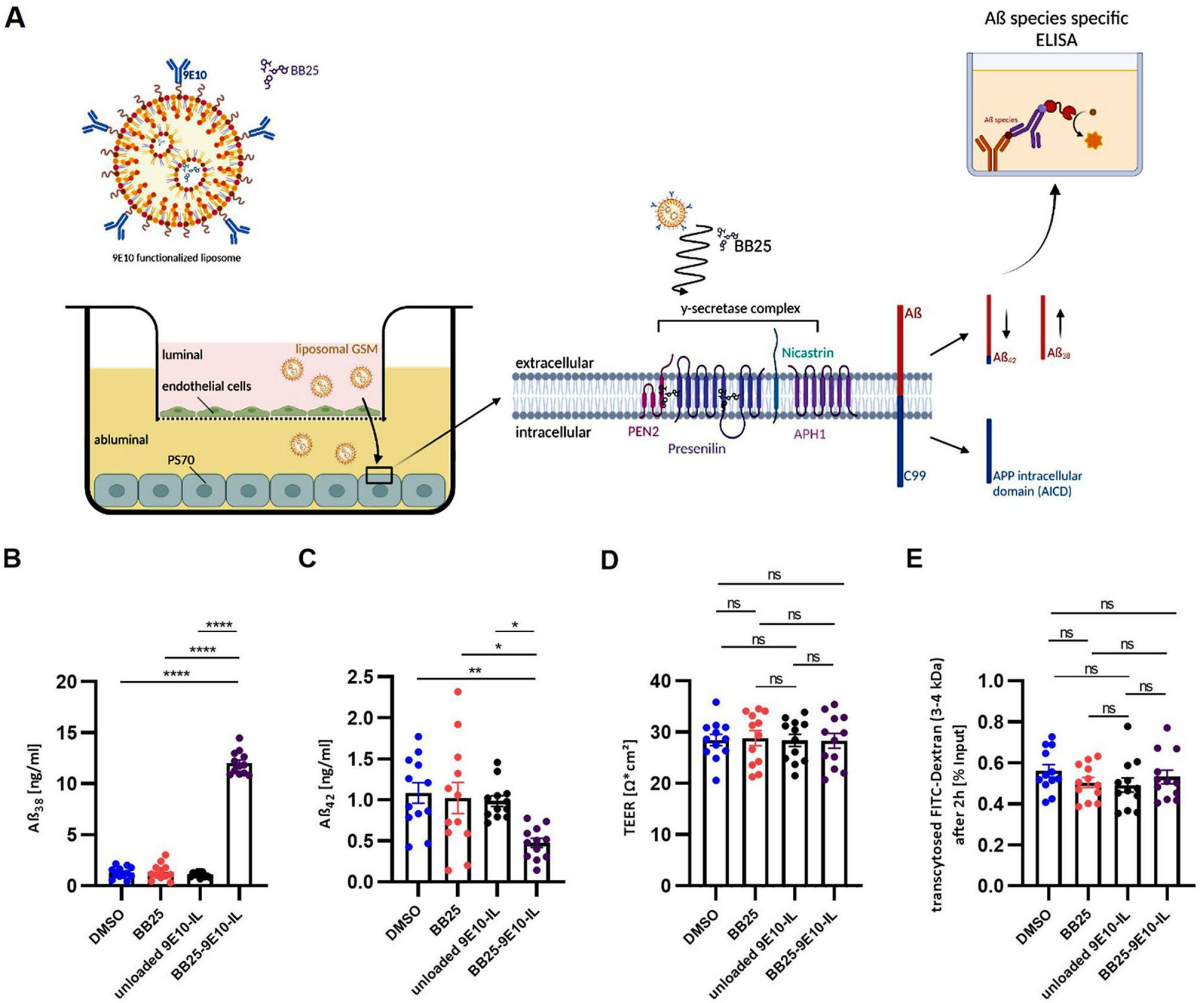
Aß levels changed after mLRP1_DIV* mediated transport of liposomal BB25 across bEnd.3 cells. **(A)** Schematic illustration of the co-culture model. PS70 cells overexpressing human APP751 and Presenilin 1 were abluminally co-cultured with transiently transfected bEnd.3 mLRP1_DIV* cells in the luminal compartment. **(B, E)** Post-confluent bEnd.3 mLRP1_DIV* cells were treated with 10µM BB25, BB25–9E10 - IL, unloaded 9E10 - IL or DMSO for 2 h. The administered concentration of the liposomes was adjusted to the free BB25. Levels of **(B)** Aβ_38_ and **(C)** Aβ_42_ in abluminal cell culture supernatants were measured 48 h post transport by an Aβ species specific ELISA. **(D)** TEER at the time of transport was measured by impedance spectroscopy. **(E)** Paracellular leakage of FITC-Dextran (3–4 kDa) 24 h before transport for 24 h. Data represent the mean ± SEM of twelve individual replicates from *n* = 3 independent experiments. One-way ANOVA followed by Tukey’s multiple comparison test was used for statistical analysis

After validation that immunoliposomes or free BB25 have no impact on endothelial barrier properties (Supplementary Data 1, Figure S15), the functionality of the mLRP1_DIV* mediated drug delivery mechanism was validated by a co-culture model, composed of luminally cultured bEnd.3 mLRP1_DIV* combined with abluminally cultured PS70 cells overexpressing APP751 and PSEN1 (Fig. 7A). When a tight barrier of bEnd.3 cells, monitored by impedance spectroscopy, was achieved, 10 µM BB25, BB25 loaded 9E10 functionalized liposomes, unloaded 9E10 functionalized liposomes, or DMSO were added to the bEnd.3 cells into the luminal compartment for 2 h. Afterwards, luminal medium was removed and replaced by normal culture medium. As a readout for a mLRP1_DIV* mediated transport of liposomal BB25 and subsequent release into the brain parenchyma, abluminal medium of PS70 cells was collected 48 h post transport and an Aβ specific ELISA was used to quantify the levels of Aβ_38_ and Aβ_42_ peptides. Furthermore, the transported amount of BB25–9E10 – IL and unloaded 9E10 - IL across the bEnd.3 mLRP1_DIV* monolayer was measured by fluorescence spectroscopy. Luminally administrated free BB25 caused no changes in Aβ_38_ and Aβ_42_ peptide levels compared to DMSO vehicle, indicating an insufficient diffusion across the bEnd.3 monolayer (*p* = 0.99 / *p* = 0.98) (Fig. 7B and C). In contrast, bEnd.3 mLRP1_DIV* cells showed a transport of liposomal BB25 of approximately

11.5 ± 0.6%, resulting in an 9.3-fold increase of Aß_38_ as well as a decrease of Aß_42_ of 56.3% compared to the DMSO control (*p* < 0.0001, *p* = 0.0051) (Fig. 7B and C; Table 2). Compared to free BB25, an 8.8-fold increase of Aß_38_ as well as a decrease of Aß_42_ of 53.7% could be detected (*p* < 0.0001,

*p* = 0 0.0141) (Fig. 7B and C). Similar results were seen after transport of liposomal BB25 compared to unloaded immunoliposomes, as a 11.1-fold increase of Aß_38_ as well as a decrease of Aß_42_ of 51.9% could be observed (*p* < 0.0001, *p* = 0.024). Although 10.9 ± 2.01% of unloaded 9E10 immunoliposomes were transcytosed, no modulating effect on γ-secretase activity with respect to Aß_38_ or Aβ_42_ peptide levels could be detected compared to an application of DMSO vehicle or free BB25 (*p* = 0.91, *p* = 0.94 / *p* = 0.79, *p* = 0.99) (Fig. 7B and C; Table 2). Notably, Aß_38_ and Aβ_42_ peptide levels averaged out at the same concentration after luminal application of DMSO, free BB25, or unloaded 9E10 liposomes across bEnd.3 mLRP1_DIV* cells (Fig. 7B and C). Importantly, all experimental groups showed similar TEER of approximately 28.58 Ω*cm^2^ as well as the same amount of paracellular diffusion of FITC-Dextran (3-4 kDa) of approximately 0.54%, indicating a comparable tight in vitro BBB between all experimental groups (Fig. 7D and E). These findings clearly demonstrate that BB25 loaded 9E10 functionalized immunoliposomes were transported mLRP1_DIV* dependently across an in vitro model of the BBB, followed by the release of BB25 on the abluminal side, where it modulated γ-secretase activity resulting in decreased Aβ_42_ and increased Aß_38_ peptide levels.

**Table 2:** Concentration of rhodamine in the abluminal compartment measured by fluorescence spectroscopy

Formulation	µM luminally administered	rhodamine abluminally 2 h post transport (µM)	% of administered concentration
BB25–9E10 – IL	184.8 µM lipid ≙ 10 µM (BB25)	21.3 µM lipid ≙ 1.15 µM (BB25)	11.5 ± 0.6%
unloaded 9E10 - IL	184.8 µM lipid	20.1 µM lipid	10.9 ± 2.01%

### AAV-(BR1)-mLRP1_DIV* is expressed in mouse brain capillaries

**Fig. 8:**
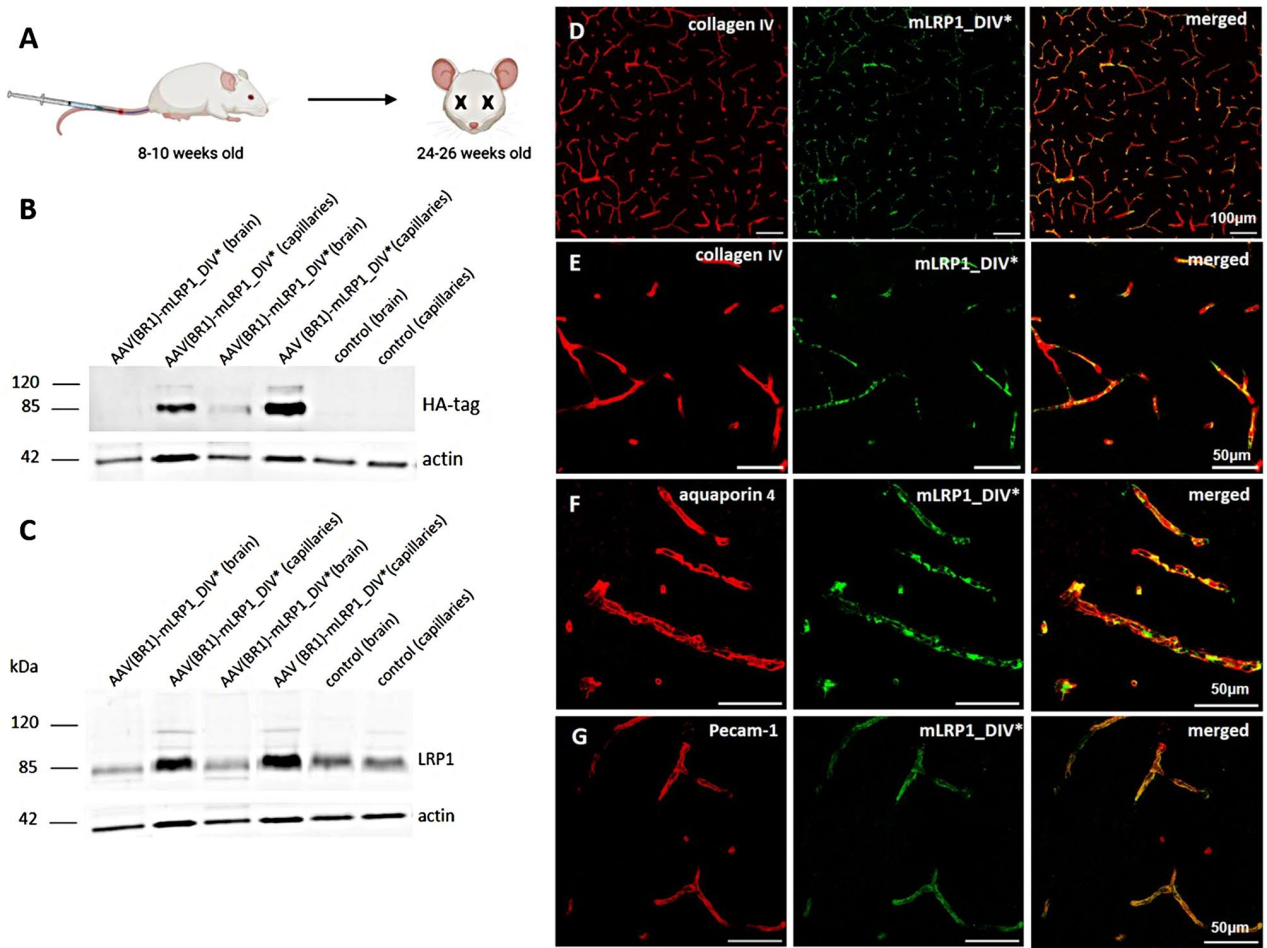
Ex vivo validation of mLRP1_DIV* expression in mouse brain tissue. **(A)** Intravenous injection of AAV(BR1)mLRP1_DIV* in 10 weeks old mice. **(B, C)** Representative immunoblotting for protein expression of mLRP1_DIV* in isolated brain microvasculature (capillaries) and vessel depleted brains of AAV treated 5xFAD wt mice and their littermate controls. ß-actin was used as loading control. Overexpression of mLRP1_DIV* was validated using **(B)** anti-HA tag antibody or **(C)** anti LRP1-ß-chain antibody 1704. **(D-G)** Representative images of murine cortical brain slices. Slices were stained for mLRP1_DIV* (green) and vascular basement membrane **(D** and **E)**, astrocytic end feet **(F)** and endothelial cells **(G)** (red). Scale bars: 100 μm **(A)** 50 μm – **(B - D)**

Based on the overall promising results in vitro, mLRP1_DIV*’s functional role as transport shuttle should be further explored in the in vivo situation with regard to its relevance for neurodegenerative diseases. To provide an in vivo evidence that LPR1-based receptors can be used as therapeutic strategy, an adeno-associated virus (AAV) that specifically infects only endothelial cells of the BBB was used.

(Fig. 8). Due to the development of this highly specific AAV, only the BBB associated endothelium was efficiently infected after intravenous injection Mice were injected with AAV-(BR1)-mLRP1_DIV* or control at age of approximately 10 weeks and sacrificed 3 months later. mLRP1_DIV*’s expression in the endothelium after intravenous injection was validated by isolation of brain capillaries and endothelial cells from 5xFAD (wt) mice, followed by immunofluorescence and lysis of corresponding tissue and western blot analysis. Expression of HA-tag attached to the C-terminus of mLRP1_DIV* was detected in capillaries of AAV-injected mice (AAV(BR)-mLRP1_DIV*) but not of control mice. As expected, no signal can be found in capillary -depleted brain fractions (Fig. 8B). LRP1 expression is increased in capillary fractions of treated mice due to mLRP1_DIV* delivery, while expression in the brain parenchyma is not altered (Fig. 8C). Relative abundance was compared to actin (loading control). mLRP1_DIV*’s expression in the brain microvasculature was further analyzed using immunofluorescent stainings for co-localization of HA tagged mLRP1_DIV* with the neurovascular unit components collagen IV, astrocytic end feet and endothelial cells. Representative cortical images captured with 100-fold magnification show a widespread detection of mLRP1_DIV* (green) throughout the brain microvasculature (red) (Fig. 8D). Moreover, co-localisation of mLRP1_DIV* with the endothelial marker PECAM1, the vascular basement membrane (collagen IV) and astrocytic end feet (aquaporin 4) confirms mLRP1_DIV* enrichment at the blood brain barrier in murine brain slices (Fig. 8E - G). Together, ex vivo data clearly show mLRP1_DIV*’s expression at the murine BBB after intravenous injection of corresponding AAVs.

## Discussion

Cerebrovascular diseases such as Alzheimer‘s disease (AD), Parkinson, multiple sclerosis and brain cancer are steadily increasing due to medical progress and the associated increase in life expectancy [51]. To date, curing of the most CNS disorders is mostly limited, as the majority of developed drugs are not able to traverse the BBB and enter the brain. A key challenge in drug development is therefore to design not only therapeutics to treat the disease, but rather treatment strategies allowing CNS-active drugs to cross the BBB. In this proof-of-concept study, the potential of the artificial mini LRP1 receptor mLRP1_DIV*, which harbored an N-terminl myc-tag for cargo attachment and lacking the four functional binding domains, for drug delivery across the BBB was investigated. Importantly, using this artificial mLRP1 construct, higher cargo delivery and reduced risk for systemic side effects is ensured compared to targeting endogenous LRP1 due to the exclusive expression at a certain location and its unique binding site. While expressed in vitro, mLRP1_DIV*’s functionality was analyzed based on internalization assays of antibodies and immunoliposomes that target the receptor as well as an apical to basolateral transport of both across an in vitro model of the BBB. Firstly, mLRP1_DIV* was demonstrated to internalize anti-Myc antibodies with high efficiency compared to control (Supplementary Data 1, Figure S3). To examine a potential transport of anti-Myc antibodies mediated by mLRP1_DIV*, hcMEC/D3 cells were cultured into 24-well transparent inserts, forming a defined luminal (blood) and abluminal (brain) compartment. Immunoblotting of proteins present in the abluminal compartment confirmed a successful transcytosis of 9E10 across the monolayer. Transient expression of mLRP1_DIV* resulted in a 2.5-fold higher transport of 9E10 compared to non-transfected cells or to a transport of unspecific antibodies (mIgG), highlighting the use of mLRP1_DIV* as a transport shuttle (Fig. 2). The functional role of the truncated LRP1 receptor was further investigated using immunoliposomes. Liposomes have been utilized extensively as a drug delivery system to increase medication efficacy and reduce drug-related toxicity or undesirable effects and are highly recommended as a carrier for biologically active ingredients. Importantly, the liposomes’ constituents, i.e. phospholipids and cholesterol render them as biodegradable, immunogenicity-free and chemically inactive, with minimal intrinsic toxicity. However, its lipophilic characteristics and large size prevent a simple diffusion across the BBB with the consequent need for further surface functionalization [52–54]. In this context, as active targeting tool to route any liposomal formulation to its corresponding tissue, antibodies represent the most promising way for active targeting. For this reason, anti-Myc antibodies were fused to the surface of immunoliposomes for actively targeting mLRP1_DIV* at the endothelial surface. Regarding internalization assays and immunofluorescence, the data clearly show a specific mLRP1_DIV* mediated internalization of 9E10 functionalized liposomes and the co-localization to mLRP1_DIV* confirmed their attachment to the receptor even after endocytosis (Supplementary Data 1, Figure S6; S7 A -G). Cells lacking the receptor show only a minor internalization and unmodified liposomes did not show an enhanced internalization by mLRP1_DIV* either (Supplementary Data 1, Figure S6). Endocytosis of unmodified liposomes occurred independently from mLRP1_DIV* (Supplementary Data 1, Figure S7 H - J). The investigation of mLRP1_DIV* in the neurovascular context also demonstrated a specific mLRP1_DIV* mediated transport of only 9E10 functionalized and not unmodified liposomes (Fig. 4). Transient expression of mLRP1_DIV* resulted in a 2.3-fold higher transcytosis of 9E10 functionalized liposomes compared to cells lacking the receptor. Competing or inhibiting transcytosis of 9E10 liposomes across hcMEC/D3 mLRP1_DIV* cells by an application of 9E10 antibodies or an incubation temperature of 4 °C resulted in a 50% reduction in transcytosis compared to control (Fig. 4C). Other studies in this field also reported a facilitated drug delivery into the CNS by conjugating single or multiple ligands to liposomal formulations [55, 56]. The active conjugation of antibodies or endogenous molecules can lead not only to a prolonged half-life of liposomes but also to an increased tissue penetration. In this context, dual conjugation of Angiopep-2, exhibiting high LRP1 binding efficiency, and TAT, providing glioma targeting function, to liposomal formulation (DOX-TAT-Ang-LIP) enhanced brain penetration in vitro. Due to its dual conjugation, DOX-TAT-Ang-LIP was not only transcytosed via LRP1 across the BBB, but also entered glioma cells TAT dependently for their subsequent necrosis due to the release of doxorubicin [57]. As the exact trafficking mechanism as well as the fate of mLRP1_DIV* and its ligands after transport were still elusive, the vesicular trafficking route of 9E10, 9E10 - IL and mLRP1_DIV* during transcytosis was investigated based on immunostainings of endothelial cells using the endocytosis marker Clathrin and Caveolin-1, EEA-1 for the early endosome, Lamp-1 for the lysosome, TfR-1 for recycling endosomes, Rab27a as exocytosis marker and p-catenin as basolateral membrane marker (Figs. 3, 5 and 6 Supplementary Data 1, Figure S4-S5; S8-S13). Obtained results show that unspecific mIgG and unmodified liposomes, which do not bind to mLRP1_DIV*, most likely follow a non-specific pinocytosis from the culture medium. This is accompanied by a lack of co-localization with Clathrin and/or Caveolin-1, membrane molecules that are involved in receptor-mediated endocytosis. In addition, mIgG and unmodified liposomes appear to be partially degraded in lysosomes and partially released back into the extracellular space via exocytosis, however independently from mLRP1_DIV* (Supplementary Data 1, Figure S5, S11-S13). Co-localization of 9E10, 9E10 functionalized liposomes and mLRP1_DIV* with Clathrin and Caveolin-1 shows a specific receptor-mediated endocytosis, and their constant co-localization confirms the specific binding of 9E10 and 9E10 liposomes to mLRP1_DIV during the whole transport. In addition, this binding appears to partially circumvent degradation of 9E10 and 9E10 liposomes in lysosomes (Supplementary Data 1, Figure S4; S8-10; S18). Those findings were further supported, as inhibiting lysosomal activity led to no changes in the transcytosis rate of 9E10 across bEnd.3 cells (Supplementary Data 1, Figure S18). Since mLRP1_DIV* and its ligands are also found in recycling endosomes and exocytosing vesicles, but not in early endosomes, a direct transport of 9E10 and 9E10 liposomes, still bound to mLRP1_DIV* from apical to basolateral membrane across the endothelial cell, followed by their mLRP1_DIV* dependent release via Rab27a in the abluminal compartment is suggested. By inhibiting Rab27a during transcytosis of 9E10 or 9E10 immunoliposomes across hcMEC/D3 or bEnd.3 cells, a 60% reduction of transport of both, 9E10 and 9E10 liposomes has been observed. Together, data clearly show a direct transport of cargo from luminal to abluminal side mediated by mLRP1_DIV* and the Rab27a dependent release on the abluminal side (Figs. 3, 5 and 6; Supplementary Data 1, Figure S8-S10).

Similar studies actively explored antibodies that target receptors at the BBB for receptor mediated transcytosis into the brain parenchyma in more detail. Christensen et al., reported monoclonal antibodies (mAbs) targeting basigin located at brain endothelial cells (BECs) to be sorted into recycling vesicles after internalization and thus avoiding lysosomal degradation [45]. Accompanying, using a unique quantitative mass spectrometry technique, studies from Haqqani et al. monitored the endocytic sorting and transcytosis of several internalizing and BBB-crossing antibody forms in BECs. Thereby, FC5, which interacts with a glycosylated epitope on the luminal side of BEC, was internalized into BEC and dispersed 70%:30% between early and late endosomes, resulting in an efficient release on the BEC monolayer’s abluminal side [58]. However, in present transport studies, only a specific receptor-mediated endocytosis of cargo and their presence in the brain parenchyma in vitro has been reported so far. The vesicular trafficking route of transported cargo and its release at the basolateral surface could not be demonstrated. Notably, our proof-of-concept study clearly highlights mLRP1_DIV*’s role as luminal to basolateral transport carrier of antibodies and liposomes into the CNS and suggest mLRP1_DIV* as promising trojan horse for drug delivery across the BBB. In the past decades, some NSAIDs have been considered for the treatment of AD, due to their ability to modulate γ-secretase activity and Aβ generation, importantly, without interfering with other APP processing pathways or Notch signaling [59]. However, NSAIDs are hampered by low brain penetration and have failed in clinical trials [60–62]. Therefore, mechanisms facilitating drug delivery into the brain, including liposome-based systems have been intensely investigated in the past years [55]. Here, to further explore our concept for improving delivery of therapeutics into the CNS, the capability of mLRP1_DIV* to ferry 9E10 functionalized immunoliposomes loaded with the GSM BB25 across the BBB was investigated. Previous studies had reported that BB25 modulated γ-secretase activity with much higher potency compared to NSAID GSMs such as ibuprofen or flurbiprofen [39]. Consequently, the biological activity of liposomal BB25 was compared to free BB25 after an application to PS70 cells in an initial experiment (Supplementary Data 1, Figure S14). Liposomal BB25 modulated γ-secretase activity in the same way as free BB25 resulting in a 90% reduction of Aß_42_ levels and a concomitant 1.4-fold increase of Aß_38_ levels. An application of unloaded liposomes displayed no γ-secretase modulating effect as expected due to the missing embedded GSM (Supplementary Data 1, Figure S14). To show that BB25 loaded 9E10 functionalized liposomes are still intact and more importantly still functional even after a transport across an endothelial monolayer, a co-culture model using bEnd.3 mLRP1_DIV* cells combined with abluminally cultured PS70 cells was established. In this experimental setup, free BB25 did not alter Aß_38_ or Aß_42_ levels compared to control, indicating an insufficient diffusion even at high concentrations across the bEnd.3 mLRP1_DIV* monolayer (Fig. 7B and C). In our proof-of-concept study, liposomal BB25 displayed the typical characteristics of a GSM after mLRP1_DIV* dependent transport across bEnd.3 cells, with a decrease of Aß_42_ and an increase of Aß_38_ levels. Such an effect could not be demonstrated after application of unloaded 9E10 functionalized liposomes, which were transported with the same efficiency as BB25 loaded 9E10 liposomes (Fig. 7B and C; Table 2). Importantly, the modulation of γ-secretase activity could only be demonstrated after the application of BB25–9E10 - IL to bEnd.3 cells expressing mLRP1_DIV*, whereas the application of BB25–9E10 - IL to empty vector-transfected bEnd.3 cells did not show such an effect (Supplementary Data 1, Figure S16). Consequently, the in vitro co-culture studies clearly demonstrated an mLRP1_DIV* dependent transport of BB25 loaded 9E10 functionalized liposomes, as well as their abluminal release into the brain parenchyma, where they exhibited their indented effect. Importantly, the modulation of γ-secretase activity can be clearly attributed to the mLRP1_DIV* mediated transport of liposomal BB25 and not to differences in the integrity of the BBB between the experimental groups, as TEER and paracellular diffusion of FITC-dextran averaged out at the same level in all groups (Fig. 7D and E). Moreover, liposomal BB25, unloaded liposomes as well as free BB25 did not show any adverse effect on the BBB in vitro, as no changes in endothelial barrier properties could be detected after their application compared to the control condition (Supplementary Data 1, Figure S15). As mentioned above, conjugation of ligands to liposomal formulations facilitate their binding to receptors at the BBB and subsequent transcytosis [55]. In this context, Osthole, a coumarin derivate believed to neutralize Aß induced neurotoxicity through neuroprotective effects, was encapsulated into transferrin functionalized liposomes (Tf-Ost-Lip) [63]. Normally, the drug’s solubility, bioavailability and low BBB permeability restrict its effectiveness. However, corresponding in vitro studies confirm an increase of Osthole into hcMEC/D3 cells followed by an enhanced drug concentration at the BBB, when loaded into Tf-liposomes. Additionally, Tf-Ost-Lip enhanced the accumulation of Ost in the brain and lengthened the cycle time in mice, according to in vivo investigations on pharmacokinetics and the distribution of Ost in brain tissue. Moreover, Tf-Ost-Lip was shown to improve Ost’s ability to reduce pathology associated with Alzheimer’s disease. Based on the overall promising results in vitro, mLRP1_DIV*’s functional role as transport shuttle was further explored in the in vivo situation regarding its relevance for neurodegenerative diseases. To provide an in vivo evidence that LPR1-based receptors can be used as therapeutic strategy, an adeno-associated virus (AAV) that specifically infects only endothelial cells of the BBB was used (Fig. 8). Due to the development of this highly specific AAV, only the BBB associated endothelium was efficiently infected after intravenous injection with AAV-(BR1)-mLRP1_DIV*. In general, mLRP1_DIV* mediated transport of BB25 loaded liposomes as drug delivery mechanism offers decisive advantages compared to current drug delivery strategies as well as to existing AD therapies including Aduhelm, Lecanemab or Donanemab. All are human or humanized IgG monoclonal antibodies against Aß aimed to neutralize Aß levels in the brain and have been shown to slow cognitive and functional decline in early AD [64, 65]. However, clinical trials demonstrated that current amyloid immunotherapies poorly enter the brain parenchyma and linger in the choroid plexus and ventricles five days after infusion. One hypothesis suggests the entry of amyloid immunotherapies at the blood-CSF-barrier (BCSFB) with the consequent accumulation more likely in the CSF rather than the entire brain parenchyma, which explains the moderate effects in clinical trails and their questionable use in the future. Additionally, during several recent Aβ immunotherapy trials, the most frequent and severe adverse event resulting from pathological alterations in the cerebral vasculature was shown to be amyloid-related imaging abnormalities (ARIA), either in form of cerebral edema or microhemorrhages. Currently unknown are the exact physiological and molecular processes via which amyloid immunotherapy amplifies the changes in vascular permeability and microhemorrhages caused by cerebral amyloid angiopathy (CAA) [66]. Most data suggests that due to the entry at the BCSFB anti-Aß antibodies directly interfere with vascular Aß leading to pathological alteration in the vasculature. For this reason, new strategies have already been developed to ensure a drug delivery across the BBB rather than across the BCSFB resulting in a widespread distribution of therapeutic agents across the entire brain parenchyma. In this context, Roche scientist explored alternate delivery methods allowing lower drug dosing with still higher potency. By fusing the anti-amyloid monoclonal antibody gantenerumab to Fc fragments of the human transferrin receptor (TfR-1), extensively expressed at the luminal side of the BBB, researchers achieve an effective CNS delivery and distribution as well as little ARIAs compared to conventional antibody therapies [19]. Although this shuttle system offers an efficient transport of corresponding ligands into the CNS and prevents severe side effects related to pathological vascular alteration, its use may induce other dysfunctional physiological effects. On the one hand, TfR-1 targeting therapeutics compete with endogenous ligands, which may perturb the normal biological functions of the receptor and on the other hand TfR-1 is systemically expressed, thus TfR-1 targeting drugs may induce adverse side effects in other organs and tissues. For this reason, the generation of mLRP1_DIV*, an artificial receptor based on native LRP1 represents an alternative targeting system, which could prevent potential systemic side effects due to its unique binding site and its exclusive expression in the brain endothelium using AAV based gene therapy. Signal transduction is therefore only triggered by corresponding intended exogenous ligands and no natural endogenous ligands compete with the binding to mLRP1_DIV*. Moreover, mLRP1_DIV* targeting CNS-active drugs are only delivered into the CNS and sidesteps other organs and tissues due to mLRP1_DIV*’s specific expression at the BBB. Nevertheless, further in vivo studies regarding the immunogenicity of mLRP1_DIV*, safety of overexpressing an artificial receptor at the BBB and the effect on the expression of other BBB receptors should be conducted. Although, we already showed that an expression of mLRP1_DIV* in endothelial cells had no effect on the protein level of native LRP1 in vitro, the expression of several LDL receptor family proteins should be investigated in the in vivo situation (Supplementary Date 1; Figure S19). Since mLRP1_DIV* is an artificial version of LRP1, with a strongly truncated DIV of LRP1, we assume a high level of safety upon expression. No ligands other than anti-Myc antibodies that normally bind to LRP1, e.g. Aß or tPa have been shown to bind to the receptor (Data not shown). Despite our promising in vitro data of mLRP1_DIV*-mediated delivery of drug-loaded immunoliposomes across an in vitro BBB and its brain endothelial expression in vivo using AAV-based gene therapy, the immunogenicity of this AAV-based gene therapy in vivo should be investigated in the future. Studies have already shown that immunogenicity significantly complicates the safety and efficacy of gene therapies based on AAV vectors and represents an increasing challenge in gene therapy. AAV gene therapies have been associated with mild to severe adverse side effects during clinical development, which has raised strong doubts about the use of such gene therapies. Thereby, humoral and cellular immune response against the viral capsid as well as the transgene protein product remains a serious challenge. The complexity of the immunogenicity of AAV gene therapies arises from the multitude of risk factors associated with their components and the pre-existing immunity of the subjects [67–73]. However, it is not only the immunogenicity of AAV gene therapy that remains a challenge for our new drug delivery mechanism. It is also the use of lipids based nanoparticles (LNPs), especially with regard to pharmacodynamics and pharmacokinetics of water insoluble, poor bioavailable and highly toxic drugs, that should be addressed [74]. The therapeutic potential of nanoparticles as cutting-edge drug delivery technologies that enhance traditional pharmacology has gained widespread recognition throughout the last ten years. LNPs have garnered significant attention in preclinical and clinical research due to their exceptional pharmacological performance and potential therapeutic benefits, among other nanomaterials [75, 76]. Especially, liposomes are superior to typical drug delivery systems, as they allow site-targeting, controlled or prolonged release, protection against drug degradation and clearance, better therapeutic effects, and less harmful side effects due to their biocompatibility, biodegradability, and low immunogenicity. Over the past few decades, a number of liposomal drug products have been approved and successfully used in clinics due to these benefits [29, 76]. Additionally, liposomes can be administered via a variety of routes, such as parenteral, transdermal, pulmonary, ocular, and oral, for both diagnostic and therapeutic purposes [77–80]. However, the chemical and physical stability of liposomes presents significant hurdles. Consequently, the development of liposomes with high stability is crucial since it greatly influences their therapeutic applicability, pharmacodynamics and pharmacokinetics [76, 81]. Despite the many challenges posed by our drug delivery mechanism, including the immunogenicity of AAV-based gene therapies and the hurdles of liposome stability and manufacturing and the resulting dynamics and kinetics of administered drugs, our proof-of-concept study represents a critical first step in the development of new minimally invasive and safe drug delivery strategies. Future in vivo experiments in mice will clarify whether our in vitro drug delivery mechanism can also be transferred to the in vivo situation, in particular with focus on the immunogenicity and safety of AAV gene therapy as well as the efficiency and safety of drug loaded immunoliposomes. Regarding clinical studies on patients, the greatest challenge is already apparent, namely the development of an AAV that specifically infects the human endothelium of the BBB for treatment of CNS disorders.

## Conclusion

Our proof-of-concept study verified for the first time the artificial LRP1 mini receptor (mLRP1_DIV*) as auspicious carrier of any cargo into the CNS across an in vitro model of the BBB. It not only provides an endocytosis of cargo into an endothelial cell, but also allows a straight transport of cargo from luminal to abluminal side across an endothelial monolayer and it’s release into brain parenchyma in vitro, where it exhibits its intended therapeutic effect. Further in vivo experiments are needed to clarify the functionality of mLRP1_DIV* mediated delivery of drug loaded liposomes in the physiological context and in clinical applications.

## Electronic supplementary material

Below is the link to the electronic supplementary material.

Supplementary Material 1

Supplementary Material 2

Supplementary Material 3

Supplementary Material 4

Supplementary Material 5

Supplementary Material 6

Supplementary Material 7

Supplementary Material 8

Supplementary Material 9

## Data Availability

All data generated or analyzed during this study are included in this published article and its supplementary information files.

## Abbreviations

AAV: Aadeno-associated virus
AD: Alzheimer’s disease
Aß: Amyloid ß
APP: Amyloid precursor protein
ARIA: Amyloid related imaging abnormalities
9E10: Anti-Myc antibodies
9E10 – IL: Anti-Myc antibodies functionalized immunoliposomes
BACE1: β-site amyloid precursor protein cleaving enzyme
BBB: Blood-brain-barrier
BCSFB: Blood CSF barrier
BB25–9E10 – IL: BB25 loaded anti-Myc antibodies functionalized immunoliposomes
BSA: Bovine serum albumin
BCA: Bicinchoninic acid
BCEC: Brain capillary endothelial cell
bEnd.3: Brain derived endothelial cells
CAA: Cerebral amyloid angiopathy
CCI: Capacitance
CNS: Central nervous system
CHO: Chinese hamster ovary
DAPI: 4′,6-diamidino-2-phenylindole
DMEM: Dulbecco’s modified eagle medium
DMSO: Dimethyl sulfoxide
DPBS: Dulbecco’s phosphate-buffered saline
DSPE-PEG-2000: 1,2-Distearoyl-sn-glycero-3phosphoethanolamin-N-[amino(polyethylenglycol)-2000
EDTA: Ethylenediaminetetraacetic acid
ELISA: Enzyme-linked immunosorbent assay
EPC: 1,2-dioleoyl-sn-glycero-3ethylphosphocholine
FITC: Fluorescein isothiocyanate
GSM: Gamma secretase modulator
HA: Hemagglutinin ()
hcMEC/D3: Human brain microvascular endothelial cells
HRP: Horseradish peroxidase
JAMs: Junctional adhesion molecules
IgG: Immunoglobulin G
LNPs: Lipid based nanoparticles
LRP1: Low-density lipoprotein receptor-related protein 1
NP: Nanoparticle
NaN_3_: Natrium azide
NaCl: Natrium chloride
NP40: Nonidet P 40
NSAID: Nonsteroidal anti-inflammatory drug
PSEN 1: Presenilin 1
PS70: Chinese hamster ovary cells overexpressing amyloid precursor protein and presenilin 1
RMT: Receptor-mediated transcytosis
RT: Room temperature
PAGE: Polyacrylamide gel electrophoresis
PFA: Paraformaldehyde
SEM: Standard error of the mean
SATA: S-succinimidyl-S-thioacetate
SDS: Sodium dodecyl sulfate
SLC: Solute carrier
H_2_SO_4_: Sulfuric acid
TCA: Trichloroacetic acid
TEER: Transendothelial electrical resistance
TJ: Tight junction
TMB: 3,3’,5,5’-tetramethylbenzidin
unm. – IL: Unmodified immunoliposomes
WLL: White light laser
Wt: Wild type
