# Supplementary figures and images for: Truncated mini LRP1 transports cargo from luminal to basolateral side across the blood brain barrier
Source: Fluids Barriers CNS. 2024 Sep 17;21:74. doi: 10.1186/s12987-024-00573-1 (PMC11409491; doi:10.1186/s12987-024-00573-1)

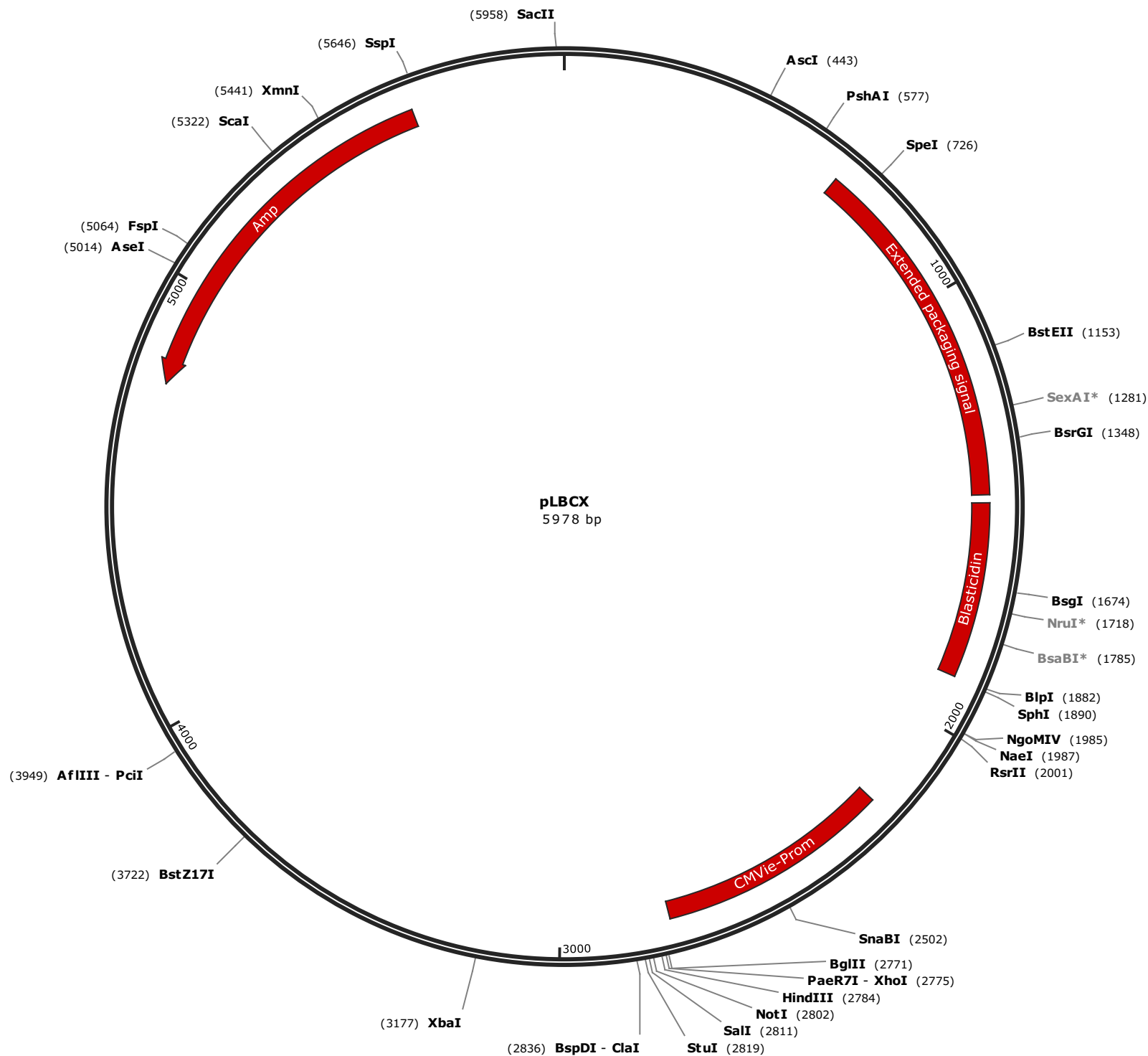

Supplement: Supplementary file 1 — Supplementary Material 1 [file 12987_2024_573_MOESM1_ESM.pdf]

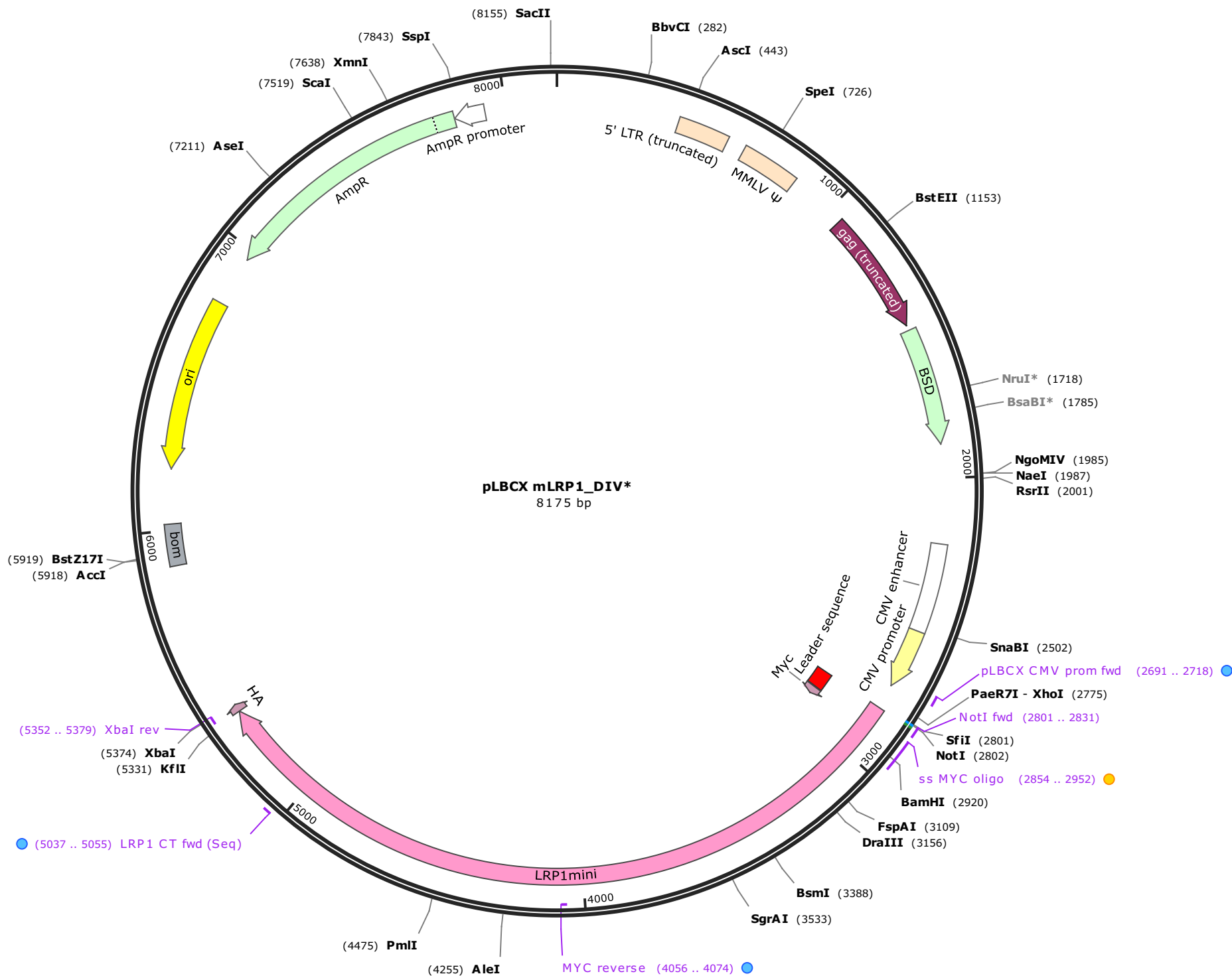

Supplement: Supplementary file 2 — Supplementary Material 2 [file 12987_2024_573_MOESM2_ESM.pdf]

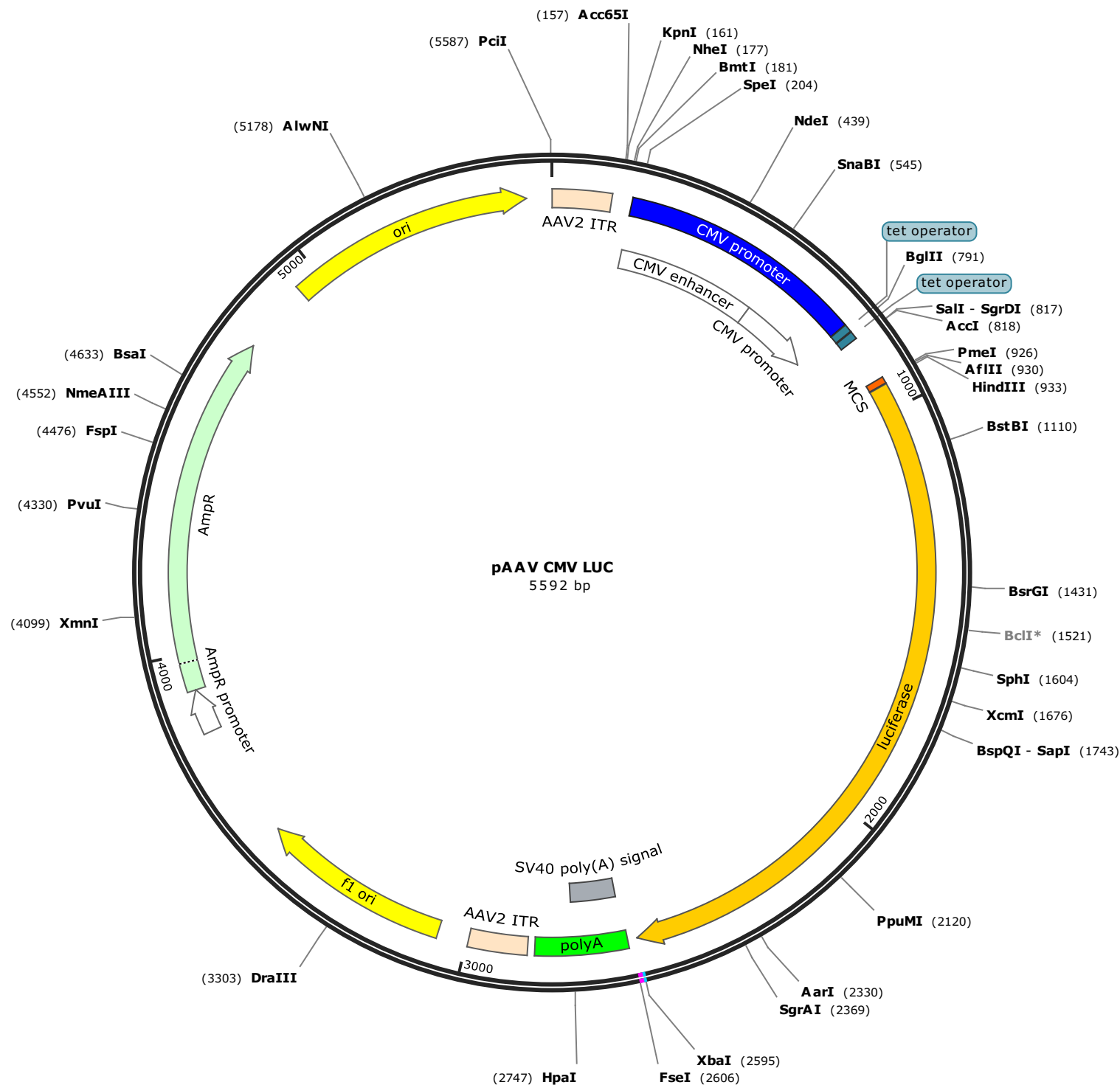

Supplement: Supplementary file 3 — Supplementary Material 3 [file 12987_2024_573_MOESM3_ESM.pdf]

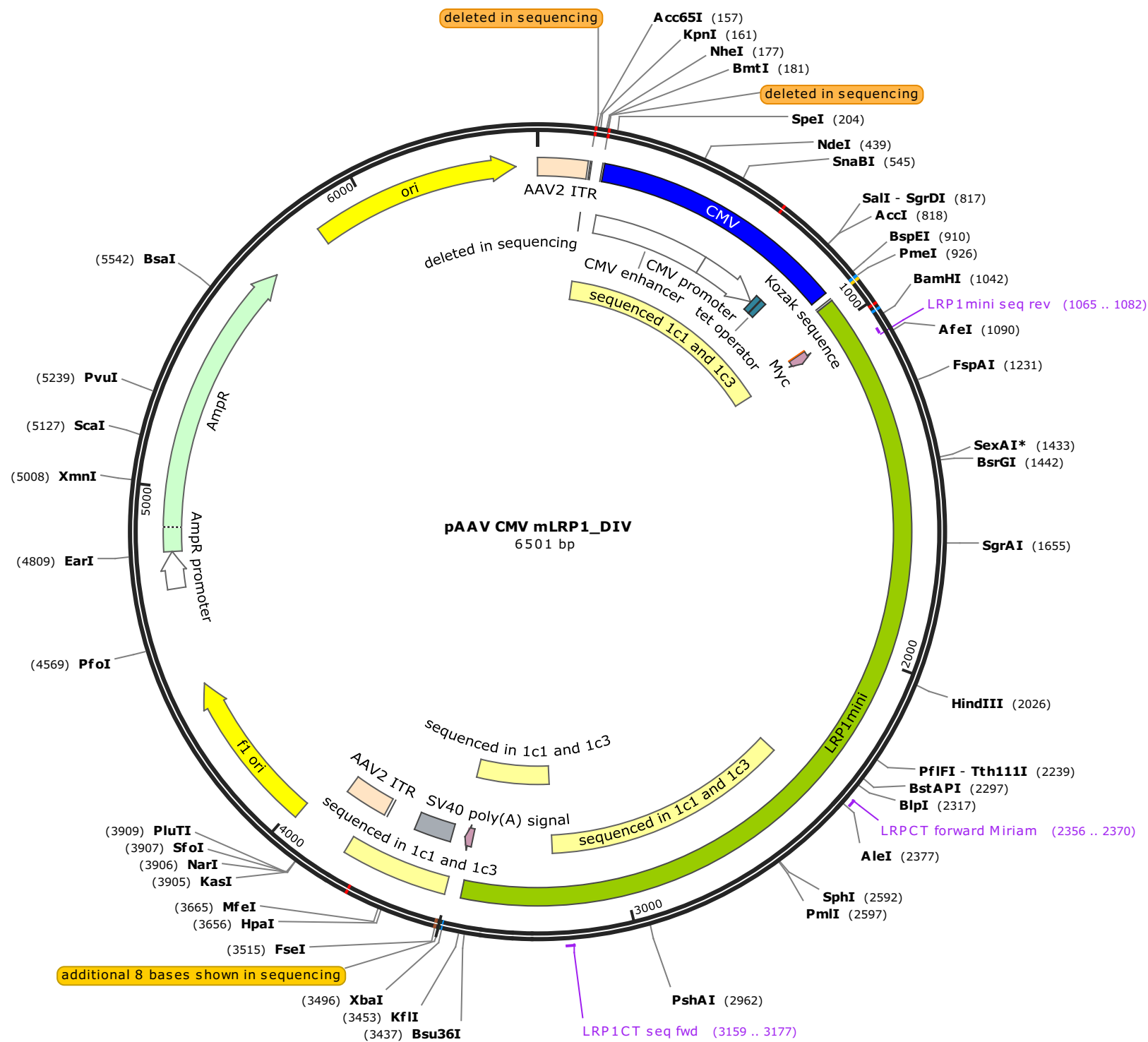

Supplement: Supplementary file 4 — Supplementary Material 4 [file 12987_2024_573_MOESM4_ESM.pdf]

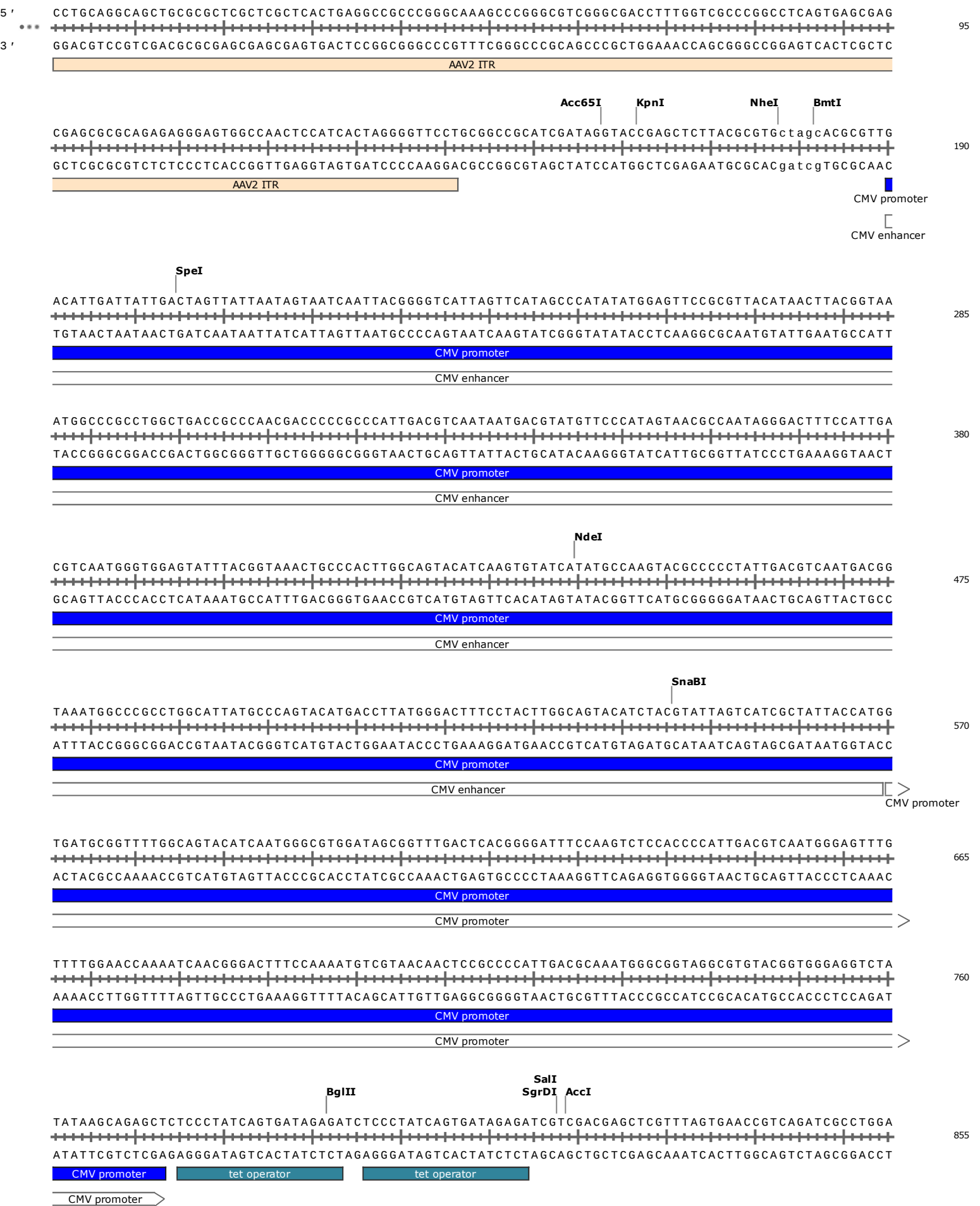

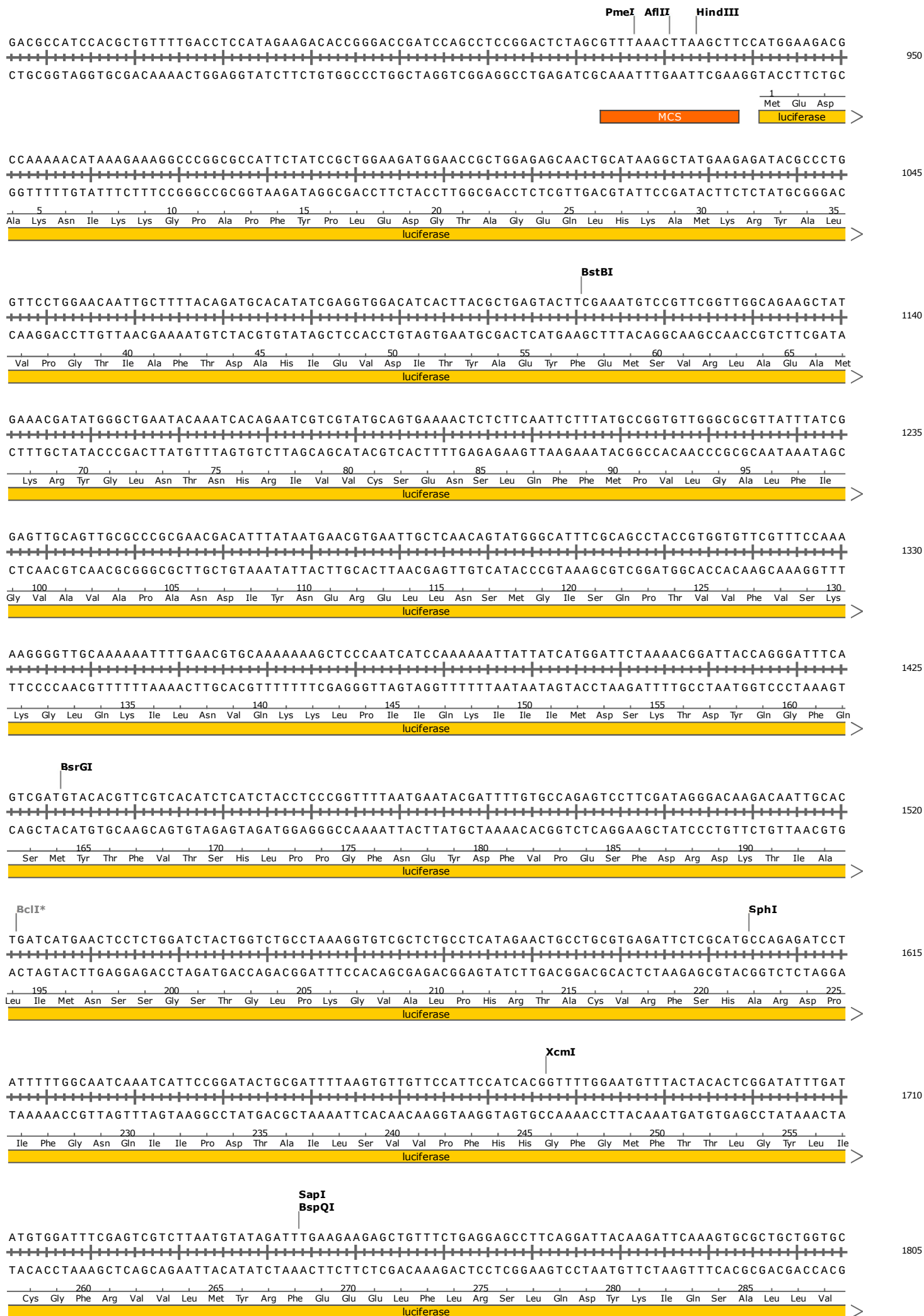

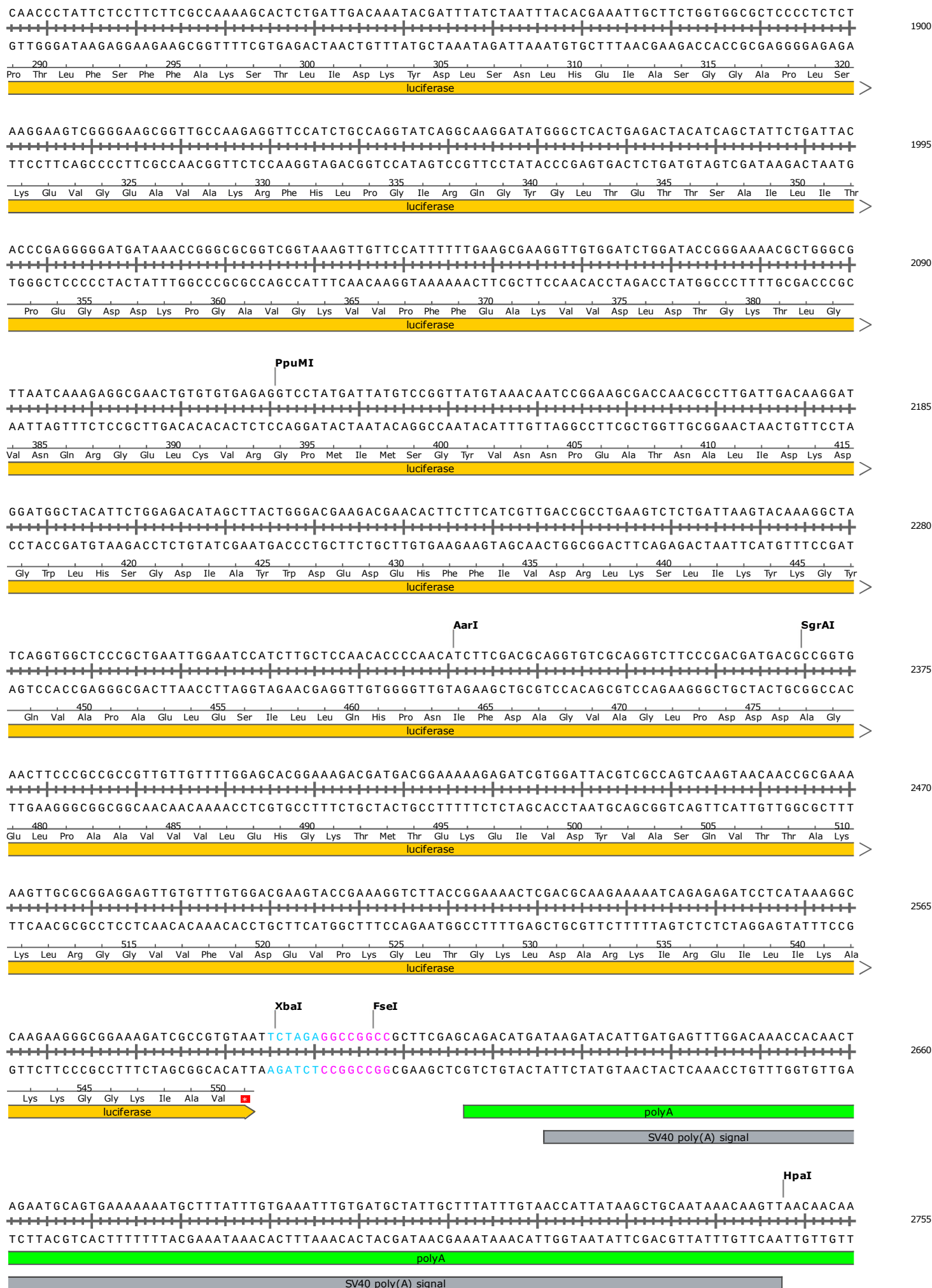

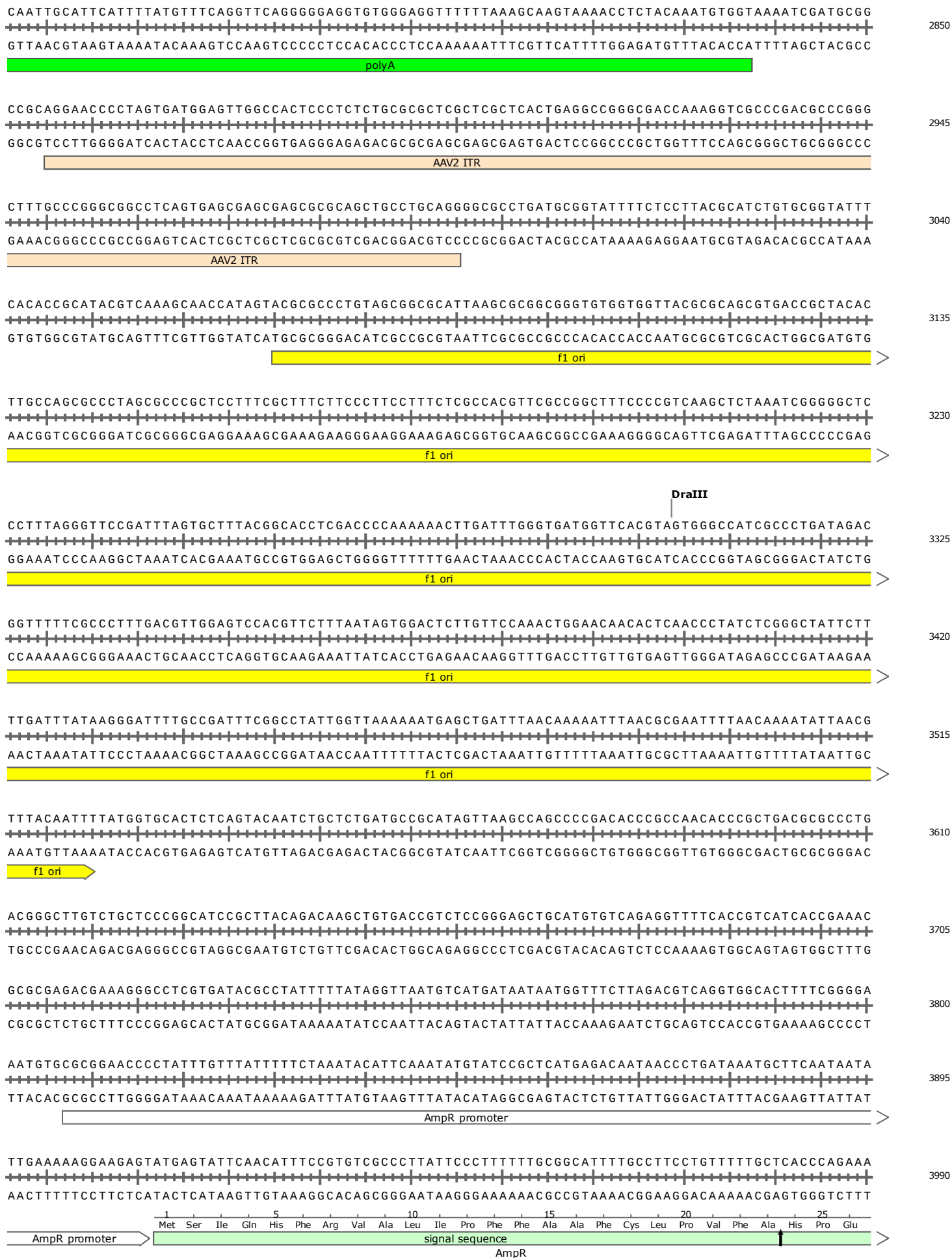

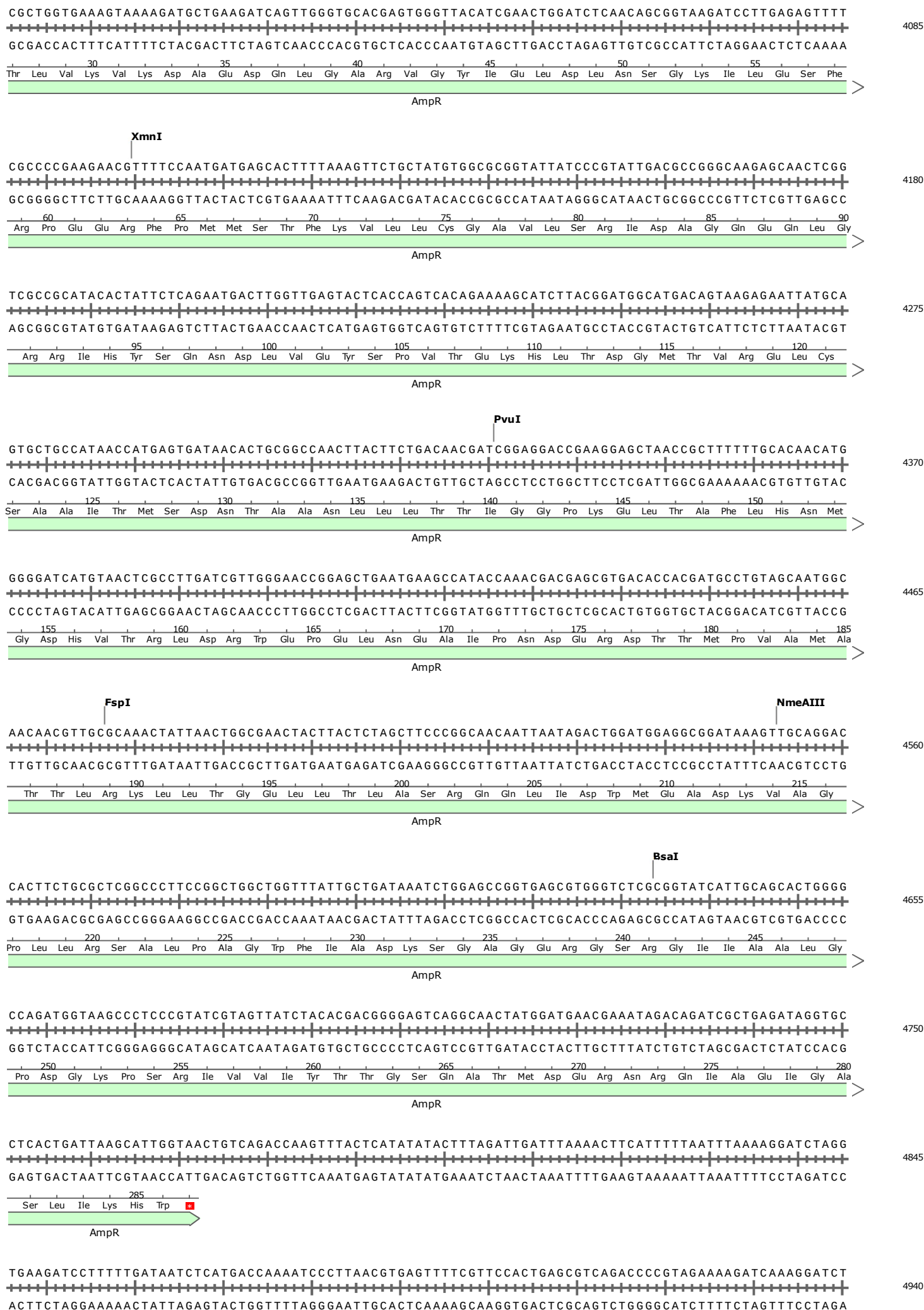

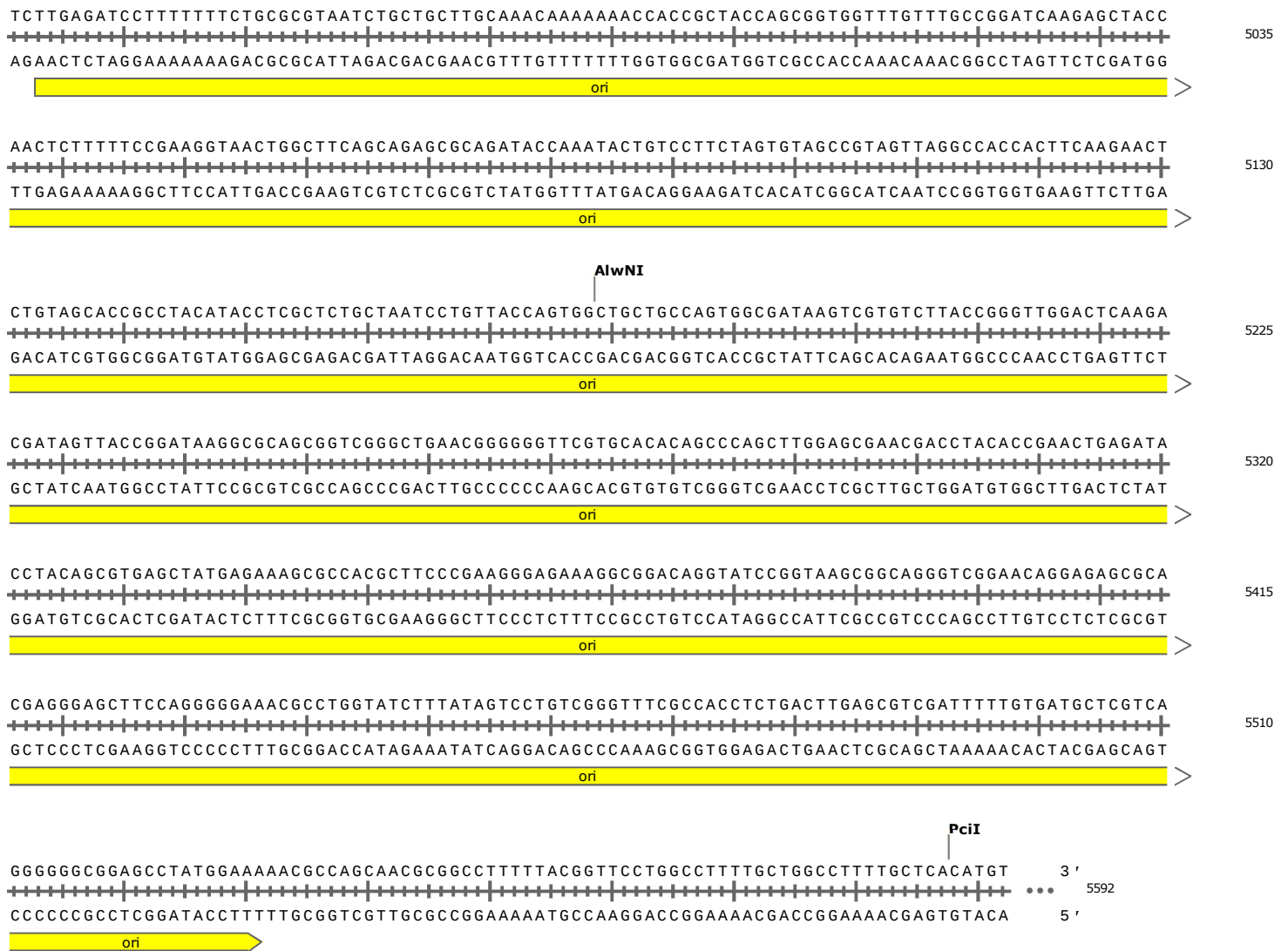

Supplement: Supplementary file 6 — Supplementary Material 6 [file 12987_2024_573_MOESM6_ESM.pdf]
