# Supplementary material for: Truncated mini LRP1 transports cargo from luminal to basolateral side across the blood brain barrier
Source: Fluids Barriers CNS. 2024 Sep 17;21:74. doi: 10.1186/s12987-024-00573-1 (PMC11409491; doi:10.1186/s12987-024-00573-1)
Supplement: Supplementary file 5 — Supplementary Material 5 [file 12987_2024_573_MOESM5_ESM.pdf]

```
5' TTTGAAAGACCCCACCCGTAGGTGGCAAGCTAGCTTAAGTAACGCCACTTTGCAAGGCATGGAAAAATACATAACTGAGAATAGAAAAGT   90
3' AAACTTTCTGGGGTGGGCATCCACCGTTCGATCGAATTCATTGCGGTGAAACGTTCCGTACCTTTTTATGTATTGACTCTTATCTTTTCA

TCAGATCAAGGTCAGGAACAAAGAAACAGCTGAATACCAAACAGGATATCTGTGGTAAGCGGTTCCTGCCCCGGCTCAGGGCCAAGAACA   180
AGTCTAGTTCCAGTCCTTGTTTCTTTGTCGACTTATGGTTTGTCCTATAGACACCATTCGCCAAGGACGGGGCCGAGTCCCGGTTCTTGT

GATGAGACAGCTGAGTGATGGGCCAAACAGGATATCTGTGGTAAGCAGTTCCTGCCCCGGCTCGGGGCCAAGAACAGATGGTCCCCAGAT   270
CTACTCTGTCGACTCACTACCCGGTTTGTCCTATAGACACCATTCGTCAAGGACGGGGCCGAGCCCCGGTTCTTGTCTACCAGGGGTCTA

GCGGTCCAGCCCTCAGCAGTTTCTAGTGAATCATCAGATGTTTCCAGGGTGCCCCAAGGACCTGAAAATGACCCTGTACCTTATTTGAAC   360
CGCCAGGTCGGGAGTCGTCAAAGATCACTTAGTAGTCTACAAAGGTCCCACGGGGTTCCTGGACTTTTACTGGGACATGGAATAAACTTG

AscI
TAACCAATCAGTTCGCTTCTCGCTTCTGTTCGCGCGCTTCCGCTCTCCGAGCTCAATAAAAGAGCCCACAACCCCTCACTCGGCGCGCCA   450
ATTGGTTAGTCAAGCGAAGAGCGAAGACAAGCGCGCGAAGGCGAGAGGCTCGAGTTATTTTCTCGGGTGTTGGGGAGTGAGCCGCGCGGT

GTCTTCCGATAGACTGCGTCGCCCGGGTACCCGTATTCCCAATAAAGCCTCTTGCTGTTTGCATCCGAATCGTGGTCTCGCTGTTCCTTG   540
CAGAAGGCTATCTGACGCAGCGGGCCCATGGGCATAAGGGTTATTTCGGAGAACGACAAACGTAGGCTTAGCACCAGAGCGACAAGGAAC

PshAI
GGAGGGTCTCCTCTGAGTGATTGACTACCCACGACGGGGGTCTTTCATTTGGGGGCTCGTCCGGGATTTGGAGACCCCTGCCCAGGGACC   630
CCTCCCAGAGGAGACTCACTAACTGATGGGTGCTGCCCCCAGAAAGTAAACCCCCGAGCAGGCCCTAAACCTCTGGGGACGGGTCCCTGG

ACCGACCCACCACCGGGAGGTAAGCTGGCCAGCAACTTATCTGTGTCTGTCCGATTGTCTAGTGTCTATGTTTGATGTTATGCGCCTGCG   720
TGGCTGGGTGGTGGCCCTCCATTCGACCGGTCGTTGAATAGACACAGACAGGCTAACAGATCACAGATACAAACTACAATACGCGGACGC
Extended packaging signal

SpeI
TCTGTACTAGTTAGCTAACTAGCTCTGTATCTGGCGGACCCGTGGTGGAACTGACGAGTTCTGAACACCCGGCCGCAACCCTGGGAGACG   810
AGACATGATCAATCGATTGATCGAGACATAGACCGCCTGGGCACCACCTTGACTGCTCAAGACTTGTGGGCCGGCGTTGGGACCCTCTGC
Extended packaging signal

TCCCAGGGACTTTGGGGGCCGTTTTTGTGGCCCGACCTGAGGAAGGGAGTCGATGTGGAATCCGACCCCGTCAGGATATGTGGTTCTGGT   900
AGGGTCCCTGAAACCCCCGGCAAAAACACCGGGCTGGACTCCTTCCCTCAGCTACACCTTAGGCTGGGGCAGTCCTATACACCAAGACCA
Extended packaging signal

AGGAGACGAGAACCTAAAACAGTTCCCGCCTCCGTCTGAATTTTTGCTTTCGGTTTGGAACCGAAGCCGCGCGTCTTGTCTGCTGCAGCG   990
TCCTCTGCTCTTGGATTTTGTCAAGGGCGGAGGCAGACTTAAAAACGAAAGCCAAACCTTGGCTTCGGCGCGCAGAACAGACGACGTCGC
Extended packaging signal

CTGCAGCATCGTTCTGTGTTGTCTCTGTCTGACTGTGTTTCTGTATTTGTCTGAAAATTAGGGCCAGACTGTTACCACTCCCTTAAGTTT   1080
GACGTCGTAGCAAGACACAACAGAGACAGACTGACACAAAGACATAAACAGACTTTTAATCCCGGTCTGACAATGGTGAGGGAATTCAAA
Extended packaging signal

BstEII
GACCTTAGGTCACTGGAAAGATGTCGAGCGGATCGCTCACAACCAGTCGGTAGATGTCAAGAAGAGACGTTGGGTTACCTTCTGCTCTGC   1170
CTGGAATCCAGTGACCTTTCTACAGCTCGCCTAGCGAGTGTTGGTCAGCCATCTACAGTTCTTCTCTGCAACCCAATGGAAGACGAGACG
Extended packaging signal

AGAATGGCCAACCTTTAACGTCGGATGGCCGCGAGACGGCACCTTTAACCGAGACCTCATCACCCAGGTTAAGATCAAGGTCTTTTCACC   1260
TCTTACCGGTTGGAAATTGCAGCCTACCGGCGCTCTGCCGTGGAAATTGGCTCTGGAGTAGTGGGTCCAATTCTAGTTCCAGAAAAGTGG
Extended packaging signal
```

SexAI* BsrGI
TGGCCCGCATGGACACCCAGACCAGGTCCCCTACATCGTGACCTGGGAAGCCTTGGCTTTTGACCCCCCTCCCTGGGTCAAGCCCTTTGT 1350
ACCGGGCGTACCTGTGGGTCTGGTCCAGGGGATGTAGCACTGGACCCTTCGGAACCGAAAACTGGGGGGAGGGACCCAGTTCGGGAAACA
Extended packaging signal

ACACCCTAAGCCTCCGCCTCCTCTTCCTCCATCCGCCCCGTCTCTCCCCCTTGAACCTCCTCGTTCGACCCCGCCTCGATCCTCCCTTTA 1440
TGTGGGATTCGGAGGCGGAGGAGAAGGAGGTAGGCGGGGCAGAGAGGGGGAACTTGGAGGAGCAAGCTGGGGCGGAGCTAGGAGGGAAAT
Extended packaging signal

TCCAGCCCTCACTCCTTCTCTAGGCGCCGGAATTCCGATCTGATCCATGGCCAAGCCTTTGTCTCAAGAAGAATCCACCCTCATTGAAAG 1530
AGGTCGGGAGTGAGGAAGAGATCCGCGGCCTTAAGGCTAGACTAGGTACCGGTTCGGAAACAGAGTTCTTCTTAGGTGGGAGTAACTTTC
Extended packaging signal Blasticidin

AGCAACGGCTACAATCAACAGCATCCCCATCTCTGAAGACTACAGCGTCGCCAGCGCAGCTCTCTCTAGCGACGGCCGCATCTTCACTGG 1620
TCGTTGCCGATGTTAGTTGTCGTAGGGGTAGAGACTTCTGATGTCGCAGCGGTCGCGTCGAGAGAGATCGCTGCCGGCGTAGAAGTGACC
Blasticidin

BsgI
TGTCAATGTATATCATTTTACTGGGGGACCTTGTGCAGAACTCGTGGTGCTGGGCACTGCTGCTGCTGCGGCAGCTGGCAACCTGACTTG 1710
ACAGTTACATATAGTAAAATGACCCCCTGGAACACGTCTTGAGCACCACGACCCGTGACGACGACGACGCCGTCGACCGTTGGACTGAAC
Blasticidin

NruI* BsaBI*
TATCGTCGCGATCGGAAATGAGAACAGGGGCATCTTGAGCCCCTGCGGACGGTGCCGACAGGTGCTTCTCGATCTGCATCCTGGGATCAA 1800
ATAGCAGCGCTAGCCTTTACTCTTGTCCCCGTAGAACTCGGGGACGCCTGCCACGGCTGTCCACGAAGAGCTAGACGTAGGACCCTAGTT
Blasticidin

BlpI SphI
AGCCATAGTGAAGGACAGTGATGGACAGCCGACGGCAGTTGGGATTCGTGAATTGCTGCCCTCTGGTTATGTGTGGGAGGGCTAAGCATG 1890
TCGGTATCACTTCCTGTCACTACCTGTCGGCTGCCGTCAACCCTAAGCACTTAACGACGGGAGACCAATACACACCCTCCCGATTCGTAC
Blasticidin

CCCGACGGCGAGGATCTCGTCGTGACCCATGGCGATGCCTGCTTGCCGAATATCATGGTGGAAAATGGCCGCTTTTCTGGATTCATCGAC 1980
GGGCTGCCGCTCCTAGAGCAGCACTGGGTACCGCTACGGACGAACGGCTTATAGTACCACCTTTTACCGGCGAAAAGACCTAAGTAGCTG

NgoMIV NaeI RsrII
TGTGGCCGGCTGGGTGTGGCGGACCGCTATCAGGACATAGCGTTGGCTACCCGTGATATTGCTGAAGAGCTTGGCGGCGAATGGGCTGAC 2070
ACACCGGCCGACCCACACCGCCTGGCGATAGTCCTGTATCGCAACCGATGGGCACTATAACGACTTCTCGAACCGCCGCTTACCCGACTG

CGCTTCCTCGTGCTTTACGGTATCGCCGCTCCCGATTCGCAGCGCATCGCCTTCTATCGCCTTCTTGACGAGTTCTTCTGAGCGGGACTC 2160
GCGAAGGAGCACGAAATGCCATAGCGGCGAGGGCTAAGCGTCGCGTAGCGGAAGATAGCGGAAGAACTGCTCAAGAAGACTCGCCCTGAG

TGGGGTTCGTAATAGTAATCAATTACGGGGTCATTAGTTCATAGCCCATATATGGAGTTCCGCGTTACATAACTTACGGTAAATGGCCCG 2250
ACCCCAAGCATTATCATTAGTTAATGCCCCAGTAATCAAGTATCGGGTATATACCTCAAGGCGCAATGTATTGAATGCCATTTACCGGGC
CMVie-Prom

CCTGGCTGACCGCCCAACGACCCCCGCCCATTGACGTCAATAATGACGTATGTTCCCATAGTAACGCCAATAGGGACTTTCCATTGACGT 2340
GGACCGACTGGCGGGTTGCTGGGGGCGGGTAACTGCAGTTATTACTGCATACAAGGGTATCATTGCGGTTATCCCTGAAAGGTAACTGCA
CMVie-Prom

CAATGGGTGGAGTATTTACGGTAAACTGCCCACTTGGCAGTACATCAAGTGTATCATATGCCAAGTACGCCCCCTATTGACGTCAATGAC 2430
GTTACCCACCTCATAAATGCCATTTGACGGGTGAACCGTCATGTAGTTCACATAGTATACGGTTCATGCGGGGGATAACTGCAGTTACTG
CMVie-Prom

SnaBI
GGTAAATGGCCCGCCTGGCATTATGCCCAGTACATGACCTTATGGGACTTTCCTACTTGGCAGTACATCTACGTATTAGTCATCGCTATT 2520
CCATTTACCGGGCGGACCGTAATACGGGTCATGTACTGGAATACCCTGAAAGGATGAACCGTCATGTAGATGCATAATCAGTAGCGATAA
CMVie-Prom

ACCATGGTGATGCGGTTTTGGCAGTACATCAATGGGCGTGGATAGCGGTTTGACTCACGGGGATTTCCAAGTCTCCACCCCATTGACGTC 2610
TGGTACCACTACGCCAAAACCGTCATGTAGTTACCCGCACCTATCGCCAAACTGAGTGCCCCTAAAGGTTCAGAGGTGGGGTAACTGCAG
CMVie-Prom

AATGGGAGTTTGTTTTGGCACCAAAATCAACGGGACTTTCCAAAATGTCGTAACAACTCCGCCCCATTGACGCAAATGGGCGGTAGGCGT 2700
TTACCCTCAAACAAAACCGTGGTTTTAGTTGCCCTGAAAGGTTTTACAGCATTGTTGAGGCGGGGTAACTGCGTTTACCCGCCATCCGCA
CMVie-Prom

XhoI
BglII PaeR7I HindIII
GTACGGTGGGAGGTCTATATAAGCAGAGCTGGTTTAGTGAACCGTCAGATCCGCTAGCGCTACCGGACTCAGATCTCGAGCTCAAGCTTG 2790
CATGCCACCCTCCAGATATATTCGTCTCGACCAAATCACTTGGCAGTCTAGGCGATCGCGATGGCCTGAGTCTAGAGCTCGAGTTCGAAC
CMVie-Prom

ClaI
NotI SalI StuI BspDI
TTTGGCCGAGGCGGCCGCTTGTCGACAGGCCTTAATGGCCTAACATCGATAAAATAAAAGATTTTATTTAGTCTCCAGAAAAAGGGGGGA 2880
AAACCGGCTCCGCCGGCGAACAGCTGTCCGGAATTACCGGATTGTAGCTATTTTATTTTCTAAAATAAATCAGAGGTCTTTTTCCCCCCT

ATGAAAGACCCCACCTGTAGGTTTGGCAAGCTAGCTTAAGTAACGCCATTTTGCAAGGCATGGAAAAATACATAACTGAGAATAGAGAAG 2970
TACTTTCTGGGGTGGACATCCAAACCGTTCGATCGAATTCATTGCGGTAAAACGTTCCGTACCTTTTTATGTATTGACTCTTATCTCTTC

TTCAGATCAAGGTCAGGAACAGATGGAACAGCTGAATATGGGCCAAACAGGATATCTGTGGTAAGCAGTTCCTGCCCCGGCTCAGGGCCA 3060
AAGTCTAGTTCCAGTCCTTGTCTACCTTGTCGACTTATACCCGGTTTGTCCTATAGACACCATTCGTCAAGGACGGGGCCGAGTCCCGGT

AGAACAGATGGAACAGCTGAATATGGGCCAAACAGGATATCTGTGGTAAGCAGTTCCTGCCCCGGCTCAGGGCCAAGAACAGATGGTCCC 3150
TCTTGTCTACCTTGTCGACTTATACCCGGTTTGTCCTATAGACACCATTCGTCAAGGACGGGGCCGAGTCCCGGTTCTTGTCTACCAGGG

XbaI
CAGATGCGGTCCAGCCCTCAGCAGTTTCTAGAGAACCATCAGATGTTTCCAGGGTGCCCCAAGGACCTGAAATGACCCTGTGCCTTATTT 3240
GTCTACGCCAGGTCGGGAGTCGTCAAAGATCTCTTGGTAGTCTACAAAGGTCCCACGGGGTTCCTGGACTTTACTGGGACACGGAATAAA

GAACTAACCAATCAGTTCGCTTCTCGCTTCTGTTCGCGCGCTTCTGCTCCCCGAGCTCAATAAAAGAGCCCACAACCCCTCACTCGGGGC 3330
CTTGATTGGTTAGTCAAGCGAAGAGCGAAGACAAGCGCGCGAAGACGAGGGGCTCGAGTTATTTTCTCGGGTGTTGGGGAGTGAGCCCCG

GCCAGTCCTCCGATTGACTGAGTCGCCCGGGTACCCGTGTATCCAATAAACCCTCTTGCAGTTGCATCCGACTTGTGGTCTCGCTGTTCC 3420
CGGTCAGGAGGCTAACTGACTCAGCGGGCCCATGGGCACATAGGTTATTTGGGAGAACGTCAACGTAGGCTGAACACCAGAGCGACAAGG

TTGGGAGGGTCTCCTCTGAGTGATTGACTACCCGTCAGCGGGGGTCTTTCATTTGGGGGCTCGTCCGGGATCGGGAGACCCCTGCCCAGG 3510
AACCCTCCCAGAGGAGACTCACTAACTGATGGGCAGTCGCCCCCAGAAAGTAAACCCCCGAGCAGGCCCTAGCCCTCTGGGGACGGGTCC

GACCACCGACCCACCACCGGGAGGTAAGCTGGCTGCCTCGCGCGTTTCGGTGATGACGGTGAAAACCTCTGACACATGCAGCTCCCGGAG 3600
CTGGTGGCTGGGTGGTGGCCCTCCATTCGACCGACGGAGCGCGCAAAGCCACTACTGCCACTTTTGGAGACTGTGTACGTCGAGGGCCTC

ACGGTCACAGCTTGTCTGTAAGCGGATGCCGGGAGCAGACAAGCCCGTCAGGGCGCGTCAGCGGGTGTTGGCGGGTGTCGGGGCGCAGCC 3690
TGCCAGTGTCGAACAGACATTCGCCTACGGCCCTCGTCTGTTCGGGCAGTCCCGCGCAGTCGCCCACAACCGCCCACAGCCCCGCGTCGG

BstZ17I

ATGACCCAGTCACGTAGCGATAGCGGAGTGTATACTGGCTTAACTATGCGGCATCAGAGCAGATTGTACTGAGAGTGCACCATATGCGGT 3780
TACTGGGTCAGTGCATCGCTATCGCCTCACATATGACCGAATTGATACGCCGTAGTCTCGTCTAACATGACTCTCACGTGGTATACGCCA

GTGAAATACCGCACAGATGCGTAAGGAGAAAATACCGCATCAGGCGCTCTTCCGCTTCCTCGCTCACTGACTCGCTGCGCTCGGTCGTTC 3870
CACTTTATGGCGTGTCTACGCATTCCTCTTTTATGGCGTAGTCCGCGAGAAGGCGAAGGAGCGAGTGACTGAGCGACGCGAGCCAGCAAG

PciI
AflIII

GGCTGCGGCGAGCGGTATCAGCTCACTCAAAGGCGGTAATACGGTTATCCACAGAATCAGGGGATAACGCAGGAAAGAACATGTGAGCAA 3960
CCGACGCCGCTCGCCATAGTCGAGTGAGTTTCCGCCATTATGCCAATAGGTGTCTTAGTCCCCTATTGCGTCCTTTCTTGTACACTCGTT

AAGGCCAGCAAAAGGCCAGGAACCGTAAAAAGGCCGCGTTGCTGGCGTTTTTCCATAGGCTCCGCCCCCCTGACGAGCATCACAAAAATC 4050
TTCCGGTCGTTTTCCGGTCCTTGGCATTTTTCCGGCGCAACGACCGCAAAAAGGTATCCGAGGCGGGGGGACTGCTCGTAGTGTTTTTAG

GACGCTCAAGTCAGAGGTGGCGAAACCCGACAGGACTATAAAGATACCAGGCGTTTCCCCCTGGAAGCTCCCTCGTGCGCTCTCCTGTTC 4140
CTGCGAGTTCAGTCTCCACCGCTTTGGGCTGTCCTGATATTTCTATGGTCCGCAAAGGGGGACCTTCGAGGGAGCACGCGAGAGGACAAG

CGACCCTGCCGCTTACCGGATACCTGTCCGCCTTTCTCCCTTCGGGAAGCGTGGCGCTTTCTCATAGCTCACGCTGTAGGTATCTCAGTT 4230
GCTGGGACGGCGAATGGCCTATGGACAGGCGGAAAGAGGGAAGCCCTTCGCACCGCGAAAGAGTATCGAGTGCGACATCCATAGAGTCAA

CGGTGTAGGTCGTTCGCTCCAAGCTGGGCTGTGTGCACGAACCCCCCGTTCAGCCCGACCGCTGCGCCTTATCCGGTAACTATCGTCTTG 4320
GCCACATCCAGCAAGCGAGGTTCGACCCGACACACGTGCTTGGGGGGCAAGTCGGGCTGGCGACGCGGAATAGGCCATTGATAGCAGAAC

AGTCCAACCCGGTAAGACACGACTTATCGCCACTGGCAGCAGCCACTGGTAACAGGATTAGCAGAGCGAGGTATGTAGGCGGTGCTACAG 4410
TCAGGTTGGGCCATTCTGTGCTGAATAGCGGTGACCGTCGTCGGTGACCATTGTCCTAATCGTCTCGCTCCATACATCCGCCACGATGTC

AGTTCTTGAAGTGGTGGCCTAACTACGGCTACACTAGAAGGACAGTATTTGGTATCTGCGCTCTGCTGAAGCCAGTTACCTTCGGAAAAA 4500
TCAAGAACTTCACCACCGGATTGATGCCGATGTGATCTTCCTGTCATAAACCATAGACGCGAGACGACTTCGGTCAATGGAAGCCTTTTT

GAGTTGGTAGCTCTTGATCCGGCAAACAAACCACCGCTGGTAGCGGTGGTTTTTTTGTTTGCAAGCAGCAGATTACGCGCAGAAAAAAAG 4590
CTCAACCATCGAGAACTAGGCCGTTTGTTTGGTGGCGACCATCGCCACCAAAAAAACAAACGTTCGTCGTCTAATGCGCGTCTTTTTTTC

GATCTCAAGAAGATCCTTTGATCTTTTCTACGGGGTCTGACGCTCAGTGGAACGAAAACTCACGTTAAGGGATTTTGGTCATGAGATTAT 4680
CTAGAGTTCTTCTAGGAAACTAGAAAAGATGCCCCAGACTGCGAGTCACCTTGCTTTTGAGTGCAATTCCCTAAAACCAGTACTCTAATA

CAAAAAGGATCTTCACCTAGATCCTTTTAAATTAAAAATGAAGTTTTAAATCAATCTAAAGTATATATGAGTAAACTTGGTCTGACAGTT 4770
GTTTTTCCTAGAAGTGGATCTAGGAAAATTTAATTTTTACTTCAAAATTTAGTTAGATTTCATATATACTCATTTGAACCAGACTGTCAA
Amp

ACCAATGCTTAATCAGTGAGGCACCTATCTCAGCGATCTGTCTATTTCGTTCATCCATAGTTGCCTGACTCCCCGTCGTGTAGATAACTA 4860
TGGTTACGAATTAGTCACTCCGTGGATAGAGTCGCTAGACAGATAAAGCAAGTAGGTATCAACGGACTGAGGGGCAGCACATCTATTGAT
Amp

CGATACGGGAGGGCTTACCATCTGGCCCCAGTGCTGCAATGATACCGCGAGACCCACGCTCACCGGCTCCAGATTTATCAGCAATAAACC 4950
GCTATGCCCTCCCGAATGGTAGACCGGGGTCACGACGTTACTATGGCGCTCTGGGTGCGAGTGGCCGAGGTCTAAATAGTCGTTATTTGG
Amp

AseI

AGCCAGCCGGAAGGGCCGAGCGCAGAAGTGGTCCTGCAACTTTATCCGCCTCCATCCAGTCTATTAATTGTTGCCGGGAAGCTAGAGTAA 5040
TCGGTCGGCCTTCCCGGCTCGCGTCTTCACCAGGACGTTGAAATAGGCGGAGGTAGGTCAGATAATTAACAACGGCCCTTCGATCTCATT
Amp

FspI

GTAGTTCGCCAGTTAATAGTTTGCGCAACGTTGTTGCCATTGCTGCAGGCATCGTGGTGTCACGCTCGTCGTTTGGTATGGCTTCATTCA 5130
CATCAAGCGGTCAATTATCAAACGCGTTGCAACAACGGTAACGACGTCCGTAGCACCACAGTGCGAGCAGCAAACCATACCGAAGTAAGT
Amp

GCTCCGGTTCCCAACGATCAAGGCGAGTTACATGATCCCCCATGTTGTGCAAAAAAGCGGTTAGCTCCTTCGGTCCTCCGATCGTTGTCA 5220
CGAGGCCAAGGGTTGCTAGTTCCGCTCAATGTACTAGGGGGTACAACACGTTTTTTCGCCAATCGAGGAAGCCAGGAGGCTAGCAACAGT
Amp

GAAGTAAGTTGGCCGCAGTGTTATCACTCATGGTTATGGCAGCACTGCATAATTCTCTTACTGTCATGCCATCCGTAAGATGCTTTTCTG 5310
CTTCATTCAACCGGCGTCACAATAGTGAGTACCAATACCGTCGTGACGTATTAAGAGAATGACAGTACGGTAGGCATTCTACGAAAAGAC
Amp

ScaI

TGACTGGTGAGTACTCAACCAAGTCATTCTGAGAATAGTGTATGCGGCGACCGAGTTGCTCTTGCCCGGCGTCAACACGGGATAATACCG 5400
ACTGACCACTCATGAGTTGGTTCAGTAAGACTCTTATCACATACGCCGCTGGCTCAACGAGAACGGGCCGCAGTTGTGCCCTATTATGGC
Amp

XmnI

CGCCACATAGCAGAACTTTAAAAGTGCTCATCATTGGAAAACGTTCTTCGGGGCGAAAACTCTCAAGGATCTTACCGCTGTTGAGATCCA 5490
GCGGTGTATCGTCTTGAAATTTTCACGAGTAGTAACCTTTTGCAAGAAGCCCCGCTTTTGAGAGTTCCTAGAATGGCGACAACTCTAGGT
Amp

GTTCGATGTAACCCACTCGTGCACCCAACTGATCTTCAGCATCTTTTACTTTCACCAGCGTTTCTGGGTGAGCAAAAACAGGAAGGCAAA 5580
CAAGCTACATTGGGTGAGCACGTGGGTTGACTAGAAGTCGTAGAAAATGAAAGTGGTCGCAAAGACCCACTCGTTTTTGTCCTTCCGTTT
Amp

SspI

ATGCCGCAAAAAAGGGAATAAGGGCGACACGGAAATGTTGAATACTCATACTCTTCCTTTTTCAATATTATTGAAGCATTTATCAGGGTT 5670
TACGGCGTTTTTTCCCTTATTCCCGCTGTGCCTTTACAACTTATGAGTATGAGAAGGAAAAAGTTATAATAACTTCGTAAATAGTCCCAA
Amp

ATTGTCTCATGAGCGGATACATATTTGAATGTATTTAGAAAAATAAACAAATAGGGGTTCCGCGCACATTTCCCCGAAAAGTGCCACCTG 5760
TAACAGAGTACTCGCCTATGTATAAACTTACATAAATCTTTTTATTTGTTTATCCCCAAGGCGCGTGTAAAGGGGCTTTTCACGGTGGAC

ACGTCTAAGAAACCATTATTATCATGACATTAACCTATAAAAATAGGCGTATCACGAGGCCCTTTCGTCTTCAAGAATTCATACCAGATC 5850
TGCAGATTCTTTGGTAATAATAGTACTGTAATTGGATATTTTTATCCGCATAGTGCTCCGGGAAAGCAGAAGTTCTTAAGTATGGTCTAG

ACCGAAAACTGTCCTCCAAATGTGTCCCCCTCACACTCCCAAATTCGCGGGCTTCTGCCTCTTAGACCACTCTACCCTATTCCCCACACT 5940
TGGCTTTTGACAGGAGGTTTACACAGGGGGAGTGTGAGGGTTTAAGCGCCCGAAGACGGAGAATCTGGTGAGATGGGATAAGGGGTGTGA

SacII

CACCGGAGCCAAAGCCGCGGCCCTTCCGTTTCTTTGCT 3′
••• 5978
GTGGCCTCGGTTTCGGCGCCGGGAAGGCAAAGAAACGA 5′

**DNA Type:**	Synthetic DNA
**Laboratory Host:**	
**Bacterial Transformation Strain:**	Unspecified<br>Dam^+ Dcm^+ $EcoKI^+$
**Description:**	Ligation of Fragment 2 into Fragment 2
**Created:**	Samstag, 10. Aug 2024
**Last Modified:**	Samstag, 10. Aug 2024
**Accession Number:**	
**Code Number:**	
**Sequence Author:**	
**Comments:**	
**References:**	
**Embedded Files:**	
