# Supplementary material for: Truncated mini LRP1 transports cargo from luminal to basolateral side across the blood brain barrier
Source: Fluids Barriers CNS. 2024 Sep 17;21:74. doi: 10.1186/s12987-024-00573-1 (PMC11409491; doi:10.1186/s12987-024-00573-1)
Supplement: Supplementary file 7 — Supplementary Material 7 [file 12987_2024_573_MOESM7_ESM.docx]

**Supplementary Data**

**Material and Methods**

**Table S1: Primary antibodies used for immunofluorescence and western blot**

| Antibody | Organism | Origin | REF# | Dilution |
| --- | --- | --- | --- | --- |
| anti-Clathrin | rabbit | abcam | Ab271185 | 8 μg/ml |
| anti-Caveolin-1 | rabbit | St. John’s Laboratory | STJ92050 | 8 μg/ml |
| anti-EEA-1 | rabbit | abcam | Ab2900 | 10 μg/ml |
| anti-Lamp-1 | rabbit | abcam | Ab24170 | 4μg/ml |
| anti-TfR-1 | mouse | invitrogen | 13-6890 | 10μg/ml |
| anti-Rab27a | mouse | abcam | Ab55667 | 8 μg/ml |
| anti-p-catenin | mouse | BD Biosciences | 610133 | 8 μg/ml |
| Alexa Fluor 488 anti-Myc | mouse | Abcam | Ab202008 | 10 mg/ml |
| Anti-collagen IV | rabbit | BioRad | 148826 | 1:200 |
| Anti-Pecam CD31 | rat | R&D Systems | AF3628 | 1:50 |
| Anti-aquaporin 4 | rabbit | Sigma-Aldrich | AB3594 | 2 μg/ml |
| Alexa Fluor 488 anti-Myc | mouse | Abcam | Ab202008 | 10 μg/ml |
| Alexa Fluor 555  Anti-Myc | mouse | Invitrogen | MA1-980-A555 | 2 μg/ml |
| Anti-Myc (9E10) | mouse | Own production |  | 1:500 |
| Anti-HA | rat | Roche | 11867423001 | 10 mg/ml |
| Anti-tubulin | mouse | Invitrogen | 62204 | 0.5 µg/mL |
| Anti-LRP1 B411E2  (full-length - α-chain)  [1] | mouse | Own production |  | 1:500 |
| Anti-LRP1 1704  (ß-chain) | rabbit | Own production |  | 1:1000 |

**Table S2: Secondary antibodies used for immunofluorescence**

| Antibody | Organism | | Origin | REF# | | Dilution |  |
| --- | --- | --- | --- | --- | --- | --- | --- |
| anti-mouse | chicken | Invitrogen | | | SA1-72018 | 1:1000 in TBS-T/5%-milk | |
| Alexa Fluor 488-anti-Chicken | goat | Abcam | | | ab150169 | 1:15000 in TBS-T | |
| Alexa Fluor 488-anti-Rabbit | goat | Abcam | | | ab150077 | 1:15000 in TBS-T | |
| Alexa Fluor 488-anti-Mouse | goat | Abcam | | | ab150117 | 2 μg/ml | |
| Alexa-Fluor 568- anti-Chicken | goat | Invitrogen | | | A11041 | 2 μg/ml | |
| Alexa-Fluor 352- anti-Mouse | goat | Invitrogen | | | A11045 | 2 μg/ml | |
| Alexa Fluor 647-anti-Rat | goat | Abcam | | | ab150159 | 2 μg/ml | |
| Alexa Fluor 647  Anti-Rabbit | goat | Invitrogen | | | A21245 | 2 μg/ml | |
| Alexa Fluor 488  Anti-Rat | goat | Abcam | | | ab150157 | 2 μg/ml | |
| DAPI |  | Invitrogen | | | D1306 | 0.2 μg/ml | |

***AAV production***

***Cloning of mLRP1_DIV* construct into the pAAV vector***

The mLRP1_DIV* transgene was prepared from human LRP1 cDNA sequence. The mLRP1_DIV* mini-receptor contains a specific signal peptide (residues 1-19; amino acid sequence of the entry no. Q07954 in the UniProt database), the first five amino acids of the mature protein (5AA linker sequence; residues 20-24), a truncated ligand binding domain IV (residues: 3739-3778), C-terminus of 515 kDa subunit (α-chain; residues 3779-3943) and a full 85 kDa ß-subunit of human LRP1 receptor (β-chain; residues 3944-4544). The pAAV-CMV-teto2-LUC backbone plasmid was generously provided by Jakob Körbelin (UKE, Hamburg; Figure S1A). mLRP1_DIV* transgene was amplified from a pcDNA3.1(+) vector in a PCR reaction (list of primers: Table S3). Column-purified (NucleoSpin® Gel and PCR Clean-up, #740609.50, Macherey-Nagel, Düren, Germany) PCR products were then validated by the agarose gel electrophoresis and subsequently ligated with the linearized pAAV-CMV-teto2 backbone devoid of luciferase transgene, using T4 DNA ligase (#M0202S, NEB, Ipswich, MA, USA) in an overnight (o/n) incubation at 16°C. On the next day, ligated plasmids were transformed into chemically competent NEB® Stable Competent E. coli (#C3040I) according to the manufacturer’s protocol. Selected clones were inoculated in 3 mL or 300 mL of Luria Bertani medium for small- and large-scale production, respectively. Bacteria growth was monitored by repeated optical density (OD600) measurements using the UV-Vis spectrophotometer (NanoDrop™ One, ThermoFisher). The pAAV-CMV-teto2-mLRP1_DIV* plasmid (Figure S1B) was isolated from small culture using High Pure Plasmid Isolation Kit (#11754777001). Correct integrity of the construct as well as the integrity of Inverted Terminal Repeats (ITR) was validated by restriction enzyme digestion and agarose gel electrophoresis. Then, plasmids from large-scale production culture were isolated and purified using NucleoBond™ Xtra Midi Kit (#740410.50, Macherey-Nagel), according to manufacturers’ protocols. Finally, the quality and purity of isolated DNA were validated by agarose gel electrophoresis and spectrophotometric measurements. Purified pAAV-CMV-teto2- mLRP1_DIV* plasmid was verified by Sanger sequencing prior to functional assays.

**Table S3: Primers used for AAV production**

| Primer name | Sequence (5’ to 3’) | T_m_ [°C] | GC [%] |
| --- | --- | --- | --- |
| PCR |  |  |  |
| PmeI Kozak mLRP1_DIV* | CAGGTTTAAACGCCACCATGCTGACC | 59 | 54 |
| XbaI HA mLRP1_DIV* | GGTTCTCTAGACTAAGCGTAATCTGG | 59 | 46 |
| Sequencing |  |  |  |
| mLRP1_DIV*seq rev | GCACGTGCGGCACAGAAACTCCTTC | 56 | 60 |
| LRP1CT fwd | TTCGGGATCCAAGCGGCTGGACAAC | 52 | 60 |
| LRP1CT seq fwd | CTGGTATAAGCGGCGAGTC | 58 | 58 |
| ssDNA oligo |  |  |  |
| HA-tag | TACCCATACGATGTTCCAGATTACGCT |  |  |
| Myc-tag | GAACAAAAACTTATTTCTGAAGAAGATCTG |  |  |


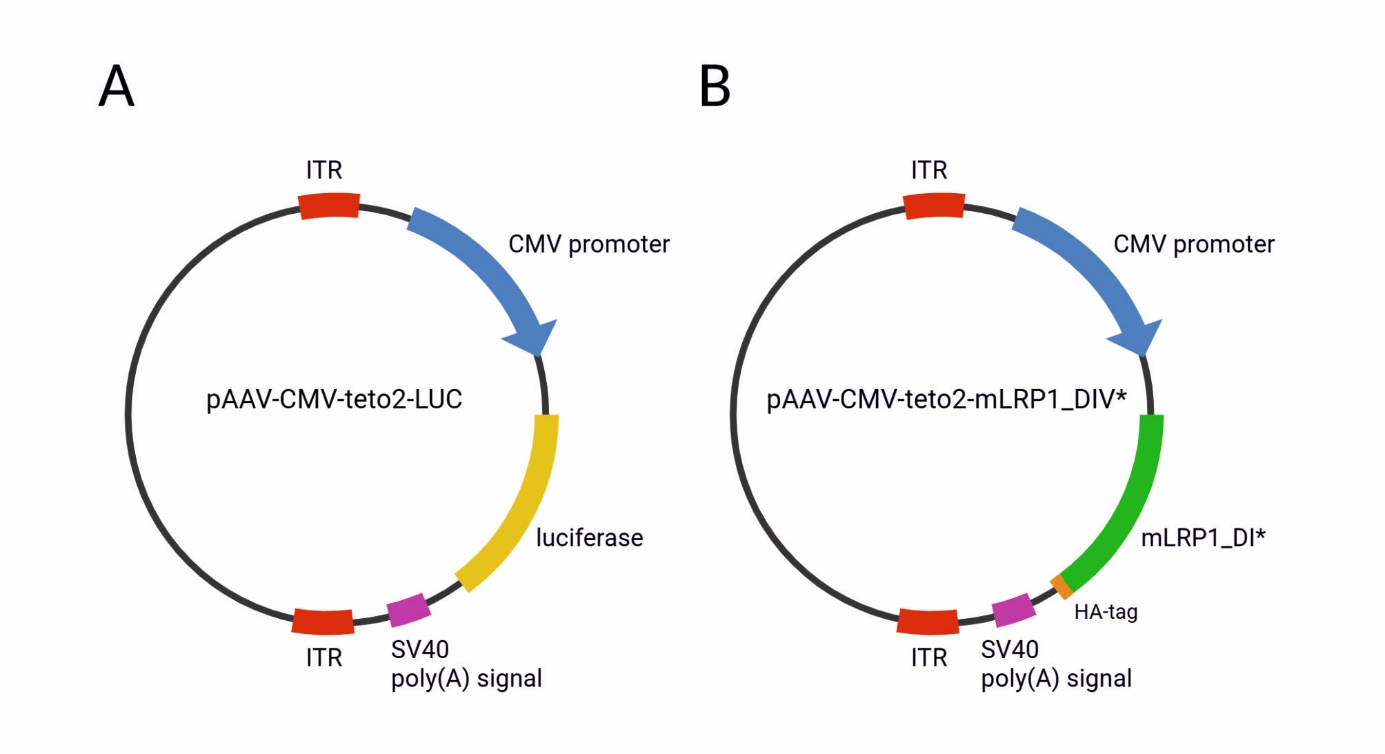


**Figure S1: Cloning strategy for the pAAV-CMV- teto2-mLRP1_DIV* DNA vector.**

(A) pAAV-CMV-teto2- LUC expression cassette containing luciferase reporter gene under control of the cytomegalovirus (CMV) promoter and SV40 poly-A signal embedded between two modified AAV2 Internal Terminal Repeats (ITR) was used as a vector backbone. (B) pAAV-CMV-teto2-mLRP1_DIV* vector. Luciferase reporter gene was cut out in the restriction digestion with PmeI and XbaI restriction enzymes. To facilitate cloning, restriction sites PmeI and XbaI were introduced upstream and downstream of mLRP1_DIV*, respectively, during the PCR amplification

***AAV production in HEK293T cells***

Recombinant AAV vectors were produced by a transient transfection of HEK293T cells with three distinct plasmids (Figure S2A). Production was performed at the University Medical Center Hamburg-Eppendorf (UKE) by Magdalena Kurtyka together with Dr. Jakob Körbelin [2]. A triple transfection is a well-established, safe method for large scale production of recombinant AAV vectors. Producer cells, in this study HEK293T cells, are transfected with a vector plasmid containing the transgene of interest subcloned between two ITRs, an AAV helper plasmid containing rep and cap genes and an adenoviral helper plasmid which provides genes for adenoviral proteins that are necessary for the AAV replication. Safety is ensured by delivering ITRs in cis orientation together with the transgene, while rep and cap genes are delivered in trans orientation with the AAV helper plasmid (Figure S2B). Briefly, HEK293T cells were seeded in 145 mm culture dishes at the density 1x107 cells per dish. Next day at 60-70% confluency, cells were transfected with plasmid DNA vectors using the Polyfect Transfection Reagent (Qiagen). The pXX6 adenoviral helper plasmid (containing E1A, E1B, E2A, E4-orf6 and VA genes), the pXX2-187-NRGTEWD plasmid (encoding the modified, brain endothelium-specific AAV capsid) and the pAAV-CMV-teto2-mLRP1_DIV* plasmid were used in a ratio of 6:3:3 (µg per dish), respectively. Purification of rAAV using iodixanol density-gradient ultracentrifugation 72 hours post-transfection, cells were harvested and lysed in three freeze-thaw cycles in PBS supplemented with 1 mM MgCl2 and 2.5 mM CaCl2. Next, recombinant vectors were purified by the iodixanol density-gradient ultracentrifugation. Briefly, discontinuous iodixanol gradient (15, 25, 40 and 54%) was added subsequently to Quick-Seal Ultraclear Centrifuge tubes (Beckman) containing the harvested recombinant AAVs. Solution was ultracentrifuged using 70.1 Ti rotor (Beckman) at 350,000 g for 70 min at 18 °C. Purified viral particles were aspirated with an 18 G needle (Braun) from a layer containing 40% iodixanol and subsequently dialysed against HBBS.


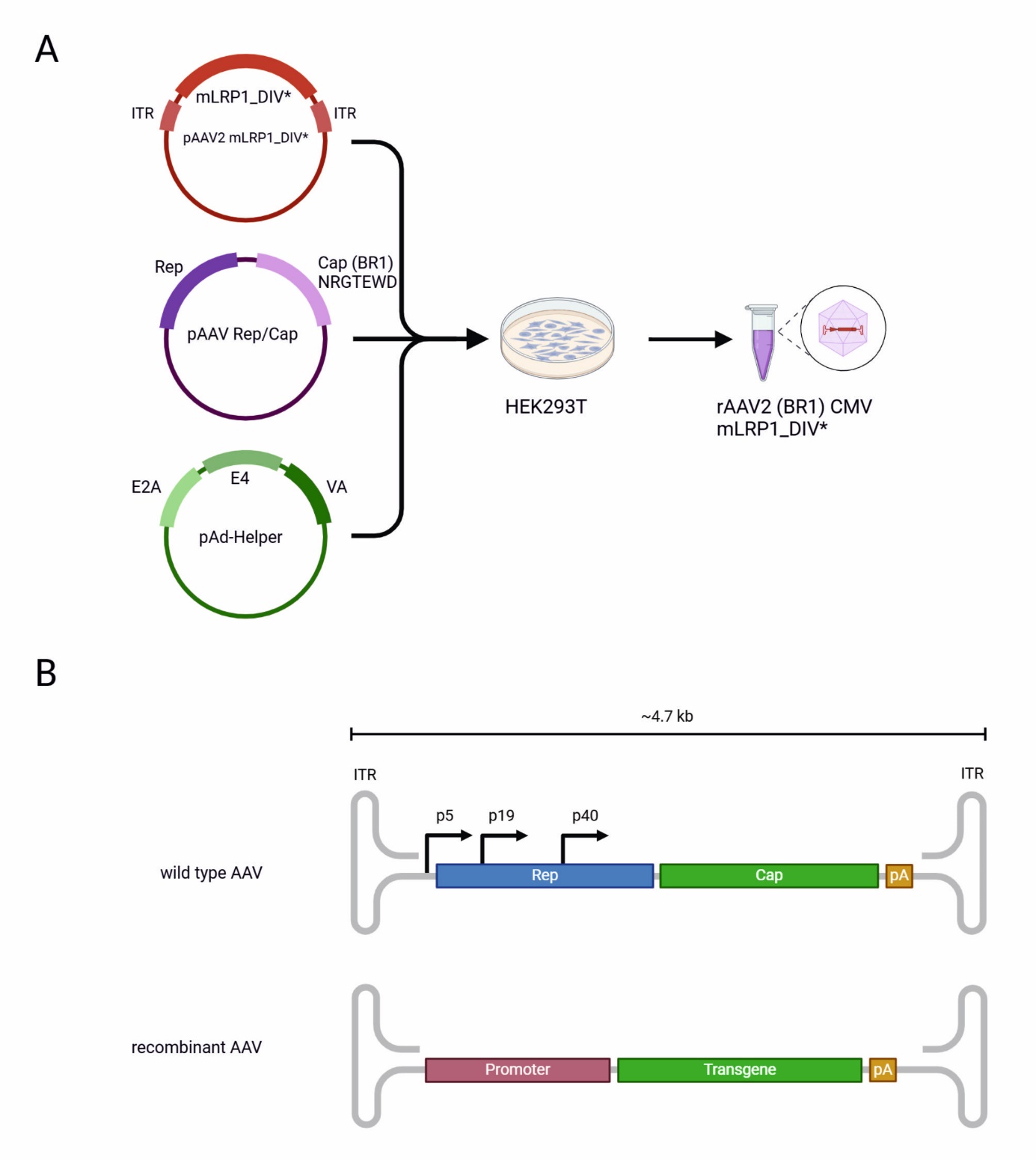


**Figure S2: Recombinant AAV vectors**. (A) AAV vector production using a triple-transfection method in HEK293T packaging cells. (B) Genome organization of wild type and recombinant AAV vectors. In a wild type AAV, all viral elements required for encapsidation into viral particles are in cis orientation. In the recombinant AAV vectors production, the ITRs are the solely viral elements required in cis for encapsidation of the transgene, while rep and cap genes are delivered in trans with a helper plasmid. Such an approach allows for a complete exchange of AAV genome’s coding region and its promoters and ensures safety of the procedure.

***Insertion of the hemagglutinin* and MYC *tag***

A short ssDNA oligomer encoding for the hemagglutinin (HA) or Myc epitope was inserted downstream or upstream of the mLRP1_DIV* construct between last base pair (bp) of LRP1’s cytoplasmatic tail and the stop codon or after the signal peptide sequence at the N-terminus. Briefly, the pAAV-CMV-teto2-mLRP1_DIV* plasmid was linearized by restriction digestion and dephosphorylated using quick CIP alkaline phosphatase (NEB, #M0525). ssDNA HA-tag or Myc -tag oligomer (Table S3) was phosphorylated using T4 Polynucleotide Kinase (T4 PNK) (10 U/µl) (NEB #M0201). Linearized plasmid and ssDNA oligomer were then ligated using HiFi DNA Assembly Master Mix and transformed into chemically competent NEB® Stable Competent E. coli (#C3040I, NEB) according to the manufacturer’s protocol.***Internalization of antibodies and liposomes***

Internalization of 9E10 was performed in CHO 13-5-1 mLRP1_DIV* or pLBCX cells. Cells were starved for 1h in OPTI-MEM® (Gibco) and internalization was performed for 1h at 37°C. Proteins were extracted and analyzed using SDS-PAGE as described above. Internalization of liposomes was performed in hcMEC/D3 cells 72h post transfection with mLRP1_DIV* or control. Uptake of 3mM
9E10 – IL or unm. - IL was performed for 2h. Afterwards, cells were washed with acidic DPBS and lysed in 1% TritonX-100 for 60 min at 60 °C. Internalized amount of immunoliposomes was analyzed based on fluorescence spectroscopy of rhodamine by the Varioskan LUX multimode microplate reader (SkanIt Software 6.0.2.3) (Ex.: 540nm, Em.: 591 nm).

***Treatment of PS70 cells with liposomal BB25***

To examine the biological activity of BB25 – 9E10 - IL, CHO cells overexpressing human APP751 and wt human Presenilin 1 (PS70) were seeded on 96-well plates. After 24h, cells were treated with 10µM free BB25, 9E10 functionalized immunoliposomes loaded with BB25 (BB25 – 9E10 - IL) or unloaded 9E10 functionalized liposomes (unloaded 9E10 - IL) for 48h. The administered concentration of the liposomes was adjusted to the free BB25. After 48h, the supernatants were collected and centrifuged at 18000 RCF for 20 minutes at 4°C. The γ-secretase activity was investigated by determining the protein levels of Aβ_38_ and Aβ_42_ using a cell-based sandwich enzyme-linked immunosorbent assay (ELISA).

***Influence of liposomal BB25 and liposomes on the integrity of an endothelial barrier***

To analyze the influence of liposomes or the γ-secretase modulator BB25 on the functionality of the BBB of endothelial cells, bEnd.3 cells were transfected with mLRP1_DIV* and seeded on coated cell culture inserts, followed by TEER monitoring. After a monolayer was built, bEnd.3 mLRP1_DIV* cells were divided into four groups similar in TEER and CCI values. Cells were cultured in culture media supplemented with 10µM free BB25, liposomal BB25 (BB25 – 9E10 - IL), unloaded 9E10 functionalized liposomes (unloaded 9E10 - IL) or DMSO for 48h. The administered concentration of the liposomes was adjusted to the free BB25. TEER of the cells was measured automatically every hour under physiological conditions by impedance spectroscopy over 48h. Additionally, the culture media of the luminal compartment was supplemented with 50 µg/mL fluorescein isothiocyanate (FITC)-Dextran
(3-4 kDa) for 24h. To assess paracellular leakage across the endothelial monolayer, fluorescence intensities of FITC-dextran in the abluminal compartments were measured after 24h using the Varioskan LUX multimode microplate reader (SkanIt Software 6.0.2.3) as described above.

***In vitro transcytosis studies with Bafilomycin***

Prior to examination of transcellular transport of anti-Myc antibodies (Alexa Fluor^TM^ 555 (9E10)), mLRP1_DIV* transfected bEnd.3 cells were seeded into transparent membrane inserts (0.4 µm) coated with the coating solution. The next day, inserts were placed into the automated cell monitoring system cellZscope (NanoAnalytics) to monitor the transendothelial electrical resistance (TEER) and capacitance (CCI) of the cells. As CCI reached a value of ~1 µF/cm^2^ or below, cells were stimulated with hydrocortisone (550nM) to enhance tight junction formation. When tight junction formation, barrier function and confluence of the cell monolayer was ensured (TEER > 30 Ω*cm^2^; CCI = ~ 1 µF/cm^2^), mLRP1_DIV* transfected cells were incubated with Bafilomycin (10nM) (InvivoGen; 88899-55-2) for 24 h. The transport of cargo was performed afterwards, approximately 72h post transfection with anti-Myc antibodies (2µg/ml) for 1h. As paracellular leakage marker 50 µg/ml of fluorescein isothiocyanate (FITC)-Dextran (3-4 kDa) was used. As a readout, the medium of the abluminal compartment of all wells was collected and analyzed using fluorescence spectroscopy of FITC or Alexa Fluor 555 by the Varioskan LUX multimode microplate reader (SkanIt Software 6.0.2.3) (Ex.: 495nm / 555nm, Em.: 520nm / 568nm).

***Results***

**mLRP1_DIV* mediated internalization of anti-Myc antibodies in stably transfected CHO 13-5-1 cells**

mLRP1_DIV*’s capability of internalizing anti-Myc antibodies (9E10) was first investigated using stably transfected CHO 13-5-1 cells. Cells were incubated with anti-Myc antibodies for different time periods and mLRP1_DIV*’s mediated uptake of antibodies was analyzed using Western Blot analysis. According to the internalization assays, one band corresponding to mLRP1_DIV* (130 kDa) in CHO 13-5-1 mLRP1_DIV* were immunologically detected, while no bands could be detected in CHO 13-5-1 pLBCX cells. Moreover, bands at about 50 kDa and 25 kDa corresponding to the heavy and light chain of 9E10 became visible in both cell lines that has been incubated with the antibodies (Figure S3 A). Cells incubated with no antibodies showed no bands corresponding to IgG. Regarding cellular internalization of 9E10, CHO 13-5-1 mLRP1_DIV* cells showed a 10-fold or 5-fold higher internalization of 9E10’s heavy chain after 60 min (p = 0.0004) and 30 min (p =0.0305) compared to control (Figure S3 B and D). The uptake of 9E10’s light chain was 3-fold higher after 60 min (p = 0.0302) and 2-fold higher after 30 min (p = 0.0023) compared to control (Figure S3 C and E). Thus, cells expressing the mLRP1_DIV* receptor showed a significant higher internalization of 9E10 compared to the cells lacking the receptor.


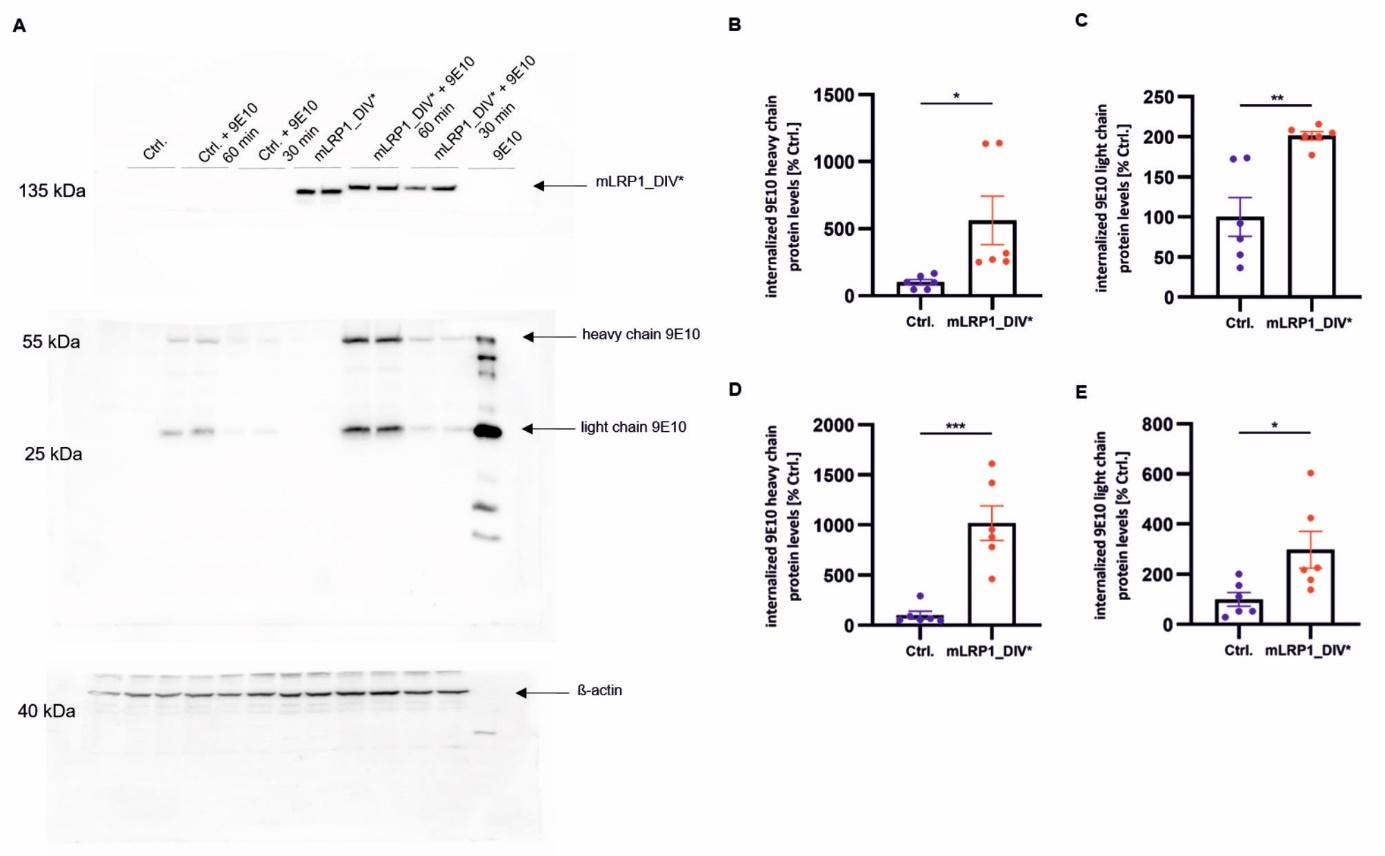


Figure S3: mLRP1_DIV* mediated internalization of anti-Myc antibodies in CHO 13-5-1 cells.

(A) Representative immunoblotting for protein levels in cell lysates of CHO 13-5-1 mLRP1_DIV* and CHO 13-5-1 pLBCX cells being incubated with 30 μg/ml of anti-Myc antibodies (9E10) or no antibodies for (B, C) 30 or (D, E) 60 min at 37°C. (B, D) Heavy and (C, E) light chain protein levels were quantified by densitometric analysis after immunoblotting and normalized to ß-actin. (B – E) The intensities of IgG in CHO 13-5-1 pLBCX cells were defined as 100%. Data represent the mean ± SEM of six individual replicates from n = 3 independent experiments. Unpaired t-test was used for statistical analysis.

***Vesicular trafficking of mIgG and 9E10 across mLRP1_DIV* cells***

As shown previously, anti-Myc antibodies were transported across an *in vitro* model of the BBB. In the following, the vesicular trafficking route of anti-Myc antibodies (9E10) was further investigated. For this purpose, hcMEC/D3 cells were transfected with mLRP1_DIV* and cultured in transparent transmembrane inserts until a monolayer was built. The trafficking route was explored by exposing the luminal side of the cells to 30 µg/ml of 9E10 or mIgG for 60 min. Cells were washed, fixed and the internalized antibodies (9E10) were identified using a fluorescence-labeled anti-mouse IgG. To investigate the cellular transport route of mLRP1_DIV* and 9E10 or mIgG, cells were co-stained for mLRP1_DIV*, the endocytosis marker Clathrin and Caveolin-1, the early endosome using EEA-1, the lysosome via Lamp-1, recycling endosomes using TfR-1 and for the exocytosis marker Rab27a. After incubation of hcMEC/D3 mLRP1_DIV* cells with 9E10 antibodies, a co-localization of mLRP1_DIV* and 9E10 with Clathrin and Caveolin-1 could be detected on the cell surface as well as within the cell. Moreover, only mLRP1_DIV* and not 9E10 appears to co-compartmentalize with early endosome (EEA-1) or lysosome (Lamp-1) positive structures. Regarding TfR-1 and Rab27a, both mLRP1_DIV* as well as 9E10 has been observed to locate near those structures. In general, a continuous co-localization of mLRP1_DIV* and 9E10 could be detected (Figure S4).

**
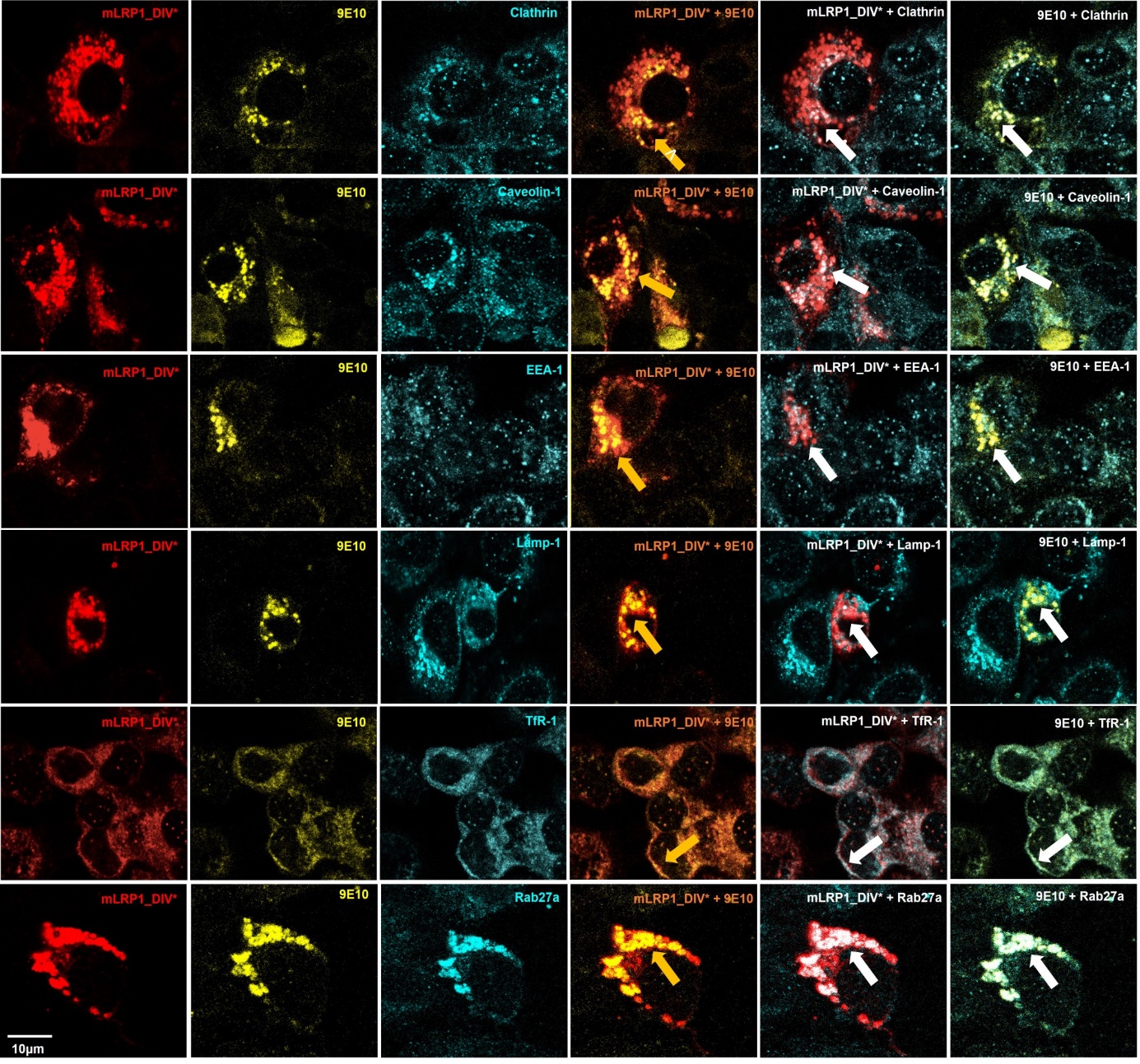
**

Figure S4: Subcellular sorting of 9E10 and mLRP1_DIV* during transport across hcMEC/D3 cells.

Cells were transfected with mLRP1_DIV* and transport of 9E10 was performed 72h post transfection. Representative confocal images of mLRP1_DIV* and 9E10 co-localization with intracellular markers Clathrin, Caveolin-1, EEA-1, Lamp-1, TfR-1 and Rab27a 60 min after transport of 9E10. Cells were washed with acidic PBS, fixed with 4% PFA, permeabilized and stained for mLRP1_DIV*, 9E10 and corresponding intracellular structure (IS). Images were taken with the LSM710 confocal laser scanning microscope using a laser at a wavelength of (mLRP1_DIV*) 647nm, (9E10) 568nm and (IS) 488nm. mLRP1_DIV* is presented in red, 9E10 in yellow and IS in cyan. Co-localizations were investigated by merging two channels. (orange) mLRP1_DIV* + 9E10, (white) mLRP1_DIV* + corresponding IS and (white) 9E10 + corresponding IS. Scale bar = 10 µm.

During the transcytosis of unspecific mIgG, a co-localization of mLRP1_DIV* and mIgG could only be partially detected. However, while mLRP1_DIV* is located within Clathrin, Caveolin-1, EEA-1 and
Lamp-1 positive structures, mIgG could only be detected in close proximity to Lamp-1. Additionally, both, mLRP1_DIV* as well as mIgG could be observed in TfR-1 and Rab27a positive structures
(Figure S5).


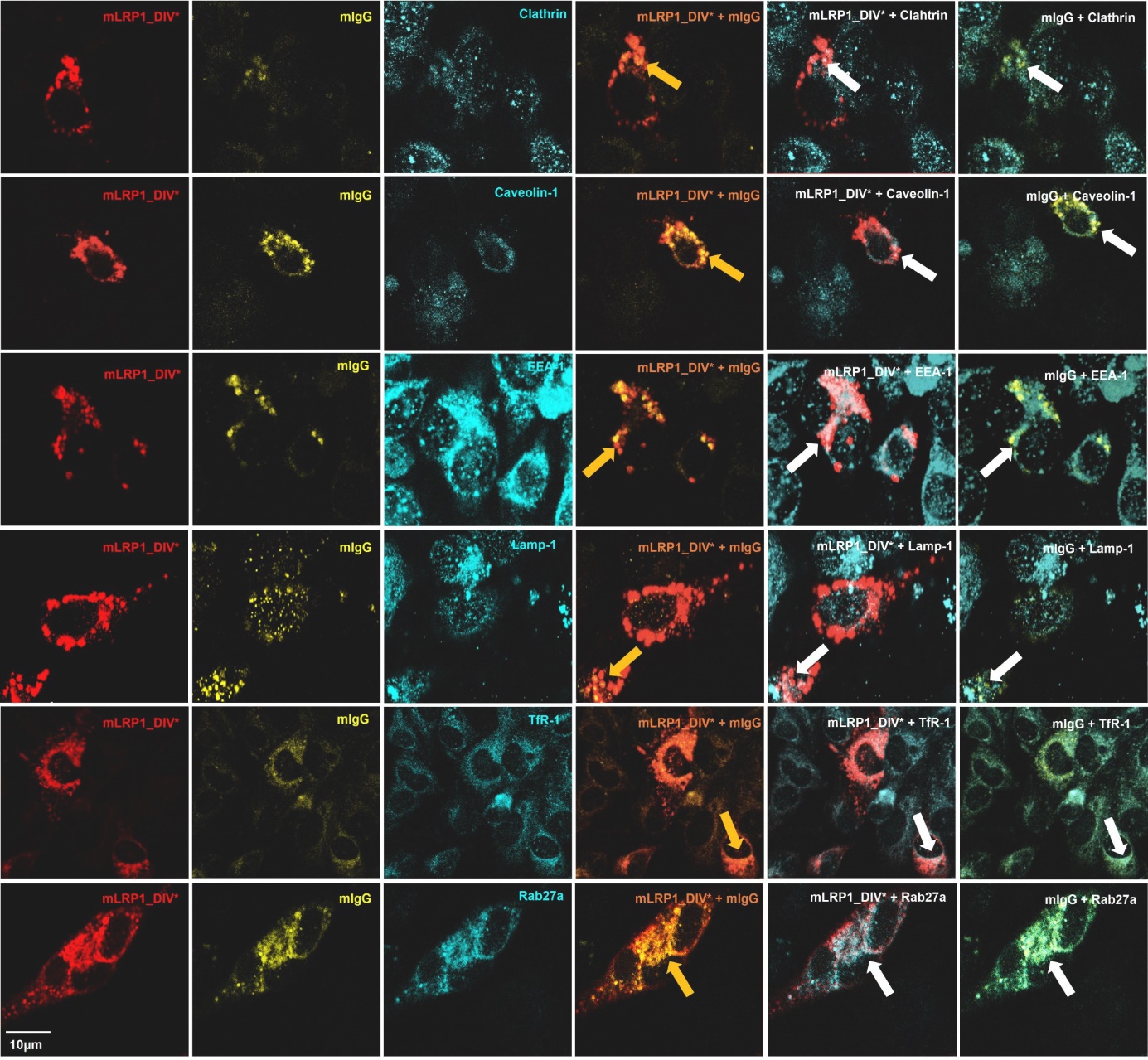


Figure S5: Subcellular sorting of mIgG and mLRP1_DIV* during transport across hcMEC/D3 cells.

Cells were transfected with mLRP1_DIV* and transport of unspecific mIgG was performed 72h post transfection. Representative confocal images of mLRP1_DIV* and mIgG co-localization with intracellular markers Clathrin, Caveolin-1, EEA-1, Lamp-1, TfR-1 and Rab27a 60 min after incubation with mIgG. Cells were washed with acidic PBS, fixed with 4% PFA, permeabilized and stained for mLRP1_DIV*, mIgG and corresponding intracellular structure (IS). Images were taken with the LSM710 confocal laser scanning microscope using a laser at a wavelength of (mLRP1_DIV*) 647nm, (mIgG) 568nm, (IS) 488nm. mLRP1_DIV* is presented in red, mIgG in yellow and IS in cyan. Co-localizations were investigated by merging two channels. (orange) mLRP1_DIV* + mIgG, (white) mLRP1_DIV* + corresponding IS and (white) 9E10 + corresponding IS. Scale bar = 10 µm.

**mLRP1_DIV* mediated internalization of 9E10 functionalized immunoliposomes in transiently transfected hcMEC/D3 cells**

Based on the internalization of 9E10 antibodies in CHO 13-5-1 cells as well as transport of 9E10 across hcMEC/D3 cells, liposomes were functionalized with 9E10 antibodies using a thin film hydration method. Investigation of the internalization of 9E10 functionalized immunoliposomes (9E10 - IL) was performed in transiently transfected hcMEC/D3 cells with mLRP1_DIV* or pLBCX as control. As further control, internalization of unmodified immunoliposomes (unm. - IL) in hcMEC/D3 pLBCX or mLRP1_DIV* cells were included into the experiments. In all experimental groups 3mM of liposomes were added to the cells and the fluorescence intensity of rhodamine, incorporated into the liposomal membrane was measured in cell lysates after 2h (Figure S6 A). The internalization of 9E10 functionalized immunoliposomes by hcMEC/D3 mLRP1_DIV* cells within 2h was significantly increased compared to control cells (p < 0.0001). Additionally, a significant higher endocytosis of 9E10 functionalized liposomes compared to an application of unmodified liposomes to mLRP1_DIV* transfected cells (p < 0.0001) or to control cells (p < 0.0001) could also be observed (Figure S6 A). The internalization of 9E10 functionalized immunoliposomes by hcMEC/D3 mLRP1_DIV* cells was further explored by incubating the cells at 37°C with or without 9E10 antibodies and at 4°C to compete or inhibit the endocytosis of 9E10 functionalized liposomes (Figure S6 B). Thereby, internalization of 9E10 functionalized immunoliposomes after simultaneous application of 9E10 antibodies or incubation at 4°C was significantly reduced by 80% after 2h compared to control (p < 0.0001 / p < 0.0001).


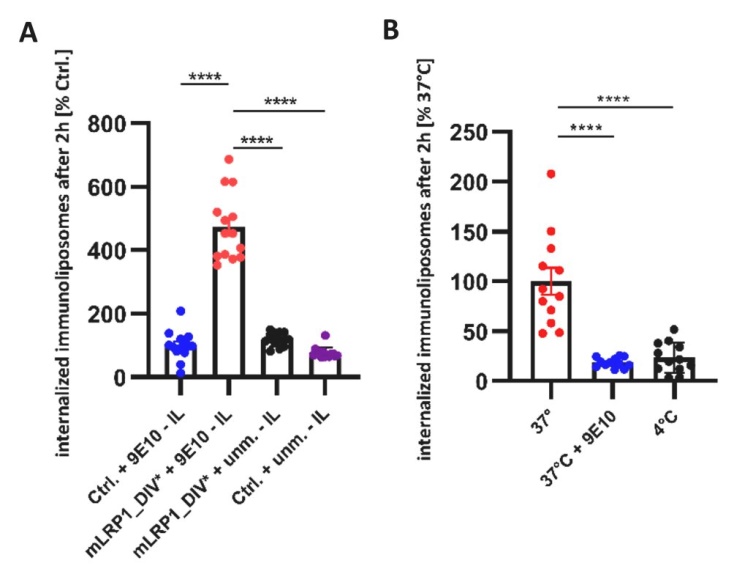


**Figure S6: Cellular internalization of immunoliposomes in hcMEC/D3 cells.**

(A) Cellular internalization of 3mM 9E10 functionalized or unmodified immunoliposomes in hcMEC/D3 mLRP1_DIV* or pLBCX cells after 2h. (B) Cellular internalization of 3mM 9E10 functionalized immunoliposomes in hcMEC/D3 mLRP1_DIV* cells after 2h at 37 °C with or without 9E10 antibodies or at 4°C. Cell lysis was performed afterwards and the fluorescence intensity of rhodamine in cell lysates was measured. To quantify the cellular uptake of liposomes, a calibration curve for both liposomes was generated. Internalized amount of liposomes was calculated according to the calibration curve. (A) hcMEC/D3 pLBCX cells incubated with 9E10 functionalized immunoliposomes or (B) condition of 37°C were defined as 100%. Data represent the mean ± SEM of fourteen (A) or twelve (B) individual replicates from n = 3 independent experiments. One-way ANOVA followed by Tukey’s multiple comparison test was used for statistical analysis.

The cellular uptake of 9E10 functionalized or unmodified liposomes was further confirmed by immunofluorescence. hcMEC/D3 cells were transfected with mLRP1_DIV* or pLBCX followed by an incubation with 3mM of 9E10 functionalized or unmodified liposomes for 2h, 72h post transfection. Cells were washed, fixed, and stained for mLRP1_DIV* and 9E10 using fluorescence-labeled secondary antibodies. Thereby, an internalization of 9E10 functionalized liposomes could only be observed in cells expressing the mini LRP1 receptor, whereas cells lacking the receptor do not show an internalization of 9E10 functionalized liposomes (Figure S7 F). Further, an attachment of 9E10 to liposomes after internalization has been confirmed (Figure S7 E). Thereby, 9E10 is continuously co-localized with liposomes as well as with mLRP1_DIV*, indicating its incorporation into the liposomal membrane, even after internalization (Figure S7 E and G). Thus, immunoliposomes were specifically internalized via mLRP1_DIV* and their co-localization to mLRP1_DIV* confirm their attachment to mLRP1_DIV* even after endocytosis. In contrast to 9E10 functionalized liposomes, unmodified liposomes were internalized by both mLRP1_DIV* and non-transfected hcMEC/D3 cells. Thereby, no co-localization of unmodified liposomes and mLRP1_DIV* could be detected (Figure S7 J).


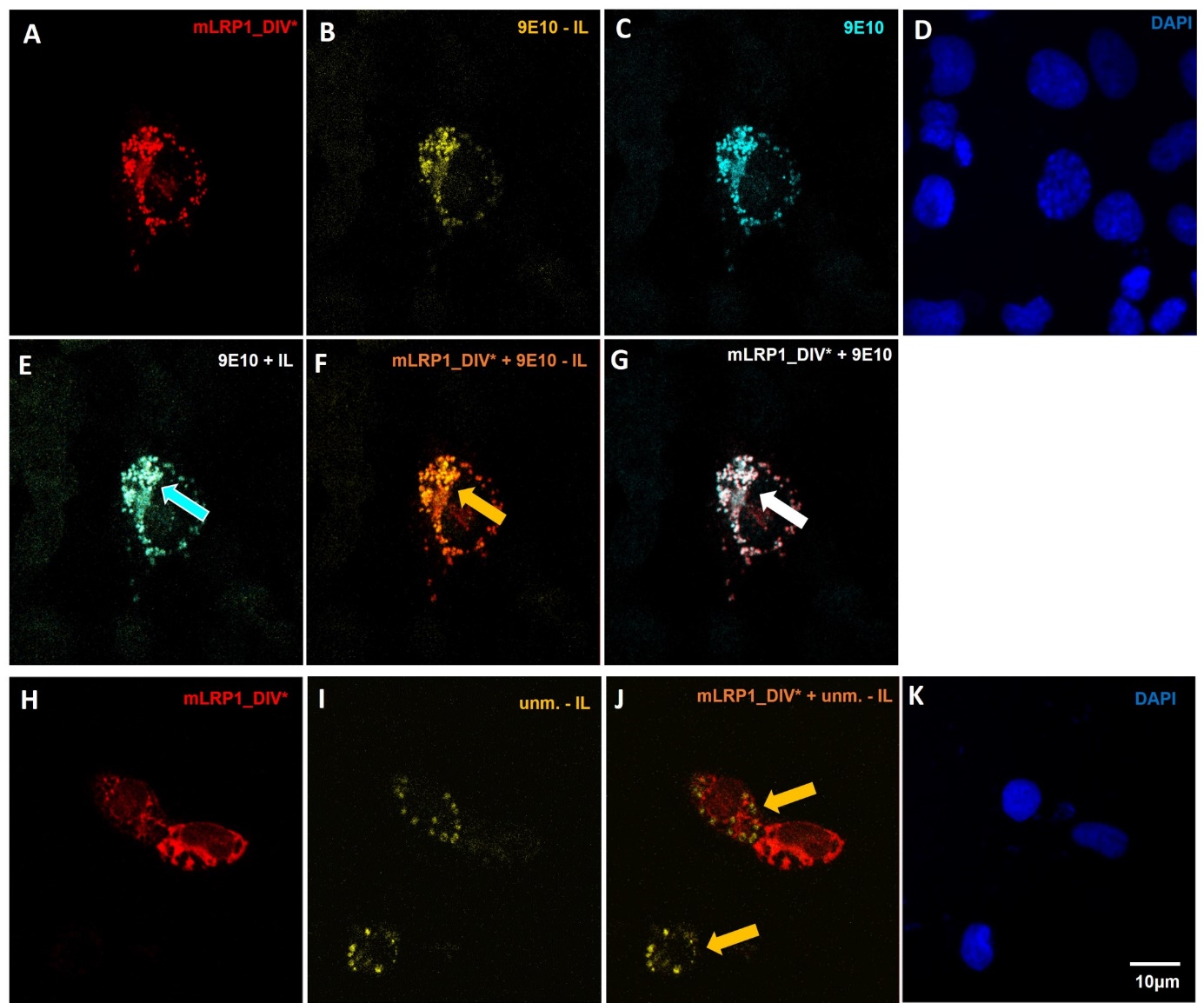


Figure S7: Co-localization of mLRP1_DIV* and 9E10 - IL or unm. – IL after internalization in hcMEC/D3 cells.

Representative confocal images of cellular internalization of 3mM (A-G) 9E10 functionalized immunoliposomes or (H-K) unmodified liposomes in hcMEC/D3 mLRP1_DIV* cells after 2h. Cells were washed with acidic PBS, fixed with 4% PFA, permeabilized and stained for mLRP1_DIV* and 9E10. Images were taken with the LSM710 confocal laser scanning microscope using a laser at a wavelength of (A/H) 647nm, (B/I) 540nm, (C) 488nm and (D/K) 350nm. mLRP1_DIV* is depicted in red, liposomes in yellow, 9E10 in cyan and nuclei in blue. Co-localizations were investigated by (E) merge of B and C, (F) merge of A and B, (G) merge of A and C and (J) merge of H and I. Scale bar = 10 µm.

***Vesicular Trafficking of 9E10 – IL across hcMEC/D3 cells***

The transport of 9E10 functionalized and unmodified immunoliposomes in an *in vitro* model of the BBB has previously been demonstrated. The liposomes' vesicular trafficking pathway was subsequently studied in more detail. To do so, mLRP1_DIV* was transfected into hcMEC/D3 cells, which were then grown in transmembrane inserts until a monolayer was formed. By exposing the luminal side of the cells to 3 mM of 9E10 functionalized or unmodified liposomes for 2h, the trafficking pathway was investigated. Cells were washed, fixed, and stained for the endocytosis and transport marker Clathrin, Caveolin-1, EEA-1, Lamp-1, TfR-1, and Rab27a as well as for mLRP1_DIV*, liposomes and 9E10 using fluorescence-labeled secondary antibodies (Figure S8-S13).

After incubation of hcMEC/D3 mLRP1_DIV* cells with 9E10 functionalized liposomes, a continuous co-localization of 9E10 with liposomes could be observed, indicating an attachment of 9E10 to liposomes during transport. Moreover, 9E10 functionalized liposomes seems to be transported in an mLRP1_DIV* dependent manner due to a constant co-localization. Thereby, both mLRP1_DIV* and liposomes seem to co-compartmentalize with Clathrin, Caveolin-1, Lamp-1, recycling endosomes (TfR-1) as wells as with Rab27a positive vesicles. Moreover, whether mLRP1_DIV* nor 9E10 liposomes could be detected within early endosomes (EEA-1) (Figure S8-S10).


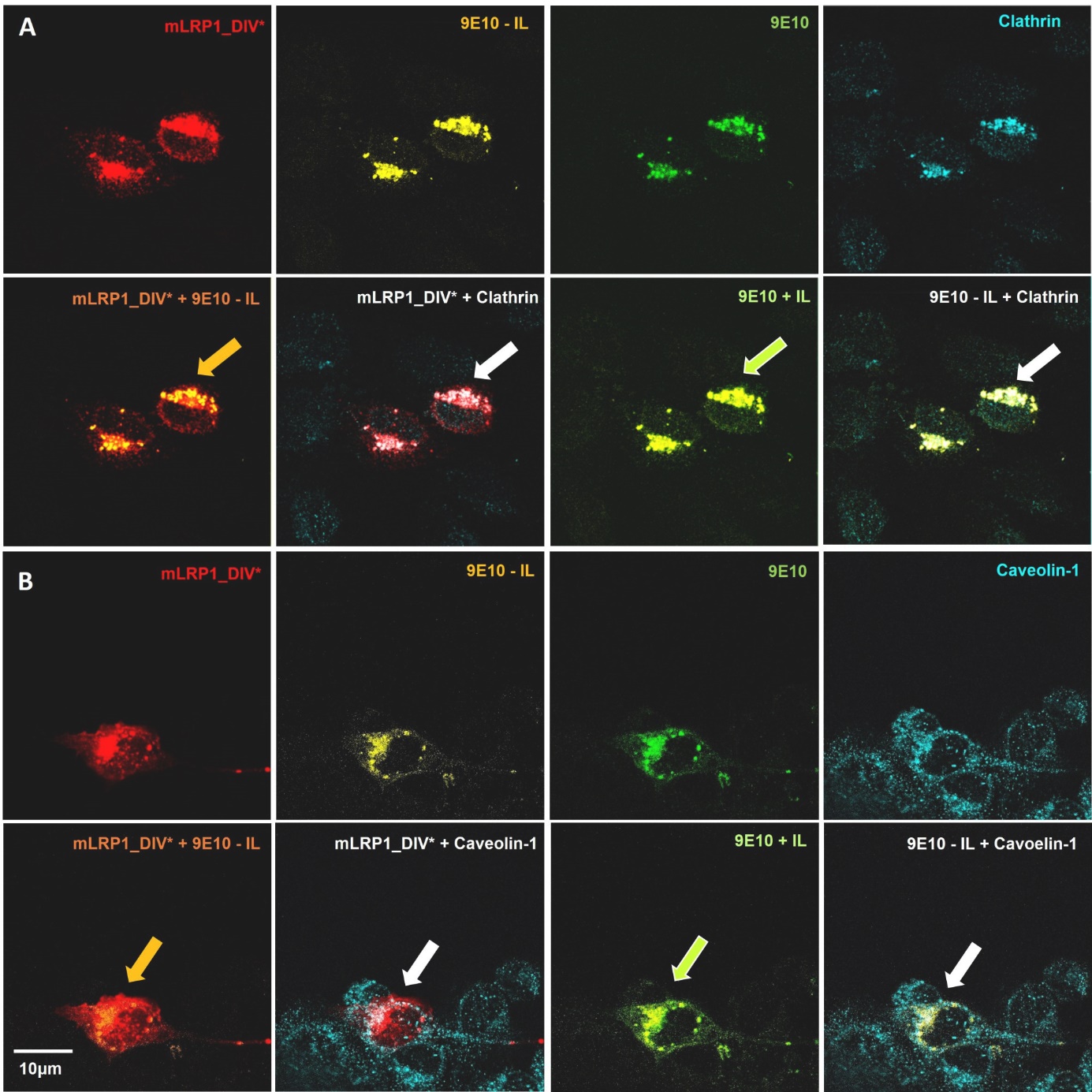


Figure S8: Subcellular sorting of 9E10 - IL/mLRP1_DIV* complex in co-stainings with Clathrin and Caveolin-1 during transport across hcMEC/D3 cells.

Cells were transfected with mLRP1_DIV* and transport of 9E10 - IL was performed 72h post transfection. Representative confocal images of mLRP1_DIV*, liposomes and 9E10 co-localization with intracellular markers (A) Clathrin and (B) Caveolin-1 2h after incubation with the liposomes. Cells were washed with acidic PBS, fixed with 4% PFA, permeabilized and stained for mLRP1_DIV*, 9E10 and corresponding intracellular structure (IS). Images were taken with the LSM710 confocal laser scanning microscope using a laser at a wavelength of (mLRP1_DIV*) 647nm, (rhodamine) 540nm, (9E10) 350nm and (IS) 488nm. mLRP1_DIV* is depicted in red,
9E10 - IL in yellow, 9E10 in green and (IS) in cyan. Co-localizations were investigated by merging two channels. (orange) mLRP1_DIV* + liposomes, (white) mLRP1_DIV* + corresponding IS, (light green) liposomes + 9E10 and (green/white) liposomes + corresponding IS. Scale bar = 10 µm.


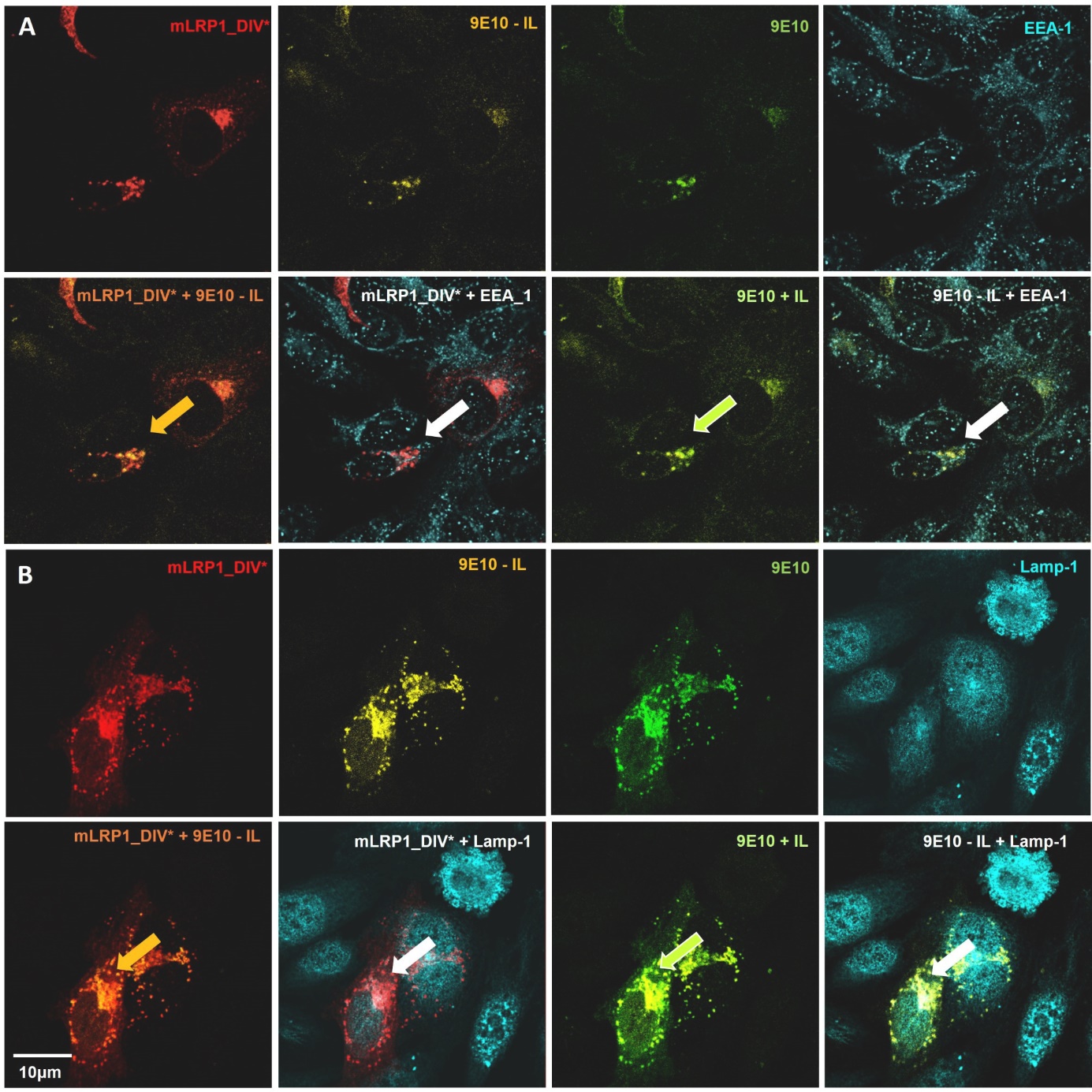


Figure S9: Subcellular sorting of 9E10 - IL/mLRP1_DIV* complex in co-stainings with Lamp-1 and EEA-1 during transport across hcMEC/D3 cells.

Cells were transfected with mLRP1_DIV* and transport of 9E10 - IL was performed 72h post transfection. Representative confocal images of mLRP1_DIV*, liposomes and 9E10 co-localization with intracellular markers (A) EEA-1 and (B) Lamp-1 2h after incubation with the liposomes. Cells were washed with acidic PBS, fixed with 4% PFA, permeabilized and stained for mLRP1_DIV*, 9E10 and corresponding intracellular structure (IS). Images were taken with the LSM710 confocal laser scanning microscope using a laser at a wavelength of (mLRP1_DIV*) 647nm, (rhodamine) 540nm, (9E10) 350nm and (IS) 488nm. mLRP1_DIV* is depicted in red, 9E10 - IL in yellow, 9E10 in green and (IS) in cyan. Co-localizations were investigated by merging two channels. (orange) mLRP1_DIV* + liposomes, (white) mLRP1_DIV* + corresponding IS, (light green) liposomes + 9E10 and (green/white) liposomes + corresponding IS. Scale bar = 10 µm.


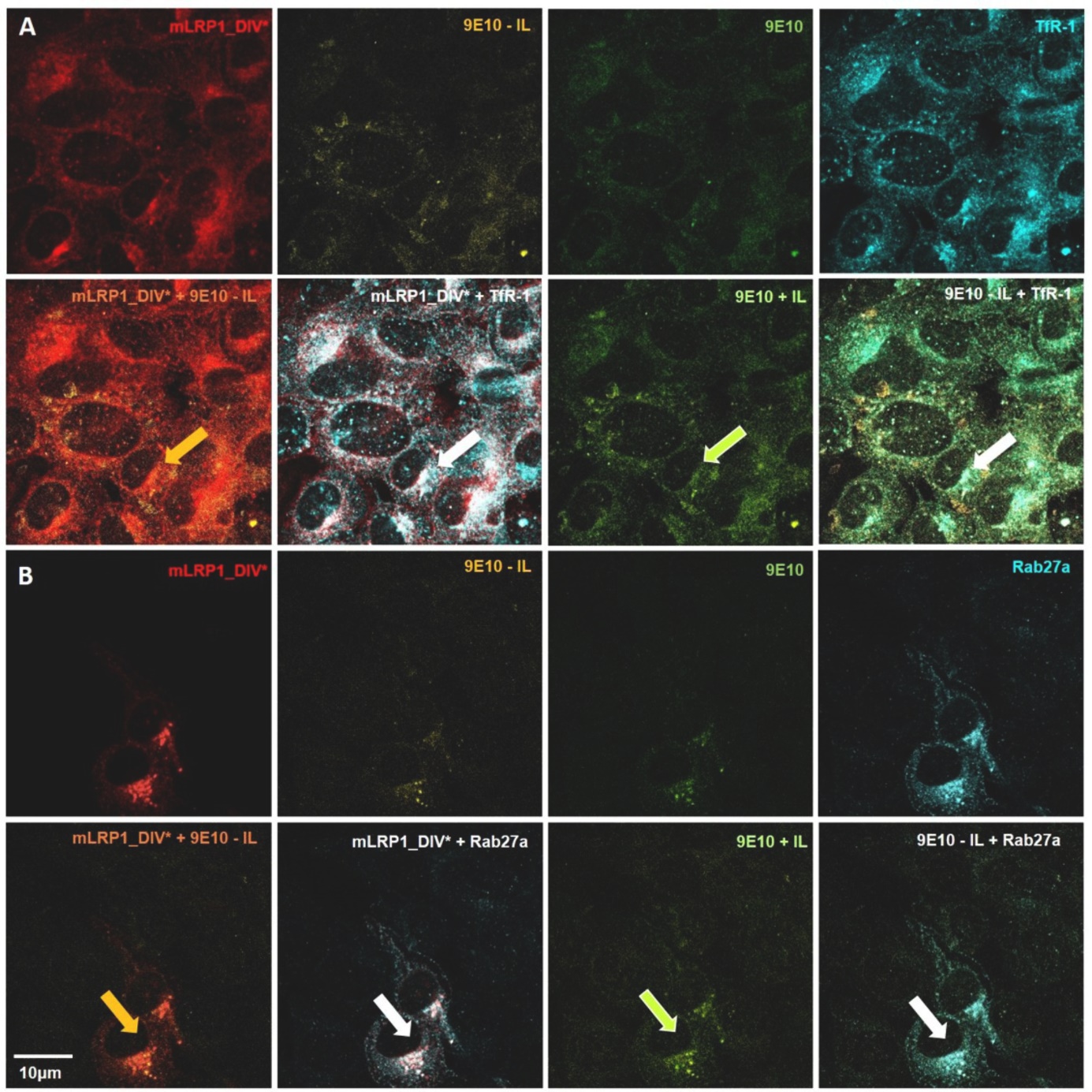


Figure S10: Subcellular sorting of 9E10 - IL/mLRP1_DIV* complex in co-stainings with TfR-1 and Rab27a during transport across hcMEC/D3 cells.

Cells were transfected with mLRP1_DIV* and transport of 9E10 - IL was performed 72h post transfection. Representative confocal images of mLRP1_DIV*, liposomes and 9E10 co-localization with intracellular markers (A) TfR-1 and (B) Rab27a 2h after incubation with the liposomes. Cells were washed with acidic PBS, fixed with 4% PFA, permeabilized and stained for mLRP1_DIV*, 9E10 and corresponding intracellular structure (IS). Images were taken with the LSM710 confocal laser scanning microscope using a laser at a wavelength of (mLRP1_DIV*) 647nm, (rhodamine) 540nm, (9E10) 350nm and (IS) 488nm. mLRP1_DIV* is depicted in red, 9E10 - IL in yellow, 9E10 in green and (IS) in cyan. Co-localizations were investigated by merging two channels. (orange) mLRP1_DIV* + liposomes, (white) mLRP1_DIV* + corresponding IS, (light green) liposomes + 9E10 and (green/white) liposomes + corresponding IS. Scale bar = 10 µm.

Regarding the vesicular transport route of unmodified liposomes, only a partial co-localization with mLRP1_DIV* has been observed (Figure S11 – S13). Additionally, both mLRP1_DIV* and unmodified liposomes are partially located within Clathrin, Lamp-1, TfR-1 and Rab27a positive structures, while no co-compartmentalization of both with the early endosome could be observed. While mLRP1_DIV* seems to co-localize with Caveolin-1, no co-localization of liposomes with this structure could be observed (Figure S11 and S13).


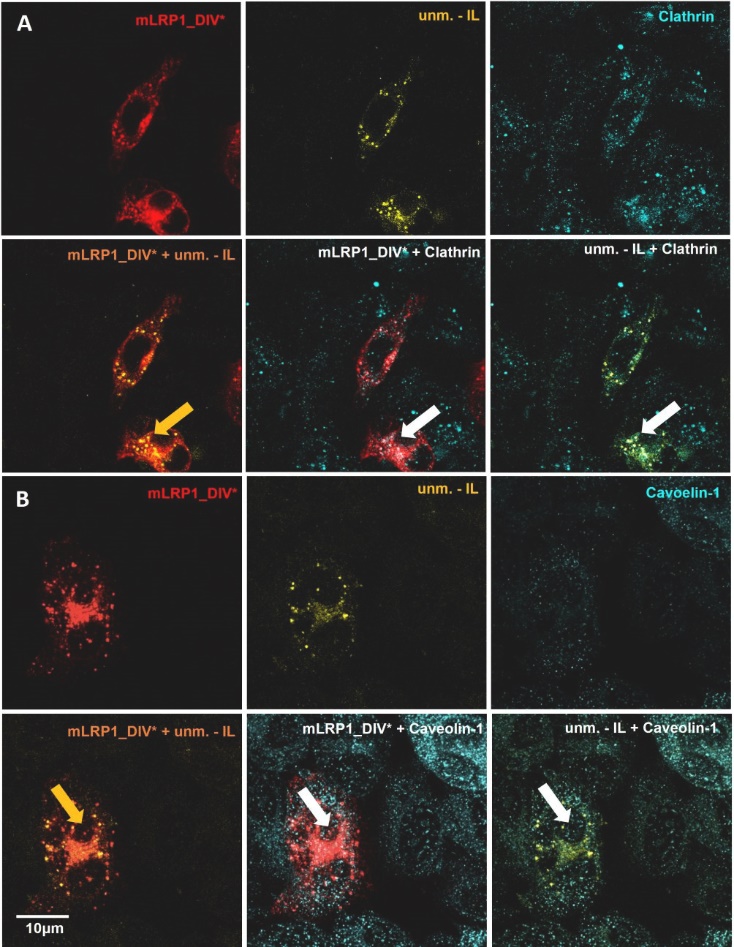


Figure S11: Subcellular sorting of unm. - IL and mLRP1_DIV* in co-stainings with Clathrin and Caveolin-1 during transport across hcMEC/D3 cells.

Cells were transfected with mLRP1_DIV* and transport of unm. - IL was performed 72h post transfection. Representative confocal images of mLRP1_DIV* and liposomes co-localization with intracellular markers (A) Clathrin and (B) Caveolin-1 2h after incubation with the liposomes. Cells were washed with acidic PBS, fixed with 4% PFA, permeabilized and stained for mLRP1_DIV* and corresponding intracellular structure (IS). Images were taken with the LSM710 confocal laser scanning microscope using a laser at a wavelength of (mLRP1_DIV*) 647nm, (rhodamine) 540nm and (IS) 488nm. mLRP1_DIV* is depicted in red, unm. - IL in yellow and IS in cyan. Co-localizations were investigated by merging two channels. (orange) mLRP1_DIV* + liposomes, (white) mLRP1_DIV* + corresponding IS and (white) liposomes + corresponding IS. Scale bar = 10 µm.


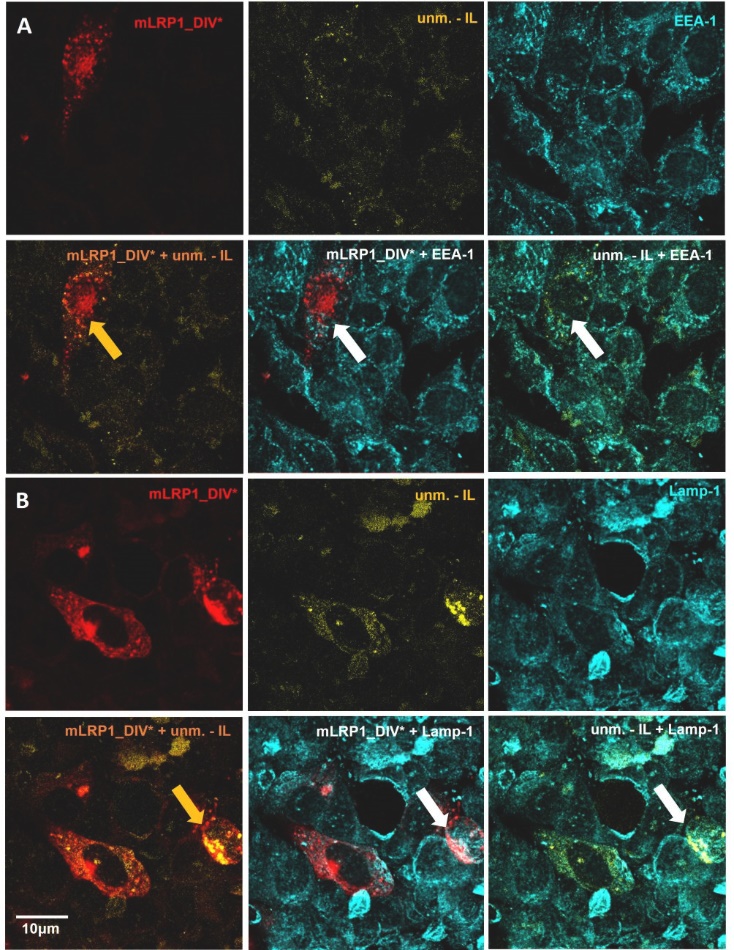


Figure S12: Subcellular sorting of unm. - IL and mLRP1_DIV* in co-stainings with EEA-1 and
Lamp-1 during transport across hcMEC/D3 cells.

Cells were transfected with mLRP1_DIV* and transport of unm. - IL was performed 72h post transfection. Representative confocal images of mLRP1_DIV* and liposomes co-localization with intracellular markers (A) EEA-1 and (B) Lamp-1 2h after incubation with the liposomes. Cells were washed with acidic PBS, fixed with 4% PFA, permeabilized and stained for mLRP1_DIV* and corresponding intracellular structure (IS). Images were taken with the LSM710 confocal laser scanning microscope using a laser at a wavelength of (mLRP1_DIV*) 647nm, (rhodamine) 540nm and (IS) 488nm. mLRP1_DIV* is depicted in red, unm. - IL in yellow and IS in cyan. Co-localizations were investigated by merging two channels. (orange) mLRP1_DIV* + liposomes, (white) mLRP1_DIV* + corresponding IS and (white) liposomes + corresponding IS. Scale bar = 10 µm.


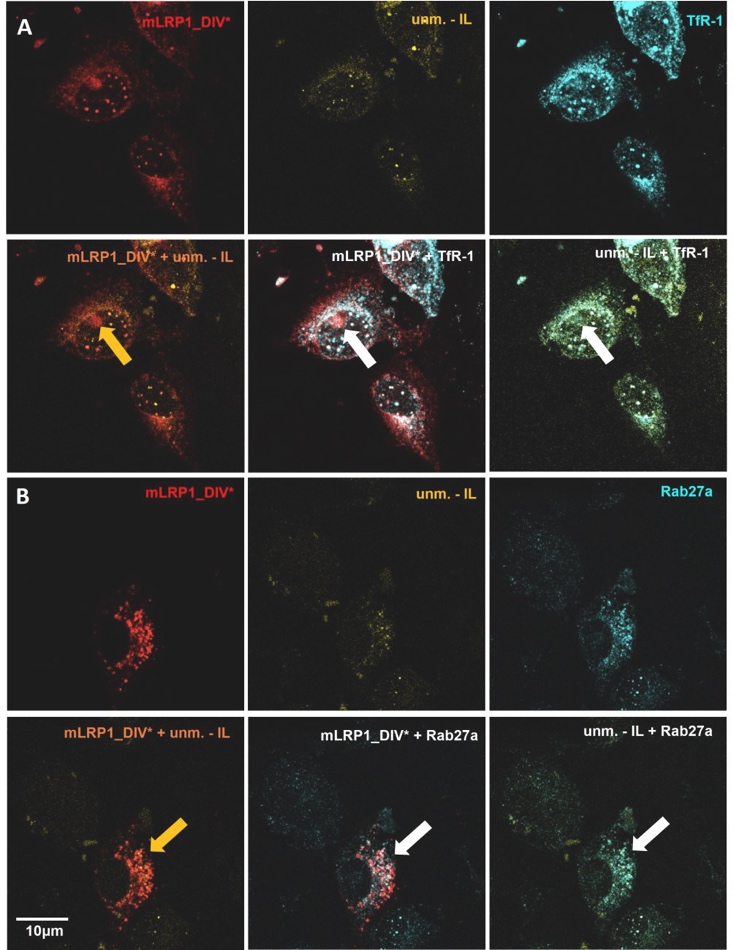


Figure S13: Subcellular sorting of unm. - IL and mLRP1_DIV* in co-stainings with TfR-1 and Rab27a during transport across hcMEC/D3 cells.

Cells were transfected with mLRP1_DIV* and transport of unm. - IL was performed 72h post transfection. Representative confocal images of mLRP1_DIV* and liposomes co-localization with intracellular markers (A) TfR-1 and (B) Rab27a 2h after incubation with the liposomes. Cells were washed with acidic PBS, fixed with 4% PFA, permeabilized and stained for mLRP1_DIV* and corresponding intracellular structure (IS). Images were taken with the LSM710 confocal laser scanning microscope using a laser at a wavelength of (mLRP1_DIV*) 647nm, (rhodamine) 540nm and (IS) 488nm. mLRP1_DIV* is depicted in red, unm. - IL in yellow and IS in cyan. Co-localizations were investigated by merging two channels. (orange) mLRP1_DIV* + liposomes, (white) mLRP1_DIV* + corresponding IS and (white) liposomes + corresponding IS. Scale bar = 10 µm.

***Liposomal BB25 modulated Aß species formation in PS70 cells***

Previous studies reported the acidic γ-secretase modulator (GSM) BB25, as a promising substrate in terms of modulating γ-secretase activity in the treatment of AD. Following treatment of CHO cells with stable co-expression of human APP751 and PSEN1 (PS70), BB25 displayed the typical characteristics of a GSM, including dose-dependent increase in Aß_38_ and decrease in Aß_42_ levels [3]. Here, the biological activity of liposomal BB25 (BB25 - 9E10 - IL) was first investigated using PS70 cells. Cells were treated with 1µM free BB25, liposomal BB25 or unloaded liposomes (unloaded 9E10 - IL) as control. The administered concentration of the liposomes was adjusted to the free BB25 to compare the effects of the liposomal and the free drug. Following a 48h incubation, tissue culture supernatants were obtained and an Aβ specific ELISA was used to quantify the levels of Aβ_38_ and Aβ_42_. Thereby, treatment of PS70 cells with 1µM free BB25 resulted in a 1.44-fold increase of Aß_38_ and a decrease of Aß_42_ of 78.5% compared to control (p = 0.0001 / p < 0.0001). Notably, liposomal BB25 exhibited the same biological activity as free BB25. Treatment with BB25 – 9E10 - IL resulted in a 1.34-fold increase in Aß_38_ as well as a decrease of Aß_42_ of 89.2% compared to control (p = 0.0007 / p < 0.0001). Additionally, no significant differences could be observed following a treatment with free BB25 compared to liposomal BB25 (p = 0.2576 / p = 0.3392) (Figure S14).


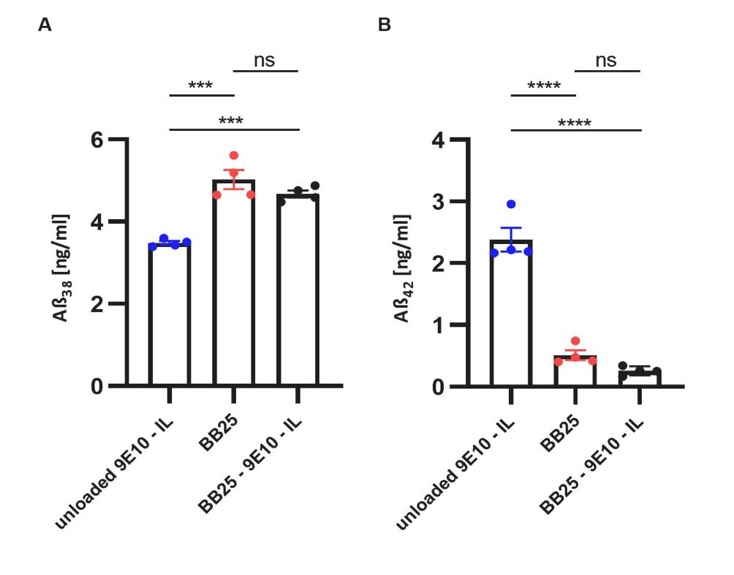


**Figure S14: Aß protein levels in PS70 cells changed after treatment with the liposomal γ-secretase modulator BB25.**

PS70 cells overexpressing human APP751 and Presenilin 1 were treated with 1µM BB25, liposomal BB25 or unloaded liposomes (Ctrl.) for 48h. The administered concentration of the liposomes was adjusted to the free BB25. Levels of (A) Aβ_38_ and (B) Aβ_42_ were measured by an Aβ species specific ELISA. Data represent the mean ± SEM of four individual replicates from n = 2 independent experiments. One-way ANOVA followed by Tukey’s multiple comparison test was used for statistical analysis**.**

***Liposomal BB25 did not interfere with the integrity of the endothelial barrier***

Since the liposomal γ-secretase modulator BB25 should be used for transport across the BBB, the impact of liposomal BB25 and immunoliposomes on the *in vitro* BBB’s integrity was investigated using TEER, a parameter reflecting the integrity of the monolayer, as well as paracellular diffusion by FITC-Dextran (3-4 kDa) (Figure S15). Therefore, formed and stimulated bEnd.3 mLRP1_DIV* cell monolayer was treated with 10 μM BB25, liposomal BB25, unloaded 9E10 functionalized liposomes or DMSO control for 48h. As a result, neither free BB25, nor liposomal BB25 or unloaded liposomes did interfere with the development of the TEER compared to control (Figure S15 A). Additionally, paracellular leakage of treated endothelial cells has been assed using FITC-Dextran (3-4 kDa), a carbohydrate with no known channel or receptor. Paracellular leakage of FITC-Dextran averaged out at 0.85% (Control),
0.79% (free BB25), 0.79% (BB25 – 9E10 - IL) or 0.81% (unloaded 9E10 - IL) 24h after application
(Figure S15 B). Thus, no changes in the endothelial barrier could be detected after treatment with one of the substances. All things considered, the endothelial cells' integrity and vitality were unaffected by free BB25, liposomal BB25 or unloaded liposomes, ruling out any potential harmful impact.


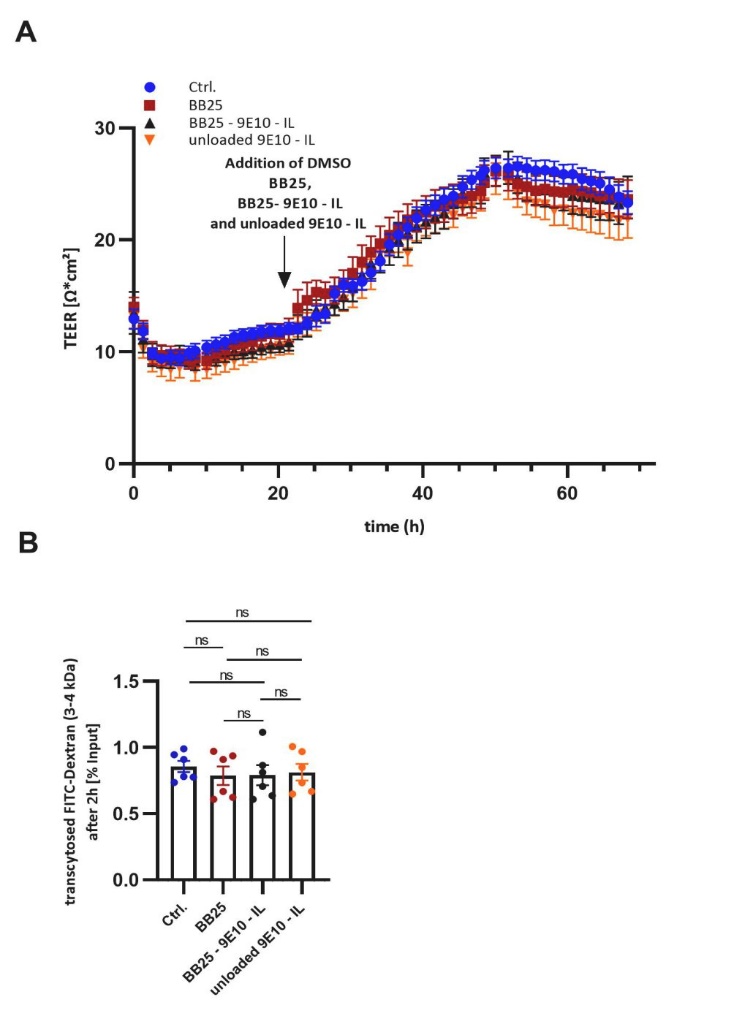


Figure S15: Barrier properties of bEnd.3 cells did not change after treatment with BB25, BB25 – 9E10 – IL or unloaded 9E10 – IL.

(A) bEnd.3 cells were transfected with mLRP1_DIV* and cultivated on cell culture inserts. When cells were post-confluent (CCI < 1 µF/cm^2^), luminal medium was supplemented with 10µM BB25, BB25 – 9E10 - IL, 9E10 - IL or DMSO (Ctrl.) and TEER was measured every hour by impedance spectroscopy over 48h. (B) Paracellular leakage of FITC-Dextran (3-4 kDa) 24h after addition of BB25, BB25 – 9E10 - IL, 9E10 - IL or DMSO (Ctrl.) for 24h. Data represent the mean ± SEM of six individual replicates from n = 3 independent experiments. One-way ANOVA followed by Tukey’s multiple comparison test was used for statistical analysis.

***Liposomal BB25 modulated Aß species formation in PS70 cells after transport across bEnd.3 in a co-culture model***

An application of BB25 – 9E10 – IL on bEnds.3 mLRP1_DIV* resulted in a 1.79-fold increase of Aß_38_ as well as a decrease of Aß_42_ of 76.2% compared to an application on bEnd.3 pLBCX cells (p < 0.0001,
p < 0.0001) (Figure S16).


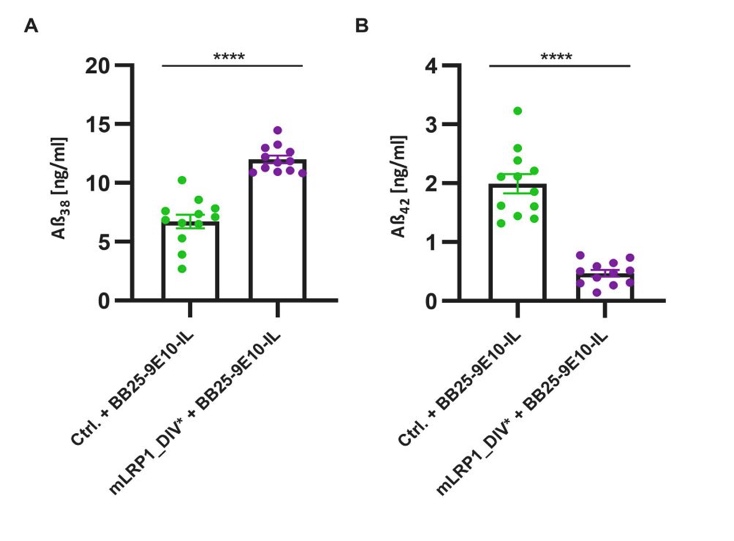


Figure S16: Aß levels changed after mLRP1_DIV* mediated transport of liposomal BB25 across bEnd.3 cells.

Transport of 10µM BB25 – 9E10 – IL across post-confluent bEnd.3 mLRP1_DIV* or pLBCX cells for 2h. Levels of (A) Aβ_38_ and (B) Aβ_42_ in abluminal cell culture supernatants were measured 48h post transport by an Aβ species specific ELISA. Data represent the mean ± SEM of twelve individual replicates from n = 3 independent experiments. Unpaired t-test was used for statistical analysis**.**

***Transfection controls***

To confirm a successful transient transfection of mLRP1_DIV* in hcMEC/D3 and bEnd.3 cells in internalization (Figure S6A and B) and transcytosis experiments (Figure 4 and 7), remaining cells during the experiments were lysed and analyzed for the expression of mLRP1_DIV* using SDS-PAGE and Western Blot analysis. All cells used, showed an expression of the mLRP1_DIV* construct and confirmed a successful transient transfection during the experiments. Thereby, two bands became visible, one at approximately 100 kDa and one at approximately 125 kDa, corresponding to the immature ER- protein (100 kDa) and the mature glycosylated transmembrane protein (125 kDa).


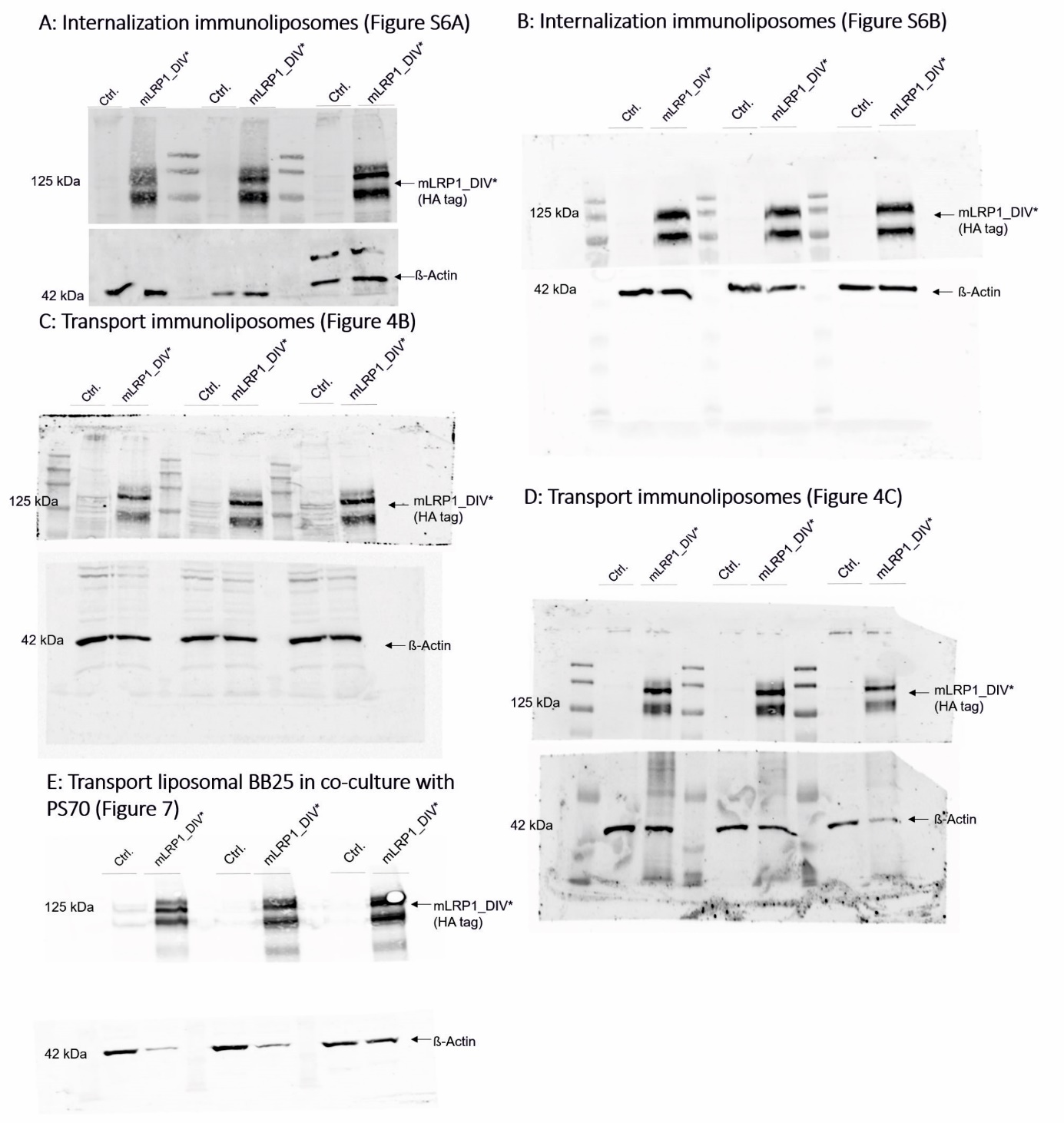


Figure S17: Transfection controls for internalization and transcytosis experiments with immunoliposomes.

Representative immunoblotting for protein levels in cell lysates of hcMEC/D3 or bEnd.3 cells. Cells were transiently transfected with mLRP1_DIV* or control and used 72h post transfection for internalization and transcytosis experiments. At the same time, cells were partially lysed and analyzed for the expression of mLRP1_DIV*. (A and B) internalization of immunoliposomes in hcMEC/D3, (C and D) transcytosis of immunoliposomes in hcMEC/D3 and (E) transport of BB25, liposomal BB25 or liposomes in bEnd.3 cells in co-culture with PS70 cells. Every blot represents one experimental setup with three biological replicates each.

***Transport of 9E10 using cytoplasmic inhibitors***

To further explore the mechanism by which mLRP1_DIV* mediates the entry of antibodies into the cell as well as the vesicular pathway of the mLRP1_DIV*/antibody complex, a transcytosis of 9E10 across bEnd.3 mLRP1_DIV* cells was performed with or without the lysosomal inhibitor Bafilomycin. Formed and stimulated bEnd.3 mLRP1_DIV* cell monolayer was treated with or without 10 nM Bafilomycin. Transcytosis of 9E10 was performed after 24h. Protein levels of 9E10 were measured 1h after transport in the abluminal medium using fluorescence spectroscopy. Thereby, no significant differences could be observed in the transcytosis of 9E10 upon Bafilomycin treatment compared to DMSO-treated cells
(p = 0.8773) (Figure S18). Results support our hypothesis of a straight transport of 9E10 from luminal to abluminal side mediated by mLRP1_DIV*, thereby avoiding lysosomal degradation.

***
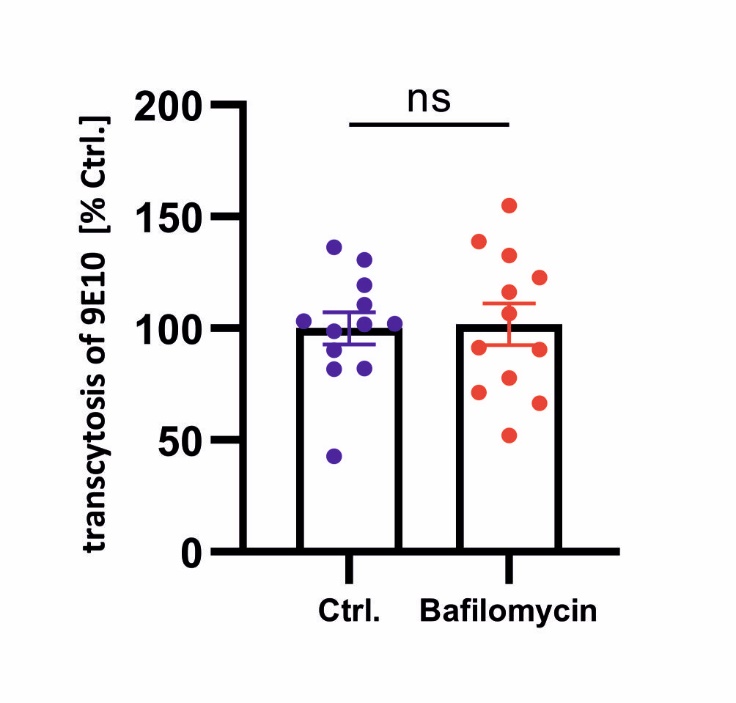
***

**Figure S18: Transcytosis of 9E10 using the lysosomal inhibitor Bafilomycin.**

Cells were transiently transfected with mLRP1_DIV*. At confluence, cells were incubated with 10nM Bafilomycin or DMSO control and transport of 2 µg/ml of anti-Myc Alexa Fluor^TM^ 555 was performed after 24h for 1h. Amount of transcytosed Alexa Fluor^TM^ 9E10 was analyzed using fluorescence spectroscopy. Transcytosed amount of anti-Myc Alexa Fluor^TM^ 555 was calculated percentual to the input saved before the transport. Cells incubated with DMSO control were defined as 100% (Ctrl.). Data represent the mean ± SEM of twelve individual replicates of
n = 3 independent experiments. Unpaired t- test was used for statistical analysis.

***Endogenous LRP1 protein levels did not change upon mLRP1_DIV* expression***

The effect of the expression of mLRP1_DIV* on full length LRP1 expression was also investigated in bend.3 cells. Thereby, Western blot analysis revealed no visible changes of full-length LRP1 protein levels in mLRP1_DIV* transfected bEnd.3 cells compared to non-transfected cells (p = 0.9410)
(Figure S19).


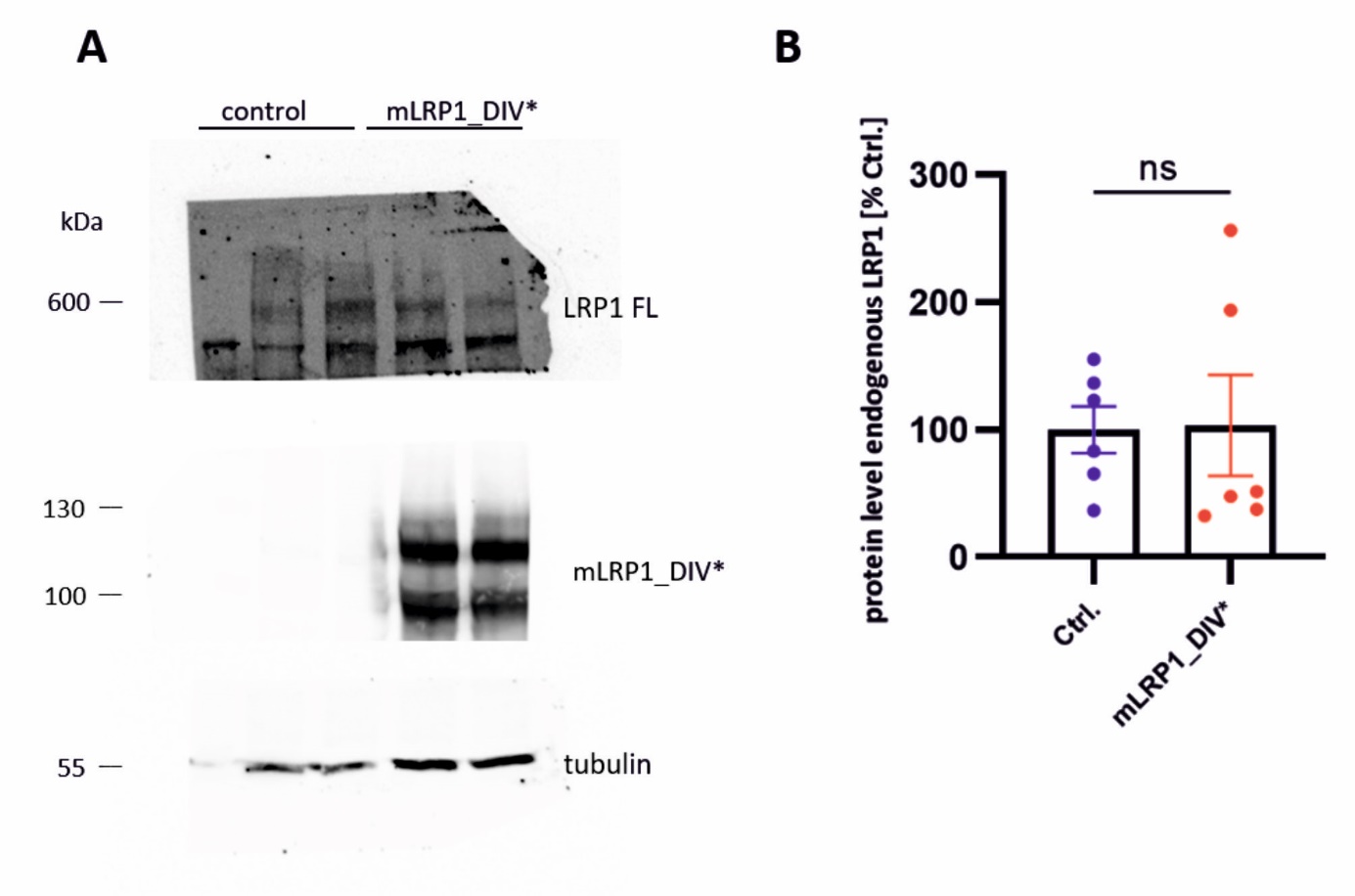


**Figure S19: Endogenous LRP1 levels in bEnd.3 mLRP1_DIV* vs. bEnd.3 wt cells.**

(A) Representative immunoblotting for protein levels in cell lysates of bEnd.3 cells 72h post transfection with mLRP1_DIV* compared to non-transfected cells. (B) Native LRP1 full-length protein levels were quantified by densitometric analysis after immunoblotting and normalized to α-tubulin. All data were normalized to the non-transfected control cells (Ctrl.). Data represent individual values and mean ± SEM of six individual replicates from
n = 3 independent experiments. Unpaired t-test was used for statistical analysis.

***AAV transduction in HEK293T cells***

After AAV(BR1)mLRP1_DIV* production, purification and determination of the titer, AAV infectivity was tested *in vitro* prior to *in vivo* administration in 5xFAD mice. For this purpose, HEK293T cells were transduced with AAV(BR1)mLRP1_DIV* and the efficiency of infection was determined by Western blot analysis. As shown in Figure S20, HEK293T were successfully transduced with AAV(BR1)mLRP1_DIV*. A band corresponding to the 85 kDa subunit of LRP1 could be detected in both control and infected cells, whereas a band corresponding to the LRP1mini-receptor was detected only in the treated cells at the height of 120 kDa (Figure S20 B). Additionally, Myc-tag signal from mLRP1_DIV* could be detected in AAV(BR1)mLRP1_DIV* transduced cells at 120 kDa. Unspecific bands could be detected in all cells at 85 kDa and above 245 kDa (Figure S20 C). Similarly, HA-tag signal was detected only in transduced cells at the height of 120 and 85 kDa. Unspecific weak bands at the height of approximately 80 kDa could be detected in all the cells (Figure S20 D). For further experiments, we assumed an expression of mLRP1_DIV*, including the N-terminal Myc-tag and the C-terminal HA-tag.


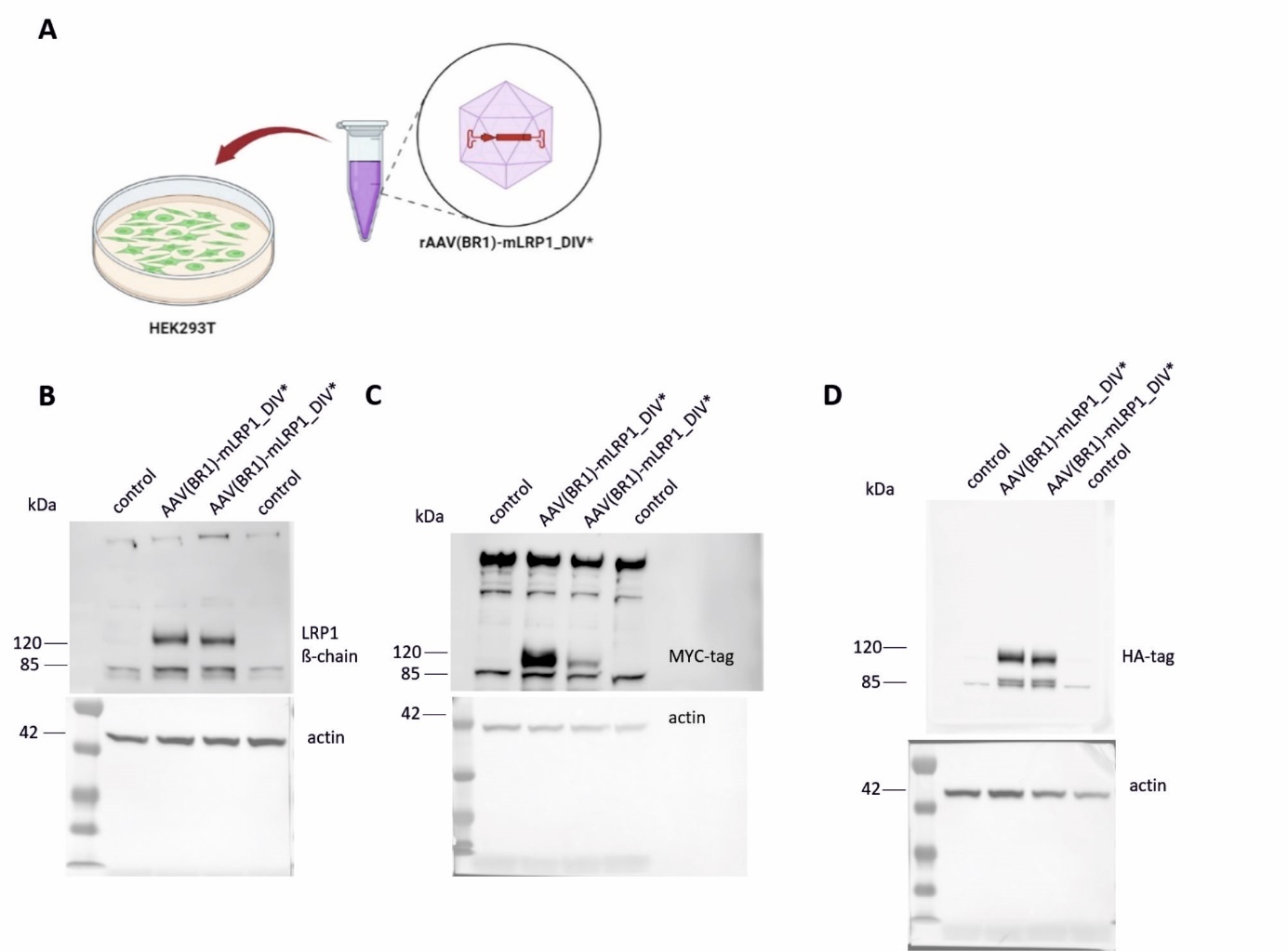


Figure S20: AAV(BR1)mLRP1_DIV* transduction in HEK293T cells.

HEK293T were transduced with 1x1010 AAV(BR1)mLRP1_DIV* genomic particles per well. mLRP1_DIV* expression was determined by Western blot analysis seven days post infection using antibodies against (B) LRP1 ß-chain (1704), (C) Myc-tag (9E10) and (D) HA-tag. Actin was used as a loading control.

References

1. Storck SE, Meister S, Nahrath J, Meißner JN, Schubert N, Di Spiezio A, et al. Endothelial LRP1 transports amyloid-β(1-42) across the blood-brain barrier. J Clin Invest. 2016;126:123–36. doi:10.1172/JCI81108.

2. Körbelin J, Dogbevia G, Michelfelder S, Ridder DA, Hunger A, Wenzel J, et al. A brain microvasculature endothelial cell-specific viral vector with the potential to treat neurovascular and neurological diseases. EMBO Mol Med. 2016;8:609–25. doi:10.15252/emmm.201506078.

3. Hahn S, Brüning T, Ness J, Czirr E, Baches S, Gijsen H, et al. Presenilin-1 but not amyloid precursor protein mutations present in mouse models of Alzheimer's disease attenuate the response of cultured cells to γ-secretase modulators regardless of their potency and structure. J Neurochem. 2011;116:385–95. doi:10.1111/j.1471-4159.2010.07118.x.
