# Supplementary material for: Truncated mini LRP1 transports cargo from luminal to basolateral side across the blood brain barrier
Source: Fluids Barriers CNS. 2024 Sep 17;21:74. doi: 10.1186/s12987-024-00573-1 (PMC11409491; doi:10.1186/s12987-024-00573-1)
Supplement: Supplementary file 8 — Supplementary Material 8 [file 12987_2024_573_MOESM8_ESM.pdf]

Sequence: pAAV CMV mLRP1_DIV.dna (Circular / 6501 bp)
Enzymes: Unique 6+ Cutters (43 of 666 total)
Features: 28 total
Primers: 3 total

Unique Cutters **Bold**

```
5' CCTGCAGGCAGCTGCGCGCTCGCTCGCTCACTGAGGCCGCCCGGGCAAAGCCCGGGCGTCGGGCGACCTTTGGTCGCCCGGCCTCAGTGA   90
3' GGACGTCCGTCGACGCGCGAGCGAGCGAGTGACTCCGGCGGGCCCGTTTCGGGCCCGCAGCCCGCTGGAAACCAGCGGGCCGGAGTCACT
```

AAV2 ITR

Acc65I **KpnI** **NheI**

```
GCGAGCGAGCGCGCAGAGAGGGAGTGGCCAACTCCATCACTAGGGGTTCCTGCGGCCGCATCGATAGGTACCGAGCTCTTACGCGTGcta   180
CGCTCGCTCGCGCGTCTCTCCCTCACCGGTTGAGGTAGTGATCCCCAAGGACGCCGGCGTAGCTATCCATGGCTCGAGAATGCGCACgat
```

AAV2 ITR | sequenced 1c1 and 1c3

deleted in sequencing

deleted in sequencing

BmtI **SpeI**

```
gcACGCGTTGACATTGATTATTGACTAGTTATTAATAGTAATCAATTACGGGGTCATTAGTTCATAGCCCATATATGGAGTTCCGCGTTA   270
cgTGCGCAACTGTAACTAATAACTGATCAATAATTATCATTAGTTAATGCCCCAGTAATCAAGTATCGGGTATATACCTCAAGGCGCAAT
```

sequenced 1c1 and 1c3

CMV

deleted in sequencing

CMV enhancer

```
CATAACTTACGGTAAATGGCCCGCCTGGCTGACCGCCCAACGACCCCCGCCCATTGACGTCAATAATGACGTATGTTCCCATAGTAACGC   360
GTATTGAATGCCATTTACCGGGCGGACCGACTGGCGGGTTGCTGGGGGCGGGTAACTGCAGTTATTACTGCATACAAGGGTATCATTGCG
```

sequenced 1c1 and 1c3

CMV

CMV enhancer

NdeI

```
CAATAGGGACTTTCCATTGACGTCAATGGGTGGAGTATTTACGGTAAACTGCCCACTTGGCAGTACATCAAGTGTATCATATGCCAAGTA   450
GTTATCCCTGAAAGGTAACTGCAGTTACCCACCTCATAAATGCCATTTGACGGGTGAACCGTCATGTAGTTCACATAGTATACGGTTCAT
```

sequenced 1c1 and 1c3

CMV

CMV enhancer

```
CGCCCCCTATTGACGTCAATGACGGTAAATGGCCCGCCTGGCATTATGCCCAGTACATGACCTTATGGGACTTTCCTACTTGGCAGTACA   540
GCGGGGGATAACTGCAGTTACTGCCATTTACCGGGCGGACCGTAATACGGGTCATGTACTGGAATACCCTGAAAGGATGAACCGTCATGT
```

sequenced 1c1 and 1c3

CMV

CMV enhancer

SnaBI

```
TCTACGTATTAGTCATCGCTATTACCATGGTGATGCGGTTTTGGCAGTACATCAATGGGCGTGGATAGCGGTTTGACTCACGGGGATTTC   630
AGATGCATAATCAGTAGCGATAATGGTACCACTACGCCAAAACCGTCATGTAGTTACCCGCACCTATCGCCAAACTGAGTGCCCCTAAAG
```

sequenced 1c1 and 1c3

CMV

CMV enhancer | CMV promoter

```
CAAGTCTCCACCCCATTGACGTCAATGGGAGTTTGTTTTGGAACCAAAATCAACGGGACTTTCCAAAATGTCGTAACAACTCCGCCCCAT   720
GTTCAGAGGTGGGGTAACTGCAGTTACCCTCAAACAAAACCTTGGTTTTAGTTGCCCTGAAAGGTTTTACAGCATTGTTGAGGCGGGGTA
```

sequenced 1c1 and 1c3

CMV

CMV promoter

G in sequencing

```
TGACGCAAATGGGCGGTAGGCGTGTACGGTGGGAGGTCTATATAAGCAGAGCTCTCCCTATCAGTGATAGAGATCTCCCTATCAGTGATA   810
ACTGCGTTTACCCGCCATCCGCACATGCCACCCTCCAGATATATTCGTCTCGAGAGGGATAGTCACTATCTCTAGAGGGATAGTCACTAT
```

sequenced 1c1 and 1c3

CMV

CMV promoter — tet operator — tet operator

SalI, SgrDI, AccI

```
GAGATCGTCGACGAGCTCGTTTAGTGAACCGTCAGATCGCCTGGAGACGCCATCCACGCTGTTTTGACCTCCATAGAAGACACCGGGACC   900
CTCTAGCAGCTGCTCGAGCAAATCACTTGGCAGTCTAGCGGACCTCTGCGGTAGGTGCGACAAAACTGGAGGTATCTTCTGTGGCCCTGG
```

sequenced 1c1 and 1c3

CMV

tet operator

BspEI, PmeI

```
GATCCAGCCTCCGGACTCTAGCGTTTAAACGCCACCAtgctgaccccgccgttgctcctgctgctgcccctgctctcagctctggtcgcg   990
CTAGGTCGGAGGCCTGAGATCGCAAATTTGCGGTGGTacgactggggcggcaacgaggacgacgacggggacgagagtcgagaccagcgc
```

CMV — Kozak sequence — LRP1mini

sequenced 1c1 and 1c3

BamHI

```
gcggctatcgacgcccctggaGAACAAAAACTTATTTCTGAAGAAGATCTGggatccacccactgcaaagacaagaaggagtttctgtgc   1080
cgccgatagctgcggggacctCTTGTTTTTGAATAAAGACTTCTTCTAGACcctaggtgggtgacgtttctgttcttcctcaaagacacg
```

LRP1mini

1 5 10

E Q K L I S E E D L G S T H C K D K K E F L C

added during cloning — Myc — (in frame with Myc)

sequenced 1c1 and 1c3

deleted? low quality peak in sequencing!

CTTCCTCAAAGACACG — LRP1mini seq rev

AfeI

```
cggaaccagcgctgcctctcctcctccctgcgctgcaacatgttcgatgactgcggggacggctctgacgaggaggactgcagcatcgac   1170
gccttggtcgcgacggagaggaggagggacgcgacgttgtacaagctactgacgcccctgccgagactgctcctcctgacgtcgtagctg
```

LRP1mini

R N Q R C L S S S L R C N M F D D C G D G S D E E D C S I D

(in frame with Myc)

GC GTGCACG — LRP1mini seq rev

FspAI

```
cccaagctgaccagctgcgccaccaatgccagcatctgtggggacgaggcacgctgcgtgcgcaccgagaaagcggcctactgtgcctgc   1260
gggttcgactggtcgacgcggtggttacggtcgtagacacccctgctccgtgcgacgcacgcgtggctctttcgccggatgacacggacg
```

LRP1mini

P K L T S C A T N A S I C G D E A R C V R T E K A A Y C A C

(in frame with Myc)

```
cgctcgggcttccacaccgtgcccggccagcccggatgccaagacatcaacgagtgcctgcgcttcggcacctgctcccagctctgcaac   1350
gcgagcccgaaggtgtggcacgggccggtcgggcctacggttctgtagttgctcacggacgcgaagccgtggacgagggtcgagacgttg
```

LRP1mini

R S G F H T V P G Q P G C Q D I N E C L R F G T C S Q L C N

(in frame with Myc)

SexAI*

aacaccaagggcggccacctctgcagctgcgctcggaacttcatgaagacgcacaacacctgcaaggccgaaggctctgagtaccaggtc 1440
ttgtggttcccgccggtggagacgtcgacgcgagccttgaagtacttctgcgtgttgtggacgttccggcttccgagactcatggtccag
LRP1mini
N T K G G H L C S C A R N F M K T H N T C K A E G S E Y Q V
(in frame with Myc)

BsrGI

ctgtacatcgctgatgacaatgagatccgcagcctgttccccggccaccccattcggcttacgagcaggcattccagggtgacgagagt 1530
gacatgtagcgactactgttactctaggcgtcggacaaggggccggtggggtaagccgaatgctcgtccgtaaggtcccactgctctca
LRP1mini
L Y I A D D N E I R S L F P G H P H S A Y E Q A F Q G D E S
(in frame with Myc)

gtccgcattgatgctatggatgtccatgtcaaggctggccgtgtctattggaccaactggcacacgggcaccatctcctaccgcagcctg 1620
caggcgtaactacgatacctacaggtacagttccgaccggcacagataacctggttgaccgtgtgcccgtggtagaggatggcgtcggac
LRP1mini
V R I D A M D V H V K A G R V Y W T N W H T G T I S Y R S L
(in frame with Myc)

SgrAI

ccacctgctgcgcctcctaccacttccaaccgccaccggcgacagattgaccggggtgtcacccacctcaacatttcagggctgaagatg 1710
ggtggacgacgcggaggatggtgaaggttggcggtggccgctgtctaactggccccacagtgggtggagttgtaaagtcccgacttctac
LRP1mini
P P A A P P T T S N R H R R Q I D R G V T H L N I S G L K M
(in frame with Myc)

cccagaggcatcgccatcgactgggtggccggaaacgtgtactggaccgactcgggccgagatgtgattgaggtggcgcagatgaagggc 1800
gggtctccgtagcggtagctgacccaccggcctttgcacatgacctggctgagcccggctctacactaactccaccgcgtctacttcccg
LRP1mini
P R G I A I D W V A G N V Y W T D S G R D V I E V A Q M K G
(in frame with Myc)

gagaaccgcaagacgctcatctcgggcatgattgacgagccccacgccattgtggtggacccactgaggggggaccatgtactggtcagac 1890
ctcttggcgttctgcgagtagagcccgtactaactgctcggggtgcggtaacaccacctgggtgactcccccctggtacatgaccagtctg
LRP1mini
E N R K T L I S G M I D E P H A I V V D P L R G T M Y W S D
(in frame with Myc)

tggggcaaccaccccaagattgagacggcagcgatggatgggacgcttcgggagacactggtgcaggacaacattcagtggcccacaggc 1980
accccgttggtggggttctaactctgccgtcgctacctaccctgcgaagccctctgtgaccacgtcctgttgtaagtcaccgggtgtccg
LRP1mini
W G N H P K I E T A A M D G T L R E T L V Q D N I Q W P T G
(in frame with Myc)

HindIII

ctggccgtggattatcacaatgagcggctgtactgggcagacgccaagctttcagtcatcggcagcatccggctcaatggcacggacccc 2070
gaccggcacctaatagtgttactcgccgacatgacccgtctgcggttcgaaagtcagtagccgtcgtaggccgagttaccgtgcctgggg
LRP1mini
L A V D Y H N E R L Y W A D A K L S V I G S I R L N G T D P
(in frame with Myc)

attgtggctgctgacagcaaacgaggcctaagtcaccccttcagcatcgacgtctttgaggattacatctatggtgtcacctacatcaat 2160
taacaccgacgactgtcgtttgctccggattcagtggggaagtcgtagctgcagaaactcctaatgtagataccacagtggatgtagtta
LRP1mini
I V A A D S K R G L S H P F S I D V F E D Y I Y G V T Y I N
(in frame with Myc)

PflFI
Tth111I

aatcgtgtcttcaagatccataagtttggccacagccccttggtcaacctgacaggggcctgagccacgcctctgacgtggtcctttac 2250
ttagcacagaagttctaggtattcaaaccggtgtcggggaaccagttggactgtccccggactcggtgcggagactgcaccaggaaatg
LRP1mini
N R V F K I H K F G H S P L V N L T G G L S H A S D V V L Y
(in frame with Myc)

BstAPI BlpI

catcagcacaagcagcccgaagtgaccaacccatgtgaccgcaagaaatgcgagtggctctgcctgctgagccccagtggcctgtctgc 2340
gtagtcgtgttcgtcgggcttcactggttgggtacactggcgttctttacgctcaccgagacggacgactcggggtcacccggacagacg
LRP1mini
H Q H K Q P E V T N P C D R K K C E W L C L L S P S G P V C
(in frame with Myc)

LRPCT forward Miriam
ttcgggatcc aagcggctggacaac

AleI

acctgtcccaatgggaagcggctggacaacggcacatgcgtgcctgtgccctctccaacgcccccccccagatgctccccggcctggaacc 2430
tggacagggttacccttcgccgacctgttgccgtgtacgcacggacacgggagaggttgcggggggggtctacgaggggccggaccttgg
LRP1mini
T C P N G K R L D N G T C V P V P S P T P P P D A P R P G T
(in frame with Myc)
sequenced in 1c1 and 1c3

tgtaacctgcagtgcttcaacggtggcagctgtttcctcaatgcacggaggcagcccaagtgccgctgccaaccccgctacacgggtgac 2520
acattggacgtcacgaagttgccaccgtcgacaaaggagttacgtgcctccgtcgggttcacggcgacggttggggcgatgtgcccactg
LRP1mini
C N L Q C F N G G S C F L N A R R Q P K C R C Q P R Y T G D
(in frame with Myc)
sequenced in 1c1 and 1c3

SphI PmlI

aagtgtgaactggaccagtgctgggagcactgtcgcaatgggggcacctgtgctgcctccccctctggcatgcccacgtgccggtgcccc 2610
ttcacacttgacctggtcacgaccctcgtgacagcgttacccccgtggacacgacggagggggagaccgtacgggtgcacggccacgggg
LRP1mini
K C E L D Q C W E H C R N G G T C A A S P S G M P T C R C P
(in frame with Myc)
sequenced in 1c1 and 1c3

acgggcttcacgggccccaaatgcacccagcaggtgtgtgcgggctactgtgccaacaacagcacctgcactgtcaaccagggcaaccag 2700
tgcccgaagtgcccggggtttacgtgggtcgtccacacacgcccgatgacacggttgttgtcgtggacgtgacagttggtcccgttggtc
LRP1mini
T G F T G P K C T Q Q V C A G Y C A N N S T C T V N Q G N Q
(in frame with Myc)
sequenced in 1c1 and 1c3

ccccagtgccgatgcctacccggcttcctgggcgaccgctgccagtaccggcagtgctctggctactgtgagaactttggcacatgccag 2790
ggggtcacggctacggatgggccgaaggacccgctggcgacggtcatggccgtcacgagaccgatgacactcttgaaaccgtgtacggtc
LRP1mini
P Q C R C L P G F L G D R C Q Y R Q C S G Y C E N F G T C Q
(in frame with Myc)
sequenced in 1c1 and 1c3

atggctgctgatggctcccgacaatgccgctgcactgcctactttgagggatcgaggtgtgaggtgaacaagtgcagccgctgtctcgaa 2880
taccgacgactaccgagggctgttacggcgacgtgacggatgaaactccctagctccacactccacttgttcacgtcggcgacagagctt
LRP1mini
M A A D G S R Q C R C T A Y F E G S R C E V N K C S R C L E
(in frame with Myc)
sequenced in 1c1 and 1c3

PshAI
ggggcctgtgtggtcaacaagcagagtggggatgtcacctgcaactgcacggatggccgggtggcccccagctgtctgacctgcgtcggc 2970
ccccggacacaccagttgttcgtctcacccctacagtggacgttgacgtgcctaccggcccaccgggggtcgacagactggacgcagccg
LRP1mini
G A C V V N K Q S G D V T C N C T D G R V A P S C L T C V G
(in frame with Myc)
sequenced in 1c1 and 1c3

cactgcagcaatggcggctcctgtaccatgaacagcaaaatgatgcctgagtgccagtgcccaccccacatgacagggccccggtgtgag 3060
gtgacgtcgttaccgccgaggacatggtacttgtcgttttactacggactcacggtcacgggtggggtgtactgtcccggggccacactc
LRP1mini
H C S N G G S C T M N S K M M P E C Q C P P H M T G P R C E
(in frame with Myc)
sequenced in 1c1 and 1c3

gagcacgtcttcagccagcagcagccaggacatatagcctccatcctaatccctctgctgttgctgctgctgctggttctggtggccgga 3150
ctcgtgcagaagtcggtcgtcgtcggtcctgtatatcggaggtaggattagggagacgacaacgacgacgacgaccaagaccaccggcct
LRP1mini
E H V F S Q Q Q P G H I A S I L I P L L L L L L L V L V A G
(in frame with Myc)
sequenced in 1c1 and 1c3

LRP1CT seq fwd
ctggtataagcggcgagtc
gtggtattctggtataagcggcgagtccaagggggctaagggcttccagcaccaacggatgaccaacggggccatgaacgtggagattgga 3240
caccataagaccatattcgccgctcaggttccccgattcccgaaggtcgtggttgcctactggttgccccggtacttgcacctctaacct
LRP1mini
V V F W Y K R R V Q G A K G F Q H Q R M T N G A M N V E I G
(in frame with Myc)
sequenced in 1c1 and 1c3 | sequenced in 1c1 and 1c3

aaccccacctacaagatgtacgaaggcggagagcctgatgatgtgggaggcctactggacgctgactttgccctggaccctgacaagccc 3330
ttggggtggatgttctacatgcttccgcctctcggactactacaccctccggatgacctgcgactgaaacgggacctgggactgttcggg
LRP1mini
N P T Y K M Y E G G E P D D V G G L L D A D F A L D P D K P
(in frame with Myc)
sequenced in 1c1 and 1c3

accaacttcaccaaccccgtgtatgccacactctacatgggggccatggcagtcgccactccctggccagcacggacgagaagcgagaa 3420
tggttgaagtggttggggcacatacggtgtgagatgtacccccggtaccgtcagcggtgagggaccggtcgtgcctgctcttcgctctt
LRP1mini
T N F T N P V Y A T L Y M G G H G S R H S L A S T D E K R E
(in frame with Myc)
sequenced in 1c1 and 1c3

Bsu36I KflI XbaI

ctcctgggccggggccctgaggacgagataggggaccccttggcaTACCCATACGATGTTCCAGATTACGCTTAGTCTAGAGTCGGGGCG 3510
gaggacccggccccgggactcctgctctatcccctggggaaccgtATGGGTATGCTACAAGGTCTAATGCGAATCAGATCTCAGCCCCGC

LRP1mini

additional 8 bases shown in sequencing

L L G R G P E D E I G D P L A Y P Y D V P D Y A *

(in frame with Myc)

HA

sequenced in 1c1 and 1c3

FseI

GCCGGCCGCTTCGAGCAGACATGATAAGATACATTGATGAGTTTGGACAAACCACAACTAGAATGCAGTGAAAAAAATGCTTTATTTGTG 3600
CGGCCGGCGAAGCTCGTCTGTACTATTCTATGTAACTACTCAAACCTGTTTGGTGTTGATCTTACGTCACTTTTTTTACGAAATAAACAC

sequenced in 1c1 and 1c3

SV40 poly(A) signal

HpaI MfeI

AAATTTGTGATGCTATTGCTTTATTTGTAACCATTATAAGCTGCAATAAACAAGTTAACAACAACAATTGCATTCATTTTATGTTTCAGG 3690
TTTAAACACTACGATAACGAAATAAACATTGGTAATATTCGACGTTATTTGTTCAATTGTTGTTGTTAACGTAAGTAAAATACAAAGTCC

sequenced in 1c1 and 1c3

SV40 poly(A) signal

TTCAGGGGGAGGTGTGGGAGGTTTTTTAAAGCAAGTAAAACCTCTACAAATGTGGTAAAATCGATGCGGCCGCAGGAACCCCTAGTGATG 3780
AAGTCCCCCTCCACACCCTCCAAAAAATTTCGTTCATTTTGGAGATGTTTACACCATTTTAGCTACGCCGGCGTCCTTGGGGATCACTAC

sequenced in 1c1 and 1c3

deleted in 1c1 and 1c3 sequencing

AAV2 ITR

GAGTTGGCCACTCCCTCTCTGCGCGCTCGCTCGCTCACTGAGGCCGGGCGACCAAAGGTCGCCCGACGCCCGGGCTTTGCCCGGGCGGCC 3870
CTCAACCGGTGAGGGAGAGACGCGCGAGCGAGCGAGTGACTCCGGCCCGCTGGTTTCCAGCGGGCTGCGGGCCCGAAACGGGCCCGCCGG

sequenced in 1c1 and 1c3

AAV2 ITR

KasI NarI SfoI PluTI

TCAGTGAGCGAGCGAGCGCGCAGCTGCCTGCAGGGGCGCCTGATGCGGTATTTTCTCCTTACGCATCTGTGCGGTATTTCACACCGCATA 3960
AGTCACTCGCTCGCTCGCGCGTCGACGGACGTCCCCGCGGACTACGCCATAAAAGAGGAATGCGTAGACACGCCATAAAGTGTGGCGTAT

AAV2 ITR

CGTCAAAGCAACCATAGTACGCGCCCTGTAGCGGCGCATTAAGCGCGGCGGGTGTGGTGGTTACGCGCAGCGTGACCGCTACACTTGCCA 4050
GCAGTTTCGTTGGTATCATGCGCGGGACATCGCCGCGTAATTCGCGCCGCCCACACCACCAATGCGCGTCGCACTGGCGATGTGAACGGT

f1 ori

GCGCCCTAGCGCCCGCTCCTTTCGCTTTCTTCCCTTCCTTTCTCGCCACGTTCGCCGGCTTTCCCCGTCAAGCTCTAAATCGGGGGCTCC 4140
CGCGGGATCGCGGGCGAGGAAAGCGAAAGAAGGGAAGGAAAGAGCGGTGCAAGCGGCCGAAAGGGGCAGTTCGAGATTTAGCCCCCGAGG

f1 ori

CTTTAGGGTTCCGATTTAGTGCTTTACGGCACCTCGACCCCAAAAAACTTGATTTGGGTGATGGTTCACGTAGTGGGCCATCGCCCTGAT 4230
GAAATCCCAAGGCTAAATCACGAAATGCCGTGGAGCTGGGGTTTTTTGAACTAAACCCACTACCAAGTGCATCACCCGGTAGCGGGACTA

f1 ori

AGACGGTTTTTCGCCCTTTGACGTTGGAGTCCACGTTCTTTAATAGTGGACTCTTGTTCCAAACTGGAACAACACTCAACCCTATCTCGG 4320
TCTGCCAAAAAGCGGGAAACTGCAACCTCAGGTGCAAGAAATTATCACCTGAGAACAAGGTTTGACCTTGTTGTGAGTTGGGATAGAGCC

f1 ori

```
GCTATTCTTTTGATTTATAAGGGATTTTGCCGATTTCGGCCTATTGGTTAAAAAATGAGCTGATTTAACAAAAATTTAACGCGAATTTTA   4410
CGATAAGAAAACTAAATATTCCCTAAAACGGCTAAAGCCGGATAACCAATTTTTTACTCGACTAAATTGTTTTTAAATTGCGCTTAAAAT
f1 ori

ACAAAATATTAACGTTTACAATTTTATGGTGCACTCTCAGTACAATCTGCTCTGATGCCGCATAGTTAAGCCAGCCCCGACACCCGCCAA   4500
TGTTTTATAATTGCAAATGTTAAAATACCACGTGAGAGTCATGTTAGACGAGACTACGGCGTATCAATTCGGTCGGGGCTGTGGGCGGTT
f1 ori

PfoI
CACCCGCTGACGCGCCCTGACGGGCTTGTCTGCTCCCGGCATCCGCTTACAGACAAGCTGTGACCGTCTCCGGGAGCTGCATGTGTCAGA   4590
GTGGGCGACTGCGCGGGACTGCCCGAACAGACGAGGGCCGTAGGCGAATGTCTGTTCGACACTGGCAGAGGCCCTCGACGTACACAGTCT

GGTTTTCACCGTCATCACCGAAACGCGCGAGACGAAAGGGCCTCGTGATACGCCTATTTTTATAGGTTAATGTCATGATAATAATGGTTT   4680
CCAAAAGTGGCAGTAGTGGCTTTGCGCGCTCTGCTTTCCCGGAGCACTATGCGGATAAAAATATCCAATTACAGTACTATTATTACCAAA

CTTAGACGTCAGGTGGCACTTTTCGGGGAAATGTGCGCGGAACCCCTATTTGTTTATTTTTCTAAATACATTCAAATATGTATCCGCTCA   4770
GAATCTGCAGTCCACCGTGAAAAGCCCCTTTACACGCGCCTTGGGGATAAACAAATAAAAAGATTTATGTAAGTTTATACATAGGCGAGT
AmpR promoter

EarI
TGAGACAATAACCCTGATAAATGCTTCAATAATATTGAAAAAGGAAGAGTATGAGTATTCAACATTTCCGTGTCGCCCTTATTCCCTTTT   4860
ACTCTGTTATTGGGACTATTTACGAAGTTATTATAACTTTTTCCTTCTCATACTCATAAGTTGTAAAGGCACAGCGGGAATAAGGGAAAA
1 5 10
M S I Q H F R V A L I P F
AmpR promoter
signal sequence
AmpR

TTGCGGCATTTTGCCTTCCTGTTTTTGCTCACCCAGAAACGCTGGTGAAAGTAAAAGATGCTGAAGATCAGTTGGGTGCACGAGTGGGTT   4950
AACGCCGTAAAACGGAAGGACAAAAACGAGTGGGTCTTTGCGACCACTTTCATTTTCTACGACTTCTAGTCAACCCACGTGCTCACCCAA
15 20 25 30 35 40
F A A F C L P V F A H P E T L V K V K D A E D Q L G A R V G
signal sequence
AmpR

XmnI
ACATCGAACTGGATCTCAACAGCGGTAAGATCCTTGAGAGTTTTCGCCCCGAAGAACGTTTTCCAATGATGAGCACTTTTAAAGTTCTGC   5040
TGTAGCTTGACCTAGAGTTGTCGCCATTCTAGGAACTCTCAAAAGCGGGGCTTCTTGCAAAAGGTTACTACTCGTGAAAATTTCAAGACG
45 50 55 60 65 70
Y I E L D L N S G K I L E S F R P E E R F P M M S T F K V L
AmpR

ScaI
TATGTGGCGCGGTATTATCCCGTATTGACGCCGGGCAAGAGCAACTCGGTCGCCGCATACACTATTCTCAGAATGACTTGGTTGAGTACT   5130
ATACACCGCGCCATAATAGGGCATAACTGCGGCCCGTTCTCGTTGAGCCAGCGGCGTATGTGATAAGAGTCTTACTGAACCAACTCATGA
75 80 85 90 95 100
L C G A V L S R I D A G Q E Q L G R R I H Y S Q N D L V E Y
AmpR

CACCAGTCACAGAAAAGCATCTTACGGATGGCATGACAGTAAGAGAATTATGCAGTGCTGCCATAACCATGAGTGATAACACTGCGGCCA   5220
GTGGTCAGTGTCTTTTCGTAGAATGCCTACCGTACTGTCATTCTCTTAATACGTCACGACGGTATTGGTACTCACTATTGTGACGCCGGT
105 110 115 120 125 130
S P V T E K H L T D G M T V R E L C S A A I T M S D N T A A
AmpR
```

PvuI

ACTTACTTCTGACAACGATCGGAGGACCGAAGGAGCTAACCGCTTTTTTGCACAACATGGGGGATCATGTAACTCGCCTTGATCGTTGGG 5310
TGAATGAAGACTGTTGCTAGCCTCCTGGCTTCCTCGATTGGCGAAAAAACGTGTTGTACCCCCTAGTACATTGAGCGGAACTAGCAACCC
135 140 145 150 155 160
N L L L T T I G G P K E L T A F L H N M G D H V T R L D R W
AmpR

AACCGGAGCTGAATGAAGCCATACCAAACGACGAGCGTGACACCACGATGCCTGTAGCAATGGCAACAACGTTGCGCAAACTATTAACTG 5400
TTGGCCTCGACTTACTTCGGTATGGTTTGCTGCTCGCACTGTGGTGCTACGGACATCGTTACCGTTGTTGCAACGCGTTTGATAATTGAC
165 170 175 180 185 190
E P E L N E A I P N D E R D T T M P V A M A T T L R K L L T
AmpR

GCGAACTACTTACTCTAGCTTCCCGGCAACAATTAATAGACTGGATGGAGGCGGATAAAGTTGCAGGACCACTTCTGCGCTCGGCCCTTC 5490
CGCTTGATGAATGAGATCGAAGGGCCGTTGTTAATTATCTGACCTACCTCCGCCTATTTCAACGTCCTGGTGAAGACGCGAGCCGGGAAG
195 200 205 210 215 220
G E L L T L A S R Q Q L I D W M E A D K V A G P L L R S A L
AmpR

BsaI

CGGCTGGCTGGTTTATTGCTGATAAATCTGGAGCCGGTGAGCGTGGGTCTCGCGGTATCATTGCAGCACTGGGGCCAGATGGTAAGCCCT 5580
GCCGACCGACCAAATAACGACTATTTAGACCTCGGCCACTCGCACCCAGAGCGCCATAGTAACGTCGTGACCCCGGTCTACCATTCGGGA
225 230 235 240 245 250
P A G W F I A D K S G A G E R G S R G I I A A L G P D G K P
AmpR

CCCGTATCGTAGTTATCTACACGACGGGGAGTCAGGCAACTATGGATGAACGAAATAGACAGATCGCTGAGATAGGTGCCTCACTGATTA 5670
GGGCATAGCATCAATAGATGTGCTGCCCCTCAGTCCGTTGATACCTACTTGCTTTATCTGTCTAGCGACTCTATCCACGGAGTGACTAAT
255 260 265 270 275 280
S R I V V I Y T T G S Q A T M D E R N R Q I A E I G A S L I
AmpR

AGCATTGGTAACTGTCAGACCAAGTTTACTCATATATACTTTAGATTGATTTAAAACTTCATTTTTAATTTAAAAGGATCTAGGTGAAGA 5760
TCGTAACCATTGACAGTCTGGTTCAAATGAGTATATATGAAATCTAACTAAATTTTGAAGTAAAAATTAAATTTTCCTAGATCCACTTCT
285
K H W *
AmpR

TCCTTTTTGATAATCTCATGACCAAAATCCCTTAACGTGAGTTTTCGTTCCACTGAGCGTCAGACCCCGTAGAAAAGATCAAAGGATCTT 5850
AGGAAAAACTATTAGAGTACTGGTTTTAGGGAATTGCACTCAAAAGCAAGGTGACTCGCAGTCTGGGGCATCTTTTCTAGTTTCCTAGAA

CTTGAGATCCTTTTTTTCTGCGCGTAATCTGCTGCTTGCAAACAAAAAAACCACCGCTACCAGCGGTGGTTTGTTTGCCGGATCAAGAGC 5940
GAACTCTAGGAAAAAAAGACGCGCATTAGACGACGAACGTTTGTTTTTTTGGTGGCGATGGTCGCCACCAAACAAACGGCCTAGTTCTCG
ori

TACCAACTCTTTTTCCGAAGGTAACTGGCTTCAGCAGAGCGCAGATACCAAATACTGTCCTTCTAGTGTAGCCGTAGTTAGGCCACCACT 6030
ATGGTTGAGAAAAAGGCTTCCATTGACCGAAGTCGTCTCGCGTCTATGGTTTATGACAGGAAGATCACATCGGCATCAATCCGGTGGTGA
ori

TCAAGAACTCTGTAGCACCGCCTACATACCTCGCTCTGCTAATCCTGTTACCAGTGGCTGCTGCCAGTGGCGATAAGTCGTGTCTTACCG 6120
AGTTCTTGAGACATCGTGGCGGATGTATGGAGCGAGACGATTAGGACAATGGTCACCGACGACGGTCACCGCTATTCAGCACAGAATGGC
ori

GGTTGGACTCAAGACGATAGTTACCGGATAAGGCGCAGCGGTCGGGCTGAACGGGGGGTTCGTGCACACAGCCCAGCTTGGAGCGAACGA 6210
CCAACCTGAGTTCTGCTATCAATGGCCTATTCCGCGTCGCCAGCCCGACTTGCCCCCCAAGCACGTGTGTCGGGTCGAACCTCGCTTGCT
ori

```
CCTACACCGAACTGAGATACCTACAGCGTGAGCTATGAGAAAGCGCCACGCTTCCCGAAGGGAGAAAGGCGGACAGGTATCCGGTAAGCG    6300
GGATGTGGCTTGACTCTATGGATGTCGCACTCGATACTCTTTCGCGGTGCGAAGGGCTTCCCTCTTTCCGCCTGTCCATAGGCCATTCGC
ori

GCAGGGTCGGAACAGGAGAGCGCACGAGGGAGCTTCCAGGGGGAAACGCCTGGTATCTTTATAGTCCTGTCGGGTTTCGCCACCTCTGAC    6390
CGTCCCAGCCTTGTCCTCTCGCGTGCTCCCTCGAAGGTCCCCCTTTGCGGACCATAGAAATATCAGGACAGCCCAAAGCGGTGGAGACTG
ori

TTGAGCGTCGATTTTTGTGATGCTCGTCAGGGGGGCGGAGCCTATGGAAAAACGCCAGCAACGCGGCCTTTTTACGGTTCCTGGCCTTTT    6480
AACTCGCAGCTAAAAACACTACGAGCAGTCCCCCCGCCTCGGATACCTTTTTGCGGTCGTTGCGCCGGAAAAATGCCAAGGACCGGAAAA
ori

GCTGGCCTTTTGCTCACATGT    3'
                     ...    6501
CGACCGGAAAACGAGTGTACA    5'
```
