# Supplementary material for: Truncated mini LRP1 transports cargo from luminal to basolateral side across the blood brain barrier
Source: Fluids Barriers CNS. 2024 Sep 17;21:74. doi: 10.1186/s12987-024-00573-1 (PMC11409491; doi:10.1186/s12987-024-00573-1)
Supplement: Supplementary file 9 — Supplementary Material 9 [file 12987_2024_573_MOESM9_ESM.pdf]

```
5' TTTGAAAGACCCCACCCGTAGGTGGCAAGCTAGCTTAAGTAACGCCACTTTGCAAGGCATGGAAAAATACATAACTGAGAATAGAAAAGT 90
3' AAACTTTCTGGGGTGGGCATCCACCGTTCGATCGAATTCATTGCGGTGAAACGTTCCGTACCTTTTTATGTATTGACTCTTATCTTTTCA

TCAGATCAAGGTCAGGAACAAAGAAACAGCTGAATACCAAACAGGATATCTGTGGTAAGCGGTTCCTGCCCCGGCTCAGGGCCAAGAACA 180
AGTCTAGTTCCAGTCCTTGTTTCTTTGTCGACTTATGGTTTGTCCTATAGACACCATTCGCCAAGGACGGGGCCGAGTCCCGGTTCTTGT

GATGAGACAGCTGAGTGATGGGCCAAACAGGATATCTGTGGTAAGCAGTTCCTGCCCCGGCTCGGGGCCAAGAACAGATGGTCCCCAGAT 270
CTACTCTGTCGACTCACTACCCGGTTTGTCCTATAGACACCATTCGTCAAGGACGGGGCCGAGCCCCGGTTCTTGTCTACCAGGGGTCTA

BbvCI
GCGGTCCAGCCCTCAGCAGTTTCTAGTGAATCATCAGATGTTTCCAGGGTGCCCCAAGGACCTGAAAATGACCCTGTACCTTATTTGAAC 360
CGCCAGGTCGGGAGTCGTCAAAGATCACTTAGTAGTCTACAAAGGTCCCACGGGGTTCCTGGACTTTTACTGGGACATGGAATAAACTTG

AscI
TAACCAATCAGTTCGCTTCTCGCTTCTGTTCGCGCGCTTCCGCTCTCCGAGCTCAATAAAAGAGCCCACAACCCCTCACTCGGCGCGCCA 450
ATTGGTTAGTCAAGCGAAGAGCGAAGACAAGCGCGCGAAGGCGAGAGGCTCGAGTTATTTTCTCGGGTGTTGGGGAGTGAGCCGCGCGGT
5' LTR (truncated)

GTCTTCCGATAGACTGCGTCGCCCGGGTACCCGTATTCCCAATAAAGCCTCTTGCTGTTTGCATCCGAATCGTGGTCTCGCTGTTCCTTG 540
CAGAAGGCTATCTGACGCAGCGGGCCCATGGGCATAAGGGTTATTTCGGAGAACGACAAACGTAGGCTTAGCACCAGAGCGACAAGGAAC
5' LTR (truncated)

GGAGGGTCTCCTCTGAGTGATTGACTACCCACGACGGGGGTCTTTCATTTGGGGGCTCGTCCGGGATTTGGAGACCCCTGCCCAGGGACC 630
CCTCCCAGAGGAGACTCACTAACTGATGGGTGCTGCCCCCAGAAAGTAAACCCCCGAGCAGGCCCTAAACCTCTGGGGACGGGTCCCTGG
5' LTR (truncated)

ACCGACCCACCACCGGGAGGTAAGCTGGCCAGCAACTTATCTGTGTCTGTCCGATTGTCTAGTGTCTATGTTTGATGTTATGCGCCTGCG 720
TGGCTGGGTGGTGGCCCTCCATTCGACCGGTCGTTGAATAGACACAGACAGGCTAACAGATCACAGATACAAACTACAATACGCGGACGC
MMLV Ψ

SpeI
TCTGTACTAGTTAGCTAACTAGCTCTGTATCTGGCGGACCCGTGGTGGAACTGACGAGTTCTGAACACCCGGCCGCAACCCTGGGAGACG 810
AGACATGATCAATCGATTGATCGAGACATAGACCGCCTGGGCACCACCTTGACTGCTCAAGACTTGTGGGCCGGCGTTGGGACCCTCTGC
MMLV Ψ

TCCCAGGGACTTTGGGGGCCGTTTTTGTGGCCCGACCTGAGGAAGGGAGTCGATGTGGAATCCGACCCCGTCAGGATATGTGGTTCTGGT 900
AGGGTCCCTGAAACCCCCGGCAAAAACACCGGGCTGGACTCCTTCCCTCAGCTACACCTTAGGCTGGGGCAGTCCTATACACCAAGACCA
MMLV Ψ

AGGAGACGAGAACCTAAAACAGTTCCCGCCTCCGTCTGAATTTTTGCTTTCGGTTTGGAACCGAAGCCGCGCGTCTTGTCTGCTGCAGCG 990
TCCTCTGCTCTTGGATTTTGTCAAGGGCGGAGGCAGACTTAAAAACGAAAGCCAAACCTTGGCTTCGGCGCGCAGAACAGACGACGTCGC

CTGCAGCATCGTTCTGTGTTGTCTCTGTCTGACTGTGTTTCTGTATTTGTCTGAAAATTAGGGCCAGACTGTTACCACTCCCTTAAGTTT 1080
GACGTCGTAGCAAGACACAACAGAGACAGACTGACACAAAGACATAAACAGACTTTTAATCCCGGTCTGACAATGGTGAGGGAATTCAAA
gag (truncated) >

BstEII
GACCTTAGGTCACTGGAAAGATGTCGAGCGGATCGCTCACAACCAGTCGGTAGATGTCAAGAAGAGACGTTGGGTTACCTTCTGCTCTGC 1170
CTGGAATCCAGTGACCTTTCTACAGCTCGCCTAGCGAGTGTTGGTCAGCCATCTACAGTTCTTCTCTGCAACCCAATGGAAGACGAGACG
gag (truncated) >
```

```
AGAATGGCCAACCTTTAACGTCGGATGGCCGCGAGACGGCACCTTTAACCGAGACCTCATCACCCAGGTTAAGATCAAGGTCTTTTCACC  1260
TCTTACCGGTTGGAAATTGCAGCCTACCGGCGCTCTGCCGTGGAAATTGGCTCTGGAGTAGTGGGTCCAATTCTAGTTCCAGAAAAGTGG
gag (truncated)

TGGCCCGCATGGACACCCAGACCAGGTCCCCTACATCGTGACCTGGGAAGCCTTGGCTTTTGACCCCCCTCCCTGGGTCAAGCCCTTTGT  1350
ACCGGGCGTACCTGTGGGTCTGGTCCAGGGGATGTAGCACTGGACCCTTCGGAACCGAAAACTGGGGGGAGGGACCCAGTTCGGGAAACA
gag (truncated)

ACACCCTAAGCCTCCGCCTCCTCTTCCTCCATCCGCCCCGTCTCTCCCCCTTGAACCTCCTCGTTCGACCCCGCCTCGATCCTCCCTTTA  1440
TGTGGGATTCGGAGGCGGAGGAGAAGGAGGTAGGCGGGGCAGAGAGGGGGAACTTGGAGGAGCAAGCTGGGGCGGAGCTAGGAGGGAAAT
gag (truncated)

TCCAGCCCTCACTCCTTCTCTAGGCGCCGGAATTCCGATCTGATCCATGGCCAAGCCTTTGTCTCAAGAAGAATCCACCCTCATTGAAAG  1530
AGGTCGGGAGTGAGGAAGAGATCCGCGGCCTTAAGGCTAGACTAGGTACCGGTTCGGAAACAGAGTTCTTCTTAGGTGGGAGTAACTTTC
                                                                1   5   10   15
                                                                Met Ala Lys Pro Leu Ser Gln Glu Glu Ser Thr Leu Ile Glu Arg
gag (truncated)                                                 BSD

AGCAACGGCTACAATCAACAGCATCCCCATCTCTGAAGACTACAGCGTCGCCAGCGCAGCTCTCTCTAGCGACGGCCGCATCTTCACTGG  1620
TCGTTGCCGATGTTAGTTGTCGTAGGGGTAGAGACTTCTGATGTCGCAGCGGTCGCGTCGAGAGAGATCGCTGCCGGCGTAGAAGTGACC
20   25   30   35   40   45
Ala Thr Ala Thr Ile Asn Ser Ile Pro Ile Ser Glu Asp Tyr Ser Val Ala Ser Ala Ala Leu Ser Ser Asp Gly Arg Ile Phe Thr Gly
BSD

TGTCAATGTATATCATTTTACTGGGGGACCTTGTGCAGAACTCGTGGTGCTGGGCACTGCTGCTGCTGCGGCAGCTGGCAACCTGACTTG  1710
ACAGTTACATATAGTAAAATGACCCCCTGGAACACGTCTTGAGCACCACGACCCGTGACGACGACGACGCCGTCGACCGTTGGACTGAAC
50   55   60   65   70   75
Val Asn Val Tyr His Phe Thr Gly Gly Pro Cys Ala Glu Leu Val Val Leu Gly Thr Ala Ala Ala Ala Ala Ala Gly Asn Leu Thr Cys
BSD

NruI*   BsaBI*
TATCGTCGCGATCGGAAATGAGAACAGGGGCATCTTGAGCCCCTGCGGACGGTGCCGACAGGTGCTTCTCGATCTGCATCCTGGGATCAA  1800
ATAGCAGCGCTAGCCTTTACTCTTGTCCCCGTAGAACTCGGGGACGCCTGCCACGGCTGTCCACGAAGAGCTAGACGTAGGACCCTAGTT
80   85   90   95   100   105
Ile Val Ala Ile Gly Asn Glu Asn Arg Gly Ile Leu Ser Pro Cys Gly Arg Cys Arg Gln Val Leu Leu Asp Leu His Pro Gly Ile Lys
BSD

AGCCATAGTGAAGGACAGTGATGGACAGCCGACGGCAGTTGGGATTCGTGAATTGCTGCCCTCTGGTTATGTGTGGGAGGGCTAAGCATG  1890
TCGGTATCACTTCCTGTCACTACCTGTCGGCTGCCGTCAACCCTAAGCACTTAACGACGGGAGACCAATACACACCCTCCCGATTCGTAC
110   115   120   125   130
Ala Ile Val Lys Asp Ser Asp Gly Gln Pro Thr Ala Val Gly Ile Arg Glu Leu Leu Pro Ser Gly Tyr Val Trp Glu Gly *
BSD

CCCGACGGCGAGGATCTCGTCGTGACCCATGGCGATGCCTGCTTGCCGAATATCATGGTGGAAAATGGCCGCTTTTCTGGATTCATCGAC  1980
GGGCTGCCGCTCCTAGAGCAGCACTGGGTACCGCTACGGACGAACGGCTTATAGTACCACCTTTTACCGGCGAAAAGACCTAAGTAGCTG

NgoMIV  NaeI   RsrII
TGTGGCCGGCTGGGTGTGGCGGACCGCTATCAGGACATAGCGTTGGCTACCCGTGATATTGCTGAAGAGCTTGGCGGCGAATGGGCTGAC  2070
ACACCGGCCGACCCACACCGCCTGGCGATAGTCCTGTATCGCAACCGATGGGCACTATAACGACTTCTCGAACCGCCGCTTACCCGACTG

CGCTTCCTCGTGCTTTACGGTATCGCCGCTCCCGATTCGCAGCGCATCGCCTTCTATCGCCTTCTTGACGAGTTCTTCTGAGCGGGACTC  2160
GCGAAGGAGCACGAAATGCCATAGCGGCGAGGGCTAAGCGTCGCGTAGCGGAAGATAGCGGAAGAACTGCTCAAGAAGACTCGCCCTGAG

TGGGGTTCGTAATAGTAATCAATTACGGGGTCATTAGTTCATAGCCCATATATGGAGTTCCGCGTTACATAACTTACGGTAAATGGCCCG  2250
ACCCCAAGCATTATCATTAGTTAATGCCCCAGTAATCAAGTATCGGGTATATACCTCAAGGCGCAATGTATTGAATGCCATTTACCGGGC
                                                                CMV enhancer
```

```
CCTGGCTGACCGCCCAACGACCCCCGCCCATTGACGTCAATAATGACGTATGTTCCCATAGTAACGCCAATAGGGACTTTCCATTGACGT   2340
GGACCGACTGGCGGGTTGCTGGGGGCGGGTAACTGCAGTTATTACTGCATACAAGGGTATCATTGCGGTTATCCCTGAAAGGTAACTGCA
CMV enhancer
```

```
CAATGGGTGGAGTATTTACGGTAAACTGCCCACTTGGCAGTACATCAAGTGTATCATATGCCAAGTACGCCCCCTATTGACGTCAATGAC   2430
GTTACCCACCTCATAAATGCCATTTGACGGGTGAACCGTCATGTAGTTCACATAGTATACGGTTCATGCGGGGGATAACTGCAGTTACTG
CMV enhancer
```

```
SnaBI
GGTAAATGGCCCGCCTGGCATTATGCCCAGTACATGACCTTATGGGACTTTCCTACTTGGCAGTACATCTACGTATTAGTCATCGCTATT   2520
CCATTTACCGGGCGGACCGTAATACGGGTCATGTACTGGAATACCCTGAAAGGATGAACCGTCATGTAGATGCATAATCAGTAGCGATAA
CMV enhancer
```

```
ACCATGGTGATGCGGTTTTGGCAGTACATCAATGGGCGTGGATAGCGGTTTGACTCACGGGGATTTCCAAGTCTCCACCCCATTGACGTC   2610
TGGTACCACTACGCCAAAACCGTCATGTAGTTACCCGCACCTATCGCCAAACTGAGTGCCCCTAAAGGTTCAGAGGTGGGGTAACTGCAG
1 Val Met Arg Phe 5 Trp Gln Tyr Ile Asn 10 Gly Arg Gly * Arg 15 Phe Asp Ser Arg Gly 20 Phe Pro Ser Leu His 25 Pro Ile Asp Val
CMV promoter
CMV enhancer
```

```
pLBCX CMV prom fwd
cggtaggcgt
AATGGGAGTTTGTTTTGGCACCAAAATCAACGGGACTTTCCAAAATGTCGTAACAACTCCGCCCCATTGACGCAAATGGGCGGTAGGCGT   2700
TTACCCTCAAACAAAACCGTGGTTTTAGTTGCCCTGAAAGGTTTTACAGCATTGTTGAGGCGGGGTAACTGCGTTTACCCGCCATCCGCA
Asn 30 Gly Ser Leu Phe Trp 35 His Gln Asn Gln Arg 40 Asp Phe Pro Lys Cys 45 Arg Asn Asn Ser Ala 50 Pro Leu Thr Gln Met 55 Gly Gly Arg Arg
CMV promoter
```

```
pLBCX CMV prom fwd
gtacggtgggaggtctat
XhoI
PaeR7I
GTACGGTGGGAGGTCTATATAAGCAGAGCTGGTTTAGTGAACCGTCAGATCCGCTAGCGCTACCGGACTCAGATCTCGAGCTCAAGCTTG   2790
CATGCCACCCTCCAGATATATTCGTCTCGACCAAATCACTTGGCAGTCTAGGCGATCGCGATGGCCTGAGTCTAGAGCTCGAGTTCGAAC
Val 60 Arg Trp Glu Val Tyr 65 Ile Ser Arg Ala Gly Leu Val Asn Arg Gln Ile Arg *
CMV promoter (in frame with CMV promoter)
```

```
SfiI NotI
NotI fwd
TTTT GCGGCCGCGCCACCATGCTGACCCCGCCGTT
ss MYC oligo
TCAGCTCTGGTCGCGGCGGCTATCGAC
TTTGGCCGAGGCGGCCGCGCCACCAtgctgaccccgccgttgctcctgctgctgcccctgctctcagctctggtcgcggcggctatcgac   2880
AAACCGGCTCCGCCGGCGCGGTGGTacgactggggcggcaacgaggacgacgacggggacgagagtcgagaccagcgccgccgatagctg
1 Met Leu Thr Pro 5 Pro Leu Leu Leu Leu 10 Leu Pro Leu Leu Ser 15 Ala Leu Val Ala Ala 20 Ala Ile Asp
LRP1mini
Leader sequence
```

```
BamHI
ss MYC oligo
GCCCCTGGAGAACAAAAACTTATTTCTGAAGAAGATCTGGGATCCACCCACTGCAAAGACAAGAAGGAGTTT
gccccTGGAGAACAAAAACTTATTTCTGAAGAAGATCTGGgatccacccactgcaaagacaagaaggagtttctgtgccggaaccagcgc   2970
cggggaCCTCTTGTTTTTGAATAAAGACTTCTTCTAGACCctaggtgggtgacgtttctgttcttcctcaaagacacggccttggtcgcg
Ala Pro 25 Gly Glu Gln Lys Leu 30 Ile Ser Glu Glu Asp 35 Leu Gly Ser Thr His 40 Cys Lys Asp Lys Lys 45 Glu Phe Leu Cys Arg 50 Asn Gln Arg
LRP1mini
1 Glu Gln Lys Leu 5 Ile Ser Glu Glu Asp 10 Leu Gly Ser Thr His Cys Lys Asp Lys Lys Glu Phe Leu Cys Arg Asn Gln Arg
Myc (in frame with Myc)
Leader sequence
```

tgcctctcctcctccctgcgctgcaacatgttcgatgactgcggggacggctctgacgaggaggactgcagcatcgaccccaagctgacc 3060
acggagaggaggagggacgcgacgttgtacaagctactgacgcccctgccgagactgctcctcctgacgtcgtagctggggttcgactgg

55 60 65 70 75 80
Cys Leu Ser Ser Ser Leu Arg Cys Asn Met Phe Asp Asp Cys Gly Asp Gly Ser Asp Glu Glu Asp Cys Ser Ile Asp Pro Lys Leu Thr
LRP1mini
Cys Leu Ser Ser Ser Leu Arg Cys Asn Met Phe Asp Asp Cys Gly Asp Gly Ser Asp Glu Glu Asp Cys Ser Ile Asp Pro Lys Leu Thr
(in frame with Myc)

FspAI

agctgcgccaccaatgccagcatctgtggggacgaggcacgctgcgtgcgcaccgagaaagcggcctactgtgcctgccgctcgggcttc 3150
tcgacgcggtggttacggtcgtagacacccctgctccgtgcgacgcacgcgtggctctttcgccggatgacacggacggcgagcccgaag

85 90 95 100 105 110
Ser Cys Ala Thr Asn Ala Ser Ile Cys Gly Asp Glu Ala Arg Cys Val Arg Thr Glu Lys Ala Ala Tyr Cys Ala Cys Arg Ser Gly Phe
LRP1mini
Ser Cys Ala Thr Asn Ala Ser Ile Cys Gly Asp Glu Ala Arg Cys Val Arg Thr Glu Lys Ala Ala Tyr Cys Ala Cys Arg Ser Gly Phe
(in frame with Myc)

DraIII

cacaccgtgcccggccagcccggatgccaagacatcaacgagtgcctgcgcttcggcacctgctcccagctctgcaacaacaccaagggc 3240
gtgtggcacgggccggtcgggcctacggttctgtagttgctcacggacgcgaagccgtggacgagggtcgagacgttgttgtggttcccg

115 120 125 130 135 140
His Thr Val Pro Gly Gln Pro Gly Cys Gln Asp Ile Asn Glu Cys Leu Arg Phe Gly Thr Cys Ser Gln Leu Cys Asn Asn Thr Lys Gly
LRP1mini
His Thr Val Pro Gly Gln Pro Gly Cys Gln Asp Ile Asn Glu Cys Leu Arg Phe Gly Thr Cys Ser Gln Leu Cys Asn Asn Thr Lys Gly
(in frame with Myc)

ggccacctctgcagctgcgctcggaacttcatgaagacgcacaacacctgcaaggccgaaggctctgagtaccaggtcctgtacatcgct 3330
ccggtggagacgtcgacgcgagccttgaagtacttctgcgtgttgtggacgttccggcttccgagactcatggtccaggacatgtagcga

145 150 155 160 165 170
Gly His Leu Cys Ser Cys Ala Arg Asn Phe Met Lys Thr His Asn Thr Cys Lys Ala Glu Gly Ser Glu Tyr Gln Val Leu Tyr Ile Ala
LRP1mini
Gly His Leu Cys Ser Cys Ala Arg Asn Phe Met Lys Thr His Asn Thr Cys Lys Ala Glu Gly Ser Glu Tyr Gln Val Leu Tyr Ile Ala
(in frame with Myc)

BsmI

gatgacaatgagatccgcagcctgttccccggccaccccattcggcttacgagcaggcattccagggtgacgagagtgtccgcattgat 3420
ctactgttactctaggcgtcggacaaggggccggtggggtaagccgaatgctcgtccgtaaggtcccactgctctcacaggcgtaacta

175 180 185 190 195 200
Asp Asp Asn Glu Ile Arg Ser Leu Phe Pro Gly His Pro His Ser Ala Tyr Glu Gln Ala Phe Gln Gly Asp Glu Ser Val Arg Ile Asp
LRP1mini
Asp Asp Asn Glu Ile Arg Ser Leu Phe Pro Gly His Pro His Ser Ala Tyr Glu Gln Ala Phe Gln Gly Asp Glu Ser Val Arg Ile Asp
(in frame with Myc)

gctatggatgtccatgtcaaggctggccgtgtctattggaccaactggcacacgggcaccatctcctaccgcagcctgccacctgctgcg 3510
cgatacctacaggtacagttccgaccggcacagataacctggttgaccgtgtgcccgtggtagaggatggcgtcggacggtggacgacgc

205 210 215 220 225 230
Ala Met Asp Val His Val Lys Ala Gly Arg Val Tyr Trp Thr Asn Trp His Thr Gly Thr Ile Ser Tyr Arg Ser Leu Pro Pro Ala Ala
LRP1mini
Ala Met Asp Val His Val Lys Ala Gly Arg Val Tyr Trp Thr Asn Trp His Thr Gly Thr Ile Ser Tyr Arg Ser Leu Pro Pro Ala Ala
(in frame with Myc)

SgrAI

cctcctaccacttccaaccgccaccggcgacagattgaccggggtgtcacccacctcaacatttcagggctgaagatgcccagaggcatc 3600
ggaggatggtgaaggttggcggtggccgctgtctaactggccccacagtgggtggagttgtaaagtcccgacttctacgggtctccgtag

235 240 245 250 255 260
Pro Pro Thr Thr Ser Asn Arg His Arg Arg Gln Ile Asp Arg Gly Val Thr His Leu Asn Ile Ser Gly Leu Lys Met Pro Arg Gly Ile
LRP1mini
Pro Pro Thr Thr Ser Asn Arg His Arg Arg Gln Ile Asp Arg Gly Val Thr His Leu Asn Ile Ser Gly Leu Lys Met Pro Arg Gly Ile
(in frame with Myc)

gccatcgactgggtggccggaaacgtgtactggaccgactcgggccgagatgtgattgaggtggcgcagatgaagggcgagaaccgcaag 3690
cggtagctgacccaccggcctttgcacatgacctggctgagcccggctctacactaactccaccgcgtctacttcccgctcttggcgttc

265 270 275 280 285 290
Ala Ile Asp Trp Val Ala Gly Asn Val Tyr Trp Thr Asp Ser Gly Arg Asp Val Ile Glu Val Ala Gln Met Lys Gly Glu Asn Arg Lys
LRP1mini
Ala Ile Asp Trp Val Ala Gly Asn Val Tyr Trp Thr Asp Ser Gly Arg Asp Val Ile Glu Val Ala Gln Met Lys Gly Glu Asn Arg Lys
(in frame with Myc)

acgctcatctcgggcatgattgacgagccccacgccattgtggtggacccactgagggggaccatgtactggtcagactggggcaaccac 3780
tgcgagtagagcccgtactaactgctcggggtgcggtaacaccacctgggtgactccccctggtacatgaccagtctgaccccgttggtg

295 300 305 310 315 320
Thr Leu Ile Ser Gly Met Ile Asp Glu Pro His Ala Ile Val Val Asp Pro Leu Arg Gly Thr Met Tyr Trp Ser Asp Trp Gly Asn His
LRP1mini
Thr Leu Ile Ser Gly Met Ile Asp Glu Pro His Ala Ile Val Val Asp Pro Leu Arg Gly Thr Met Tyr Trp Ser Asp Trp Gly Asn His
(in frame with Myc)

cccaagattgagacggcagcgatggatgggacgcttcgggagacactggtgcaggacaacattcagtggcccacaggcctggccgtggat 3870
gggttctaactctgccgtcgctacctaccctgcgaagccctctgtgaccacgtcctgttgtaagtcaccgggtgtccggaccggcaccta

325 330 335 340 345 350
Pro Lys Ile Glu Thr Ala Ala Met Asp Gly Thr Leu Arg Glu Thr Leu Val Gln Asp Asn Ile Gln Trp Pro Thr Gly Leu Ala Val Asp
LRP1mini
Pro Lys Ile Glu Thr Ala Ala Met Asp Gly Thr Leu Arg Glu Thr Leu Val Gln Asp Asn Ile Gln Trp Pro Thr Gly Leu Ala Val Asp
(in frame with Myc)

tatcacaatgagcggctgtactgggcagacgccaagctttcagtcatcggcagcatccggctcaatggcacggaccccattgtggctgct 3960
atagtgttactcgccgacatgacccgtctgcggttcgaaagtcagtagccgtcgtaggccgagttaccgtgcctggggtaacaccgacga

355 360 365 370 375 380
Tyr His Asn Glu Arg Leu Tyr Trp Ala Asp Ala Lys Leu Ser Val Ile Gly Ser Ile Arg Leu Asn Gly Thr Asp Pro Ile Val Ala Ala
LRP1mini
Tyr His Asn Glu Arg Leu Tyr Trp Ala Asp Ala Lys Leu Ser Val Ile Gly Ser Ile Arg Leu Asn Gly Thr Asp Pro Ile Val Ala Ala
(in frame with Myc)

gacagcaaacgaggcctaagtcaccccttcagcatcgacgtctttgaggattacatctatggtgtcacctacatcaataatcgtgtcttc 4050
ctgtcgtttgctccggattcagtggggaagtcgtagctgcagaaactcctaatgtagataccacagtggatgtagttattagcacagaag

385 390 395 400 405 410
Asp Ser Lys Arg Gly Leu Ser His Pro Phe Ser Ile Asp Val Phe Glu Asp Tyr Ile Tyr Gly Val Thr Tyr Ile Asn Asn Arg Val Phe
LRP1mini
Asp Ser Lys Arg Gly Leu Ser His Pro Phe Ser Ile Asp Val Phe Glu Asp Tyr Ile Tyr Gly Val Thr Tyr Ile Asn Asn Arg Val Phe
(in frame with Myc)

aagatccataagtttggccacagccccttggtcaacctgacagggggcctgagccacgcctctgacgtggtcctttaccatcagcacaag 4140
ttctaggtattcaaaccggtgtcggggaaccagttggactgtcccccggactcggtgcggagactgcaccaggaaatggtagtcgtgttc

415 420 425 430 435 440
Lys Ile His Lys Phe Gly His Ser Pro Leu Val Asn Leu Thr Gly Gly Leu Ser His Ala Ser Asp Val Val Leu Tyr His Gln His Lys
LRP1mini
Lys Ile His Lys Phe Gly His Ser Pro Leu Val Asn Leu Thr Gly Gly Leu Ser His Ala Ser Asp Val Val Leu Tyr His Gln His Lys
(in frame with Myc)

ggtattcaaaccggtgtcg
MYC reverse

cagcccgaagtgaccaacccatgtgaccgcaagaaatgcgagtggctctgcctgctgagccccagtgggcctgtctgcacctgtcccaat 4230
gtcgggcttcactggttgggtacactggcgttctttacgctcaccgagacggacgactcggggtcacccggacagacgtggacagggtta

445 450 455 460 465 470
Gln Pro Glu Val Thr Asn Pro Cys Asp Arg Lys Lys Cys Glu Trp Leu Cys Leu Leu Ser Pro Ser Gly Pro Val Cys Thr Cys Pro Asn
LRP1mini
Gln Pro Glu Val Thr Asn Pro Cys Asp Arg Lys Lys Cys Glu Trp Leu Cys Leu Leu Ser Pro Ser Gly Pro Val Cys Thr Cys Pro Asn
(in frame with Myc)

AleI

gggaagcggctggacaacggcacatgcgtgcctgtgccctctccaacgcccccccccagatgctccccggcctggaacctgtaacctgcag 4320
cccttcgccgacctgttgccgtgtacgcacggacacgggagaggttgcgggggggggtctacgaggggccggaccttggacattggacgtc

475 480 485 490 495 500
Gly Lys Arg Leu Asp Asn Gly Thr Cys Val Pro Val Pro Ser Pro Thr Pro Pro Pro Asp Ala Pro Arg Pro Gly Thr Cys Asn Leu Gln
LRP1mini
Gly Lys Arg Leu Asp Asn Gly Thr Cys Val Pro Val Pro Ser Pro Thr Pro Pro Pro Asp Ala Pro Arg Pro Gly Thr Cys Asn Leu Gln
(in frame with Myc)

```
tgcttcaacggtggcagctgtttcctcaatgcacggaggcagcccaagtgccgctgccaaccccgctacacgggtgacaagtgtgaactg  4410
acgaagttgccaccgtcgacaaaggagttacgtgcctccgtcgggttcacggcgacggttggggcgatgtgcccactgttcacacttgac
        505                 510                 515                 520                 525                 530
Cys Phe Asn Gly Gly Ser Cys Phe Leu Asn Ala Arg Arg Gln Pro Lys Cys Arg Cys Gln Pro Arg Tyr Thr Gly Asp Lys Cys Glu Leu
LRP1mini
Cys Phe Asn Gly Gly Ser Cys Phe Leu Asn Ala Arg Arg Gln Pro Lys Cys Arg Cys Gln Pro Arg Tyr Thr Gly Asp Lys Cys Glu Leu
(in frame with Myc)
```

```
                                                                PmlI
gaccagtgctgggagcactgtcgcaatggggcacctgtgctgcctcccctctggcatgcccacgtgccggtgccccacgggcttcacg  4500
ctggtcacgaccctcgtgacagcgttaccccgtggacacgacggaggggagaccgtacgggtgcacggccacggggtgcccgaagtgc
        535                 540                 545                 550                 555                 560
Asp Gln Cys Trp Glu His Cys Arg Asn Gly Gly Thr Cys Ala Ala Ser Pro Ser Gly Met Pro Thr Cys Arg Cys Pro Thr Gly Phe Thr
LRP1mini
Asp Gln Cys Trp Glu His Cys Arg Asn Gly Gly Thr Cys Ala Ala Ser Pro Ser Gly Met Pro Thr Cys Arg Cys Pro Thr Gly Phe Thr
(in frame with Myc)
```

```
ggccccaaatgcacccagcaggtgtgtgcgggctactgtgccaacaacagcacctgcactgtcaaccagggcaaccagccccagtgccga  4590
ccggggtttacgtgggtcgtccacacacgcccgatgacacggttgttgtcgtggacgtgacagttggtcccgttggtcggggtcacggct
        565                 570                 575                 580                 585                 590
Gly Pro Lys Cys Thr Gln Gln Val Cys Ala Gly Tyr Cys Ala Asn Asn Ser Thr Cys Thr Val Asn Gln Gly Asn Gln Pro Gln Cys Arg
LRP1mini
Gly Pro Lys Cys Thr Gln Gln Val Cys Ala Gly Tyr Cys Ala Asn Asn Ser Thr Cys Thr Val Asn Gln Gly Asn Gln Pro Gln Cys Arg
(in frame with Myc)
```

```
tgcctacccggcttcctgggcgaccgctgccagtaccggcagtgctctggctactgtgagaactttggcacatgccagatggctgctgat  4680
acggatgggccgaaggacccgctggcgacggtcatggccgtcacgagaccgatgacactcttgaaaccgtgtacggtctaccgacgacta
        595                 600                 605                 610                 615                 620
Cys Leu Pro Gly Phe Leu Gly Asp Arg Cys Gln Tyr Arg Gln Cys Ser Gly Tyr Cys Glu Asn Phe Gly Thr Cys Gln Met Ala Ala Asp
LRP1mini
Cys Leu Pro Gly Phe Leu Gly Asp Arg Cys Gln Tyr Arg Gln Cys Ser Gly Tyr Cys Glu Asn Phe Gly Thr Cys Gln Met Ala Ala Asp
(in frame with Myc)
```

```
ggctcccgacaatgccgctgcactgcctactttgagggatcgaggtgtgaggtgaacaagtgcagccgctgtctcgaagggggcctgtgtg  4770
ccgagggctgttacggcgacgtgacggatgaaactccctagctccacactccacttgttcacgtcggcgacagagcttccccggacacac
        625                 630                 635                 640                 645                 650
Gly Ser Arg Gln Cys Arg Cys Thr Ala Tyr Phe Glu Gly Ser Arg Cys Glu Val Asn Lys Cys Ser Arg Cys Leu Glu Gly Ala Cys Val
LRP1mini
Gly Ser Arg Gln Cys Arg Cys Thr Ala Tyr Phe Glu Gly Ser Arg Cys Glu Val Asn Lys Cys Ser Arg Cys Leu Glu Gly Ala Cys Val
(in frame with Myc)
```

```
gtcaacaagcagagtggggatgtcacctgcaactgcacggatggccgggtggccccagctgtctgacctgcgtcggccactgcagcaat  4860
cagttgttcgtctcacccctacagtggacgttgacgtgcctaccggcccaccggggtcgacagactggacgcagccggtgacgtcgtta
        655                 660                 665                 670                 675                 680
Val Asn Lys Gln Ser Gly Asp Val Thr Cys Asn Cys Thr Asp Gly Arg Val Ala Pro Ser Cys Leu Thr Cys Val Gly His Cys Ser Asn
LRP1mini
Val Asn Lys Gln Ser Gly Asp Val Thr Cys Asn Cys Thr Asp Gly Arg Val Ala Pro Ser Cys Leu Thr Cys Val Gly His Cys Ser Asn
(in frame with Myc)
```

```
ggcggctcctgtaccatgaacagcaaaatgatgcctgagtgccagtgcccaccccacatgacagggccccggtgtgaggagcacgtcttc  4950
ccgccgaggacatggtacttgtcgttttactacggactcacggtcacgggtggggtgtactgtcccggggccacactcctcgtgcagaag
        685                 690                 695                 700                 705                 710
Gly Gly Ser Cys Thr Met Asn Ser Lys Met Met Pro Glu Cys Gln Cys Pro Pro His Met Thr Gly Pro Arg Cys Glu Glu His Val Phe
LRP1mini
Gly Gly Ser Cys Thr Met Asn Ser Lys Met Met Pro Glu Cys Gln Cys Pro Pro His Met Thr Gly Pro Arg Cys Glu Glu His Val Phe
(in frame with Myc)
```

```
                                                                                   LRP1 CT fwd (Seq)
                                                                                                CTGG
agccagcagcagccaggacatatagcctccatcctaatccctctgctgttgctgctgctgctggttctggtggccggagtggtattctgg  5040
tcggtcgtcgtcggtcctgtatatcggaggtaggattagggagacgacaacgacgacgacgaccaagaccaccggcctcaccataagacc
        715                 720                 725                 730                 735                 740
Ser Gln Gln Gln Pro Gly His Ile Ala Ser Ile Leu Ile Pro Leu Leu Leu Leu Leu Leu Leu Val Leu Val Ala Gly Val Val Phe Trp
LRP1mini
Ser Gln Gln Gln Pro Gly His Ile Ala Ser Ile Leu Ile Pro Leu Leu Leu Leu Leu Leu Leu Val Leu Val Ala Gly Val Val Phe Trp
(in frame with Myc)
```

LRP1 CT fwd (Seq)
TATAAGCGGCGAGTC

tataagcggcgagtccaaggggctaagggcttccagcaccaacggatgaccaacggggccatgaacgtggagattggaaaccccacctac 5130
atattcgccgctcaggttccccgattcccgaaggtcgtggttgcctactggttgccccggtacttgcacctctaacctttggggtggatg

745 750 755 760 765 770
Tyr Lys Arg Arg Val Gln Gly Ala Lys Gly Phe Gln His Gln Arg Met Thr Asn Gly Ala Met Asn Val Glu Ile Gly Asn Pro Thr Tyr
LRP1mini

Tyr Lys Arg Arg Val Gln Gly Ala Lys Gly Phe Gln His Gln Arg Met Thr Asn Gly Ala Met Asn Val Glu Ile Gly Asn Pro Thr Tyr
(in frame with Myc)

aagatgtacgaaggcggagagcctgatgatgtgggaggcctactggacgctgactttgccctggaccctgacaagcccaccaacttcacc 5220
ttctacatgcttccgcctctcggactactacaccctccggatgacctgcgactgaaacgggacctgggactgttcgggtggttgaagtgg

775 780 785 790 795 800
Lys Met Tyr Glu Gly Gly Glu Pro Asp Asp Val Gly Gly Leu Leu Asp Ala Asp Phe Ala Leu Asp Pro Asp Lys Pro Thr Asn Phe Thr
LRP1mini

Lys Met Tyr Glu Gly Gly Glu Pro Asp Asp Val Gly Gly Leu Leu Asp Ala Asp Phe Ala Leu Asp Pro Asp Lys Pro Thr Asn Phe Thr
(in frame with Myc)

aaccccgtgtatgccacactctacatgggggccatggcagtcgccactccctggccagcacggacgagaagcgagaactcctgggccgg 5310
ttggggcacatacggtgtgagatgtacccccggtaccgtcagcggtgagggaccggtcgtgcctgctcttcgctcttgaggacccggcc

805 810 815 820 825 830
Asn Pro Val Tyr Ala Thr Leu Tyr Met Gly Gly His Gly Ser Arg His Ser Leu Ala Ser Thr Asp Glu Lys Arg Glu Leu Leu Gly Arg
LRP1mini

Asn Pro Val Tyr Ala Thr Leu Tyr Met Gly Gly His Gly Ser Arg His Ser Leu Ala Ser Thr Asp Glu Lys Arg Glu Leu Leu Gly Arg
(in frame with Myc)

KflI XbaI

ggccctgaggacgagataggggaccccttggcaTACCCATACGATGTTCCAGATTACGCTTAGTCTAGAGAACCATCAGATGTTTCCAGG 5400
ccgggactcctgctctatcccctggggaaccgtATGGGTATGCTACAAGGTCTAATGCGAATCAGATCTCTTGGTAGTCTACAAAGGTCC

835 840 1 5
Gly Pro Glu Asp Glu Ile Gly Asp Pro Leu Ala Tyr Pro Tyr Asp Val Pro Asp Tyr Ala *
LRP1mini HA

Gly Pro Glu Asp Glu Ile Gly Asp Pro Leu Ala Tyr Pro Tyr Asp Val Pro Asp Tyr Ala *
(in frame with Myc)

GCTACAAGGTCTAATGCGAATCAGATCTTTTT
XbaI rev

GTGCCCCAAGGACCTGAAATGACCCTGTGCCTTATTTGAACTAACCAATCAGTTCGCTTCTCGCTTCTGTTCGCGCGCTTCTGCTCCCCG 5490
CACGGGGTTCCTGGACTTTACTGGGACACGGAATAAACTTGATTGGTTAGTCAAGCGAAGAGCGAAGACAAGCGCGCGAAGACGAGGGGC

AGCTCAATAAAAGAGCCCACAACCCCTCACTCGGGGCGCCAGTCCTCCGATTGACTGAGTCGCCCGGGTACCCGTGTATCCAATAAACCC 5580
TCGAGTTATTTTCTCGGGTGTTGGGGAGTGAGCCCCGCGGTCAGGAGGCTAACTGACTCAGCGGGCCCATGGGCACATAGGTTATTTGGG

TCTTGCAGTTGCATCCGACTTGTGGTCTCGCTGTTCCTTGGGAGGGTCTCCTCTGAGTGATTGACTACCCGTCAGCGGGGGTCTTTCATT 5670
AGAACGTCAACGTAGGCTGAACACCAGAGCGACAAGGAACCCTCCCAGAGGAGACTCACTAACTGATGGGCAGTCGCCCCCAGAAAGTAA

TGGGGGCTCGTCCGGGATCGGGAGACCCCTGCCCAGGGACCACCGACCCACCACCGGGAGGTAAGCTGGCTGCCTCGCGCGTTTCGGTGA 5760
ACCCCCGAGCAGGCCCTAGCCCTCTGGGGACGGGTCCCTGGTGGCTGGGTGGTGGCCCTCCATTCGACCGACGGAGCGCGCAAAGCCACT

TGACGGTGAAAACCTCTGACACATGCAGCTCCCGGAGACGGTCACAGCTTGTCTGTAAGCGGATGCCGGGAGCAGACAAGCCCGTCAGGG 5850
ACTGCCACTTTTGGAGACTGTGTACGTCGAGGGCCTCTGCCAGTGTCGAACAGACATTCGCCTACGGCCCTCGTCTGTTCGGGCAGTCCC

AccI BstZ17I

CGCGTCAGCGGGTGTTGGCGGGTGTCGGGGCGCAGCCATGACCCAGTCACGTAGCGATAGCGGAGTGTATACTGGCTTAACTATGCGGCA 5940
GCGCAGTCGCCCACAACCGCCCACAGCCCCGCGTCGGTACTGGGTCAGTGCATCGCTATCGCCTCACATATGACCGAATTGATACGCCGT
bom

TCAGAGCAGATTGTACTGAGAGTGCACCATATGCGGTGTGAAATACCGCACAGATGCGTAAGGAGAAAATACCGCATCAGGCGCTCTTCC 6030
AGTCTCGTCTAACATGACTCTCACGTGGTATACGCCACACTTTATGGCGTGTCTACGCATTCCTCTTTTATGGCGTAGTCCGCGAGAAGG
bom

GCTTCCTCGCTCACTGACTCGCTGCGCTCGGTCGTTCGGCTGCGGCGAGCGGTATCAGCTCACTCAAAGGCGGTAATACGGTTATCCACA 6120
CGAAGGAGCGAGTGACTGAGCGACGCGAGCCAGCAAGCCGACGCCGCTCGCCATAGTCGAGTGAGTTTCCGCCATTATGCCAATAGGTGT

GAATCAGGGGATAACGCAGGAAAGAACATGTGAGCAAAAGGCCAGCAAAAGGCCAGGAACCGTAAAAAGGCCGCGTTGCTGGCGTTTTTC 6210
CTTAGTCCCCTATTGCGTCCTTTCTTGTACACTCGTTTTCCGGTCGTTTTCCGGTCCTTGGCATTTTTCCGGCGCAACGACCGCAAAAAG
ori

CATAGGCTCCGCCCCCCTGACGAGCATCACAAAAATCGACGCTCAAGTCAGAGGTGGCGAAACCCGACAGGACTATAAAGATACCAGGCG 6300
GTATCCGAGGCGGGGGGACTGCTCGTAGTGTTTTTAGCTGCGAGTTCAGTCTCCACCGCTTTGGGCTGTCCTGATATTTCTATGGTCCGC
ori

TTTCCCCCTGGAAGCTCCCTCGTGCGCTCTCCTGTTCCGACCCTGCCGCTTACCGGATACCTGTCCGCCTTTCTCCCTTCGGGAAGCGTG 6390
AAAGGGGGACCTTCGAGGGAGCACGCGAGAGGACAAGGCTGGGACGGCGAATGGCCTATGGACAGGCGGAAAGAGGGAAGCCCTTCGCAC
ori

GCGCTTTCTCATAGCTCACGCTGTAGGTATCTCAGTTCGGTGTAGGTCGTTCGCTCCAAGCTGGGCTGTGTGCACGAACCCCCCGTTCAG 6480
CGCGAAAGAGTATCGAGTGCGACATCCATAGAGTCAAGCCACATCCAGCAAGCGAGGTTCGACCCGACACACGTGCTTGGGGGGCAAGTC
ori

CCCGACCGCTGCGCCTTATCCGGTAACTATCGTCTTGAGTCCAACCCGGTAAGACACGACTTATCGCCACTGGCAGCAGCCACTGGTAAC 6570
GGGCTGGCGACGCGGAATAGGCCATTGATAGCAGAACTCAGGTTGGGCCATTCTGTGCTGAATAGCGGTGACCGTCGTCGGTGACCATTG
ori

AGGATTAGCAGAGCGAGGTATGTAGGCGGTGCTACAGAGTTCTTGAAGTGGTGGCCTAACTACGGCTACACTAGAAGGACAGTATTTGGT 6660
TCCTAATCGTCTCGCTCCATACATCCGCCACGATGTCTCAAGAACTTCACCACCGGATTGATGCCGATGTGATCTTCCTGTCATAAACCA
ori

ATCTGCGCTCTGCTGAAGCCAGTTACCTTCGGAAAAAGAGTTGGTAGCTCTTGATCCGGCAAACAAACCACCGCTGGTAGCGGTGGTTTT 6750
TAGACGCGAGACGACTTCGGTCAATGGAAGCCTTTTTCTCAACCATCGAGAACTAGGCCGTTTGTTTGGTGGCGACCATCGCCACCAAAA
ori

TTTGTTTGCAAGCAGCAGATTACGCGCAGAAAAAAAGGATCTCAAGAAGATCCTTTGATCTTTTCTACGGGGTCTGACGCTCAGTGGAAC 6840
AAACAAACGTTCGTCGTCTAATGCGCGTCTTTTTTTCCTAGAGTTCTTCTAGGAAACTAGAAAAGATGCCCCAGACTGCGAGTCACCTTG
ori

GAAAACTCACGTTAAGGGATTTTGGTCATGAGATTATCAAAAAGGATCTTCACCTAGATCCTTTTAAATTAAAAATGAAGTTTTAAATCA 6930
CTTTTGAGTGCAATTCCCTAAAACCAGTACTCTAATAGTTTTTCCTAGAAGTGGATCTAGGAAAATTTAATTTTTACTTCAAAATTTAGT

ATCTAAAGTATATATGAGTAAACTTGGTCTGACAGTTACCAATGCTTAATCAGTGAGGCACCTATCTCAGCGATCTGTCTATTTCGTTCA 7020
TAGATTTCATATATACTCATTTGAACCAGACTGTCAATGGTTACGAATTAGTCACTCCGTGGATAGAGTCGCTAGACAGATAAAGCAAGT
285 280 275 270
* Trp His Lys Ile Leu Ser Ala Gly Ile Glu Ala Ile Gln Arg Asn Arg Glu
AmpR

TCCATAGTTGCCTGACTCCCCGTCGTGTAGATAACTACGATACGGGAGGGCTTACCATCTGGCCCCAGTGCTGCAATGATACCGCGAGAC 7110
AGGTATCAACGGACTGAGGGGCAGCACATCTATTGATGCTATGCCCTCCCGAATGGTAGACCGGGGTCACGACGTTACTATGGCGCTCTG
265 260 255 250 245 240
Asp Met Thr Ala Gln Ser Gly Thr Thr Tyr Ile Val Val Ile Arg Ser Pro Lys Gly Asp Pro Gly Leu Ala Ala Ile Ile Gly Arg Ser
AmpR

CCACGCTCACCGGCTCCAGATTTATCAGCAATAAACCAGCCAGCCGGAAGGGCCGAGCGCAGAAGTGGTCCTGCAACTTTATCCGCCTCC 7200
GGTGCGAGTGGCCGAGGTCTAAATAGTCGTTATTTGGTCGGTCGGCCTTCCCGGCTCGCGTCTTCACCAGGACGTTGAAATAGGCGGAGG
235 230 225 220 215 210
Gly Arg Glu Gly Ala Gly Ser Lys Asp Ala Ile Phe Trp Gly Ala Pro Leu Ala Ser Arg Leu Leu Pro Gly Ala Val Lys Asp Ala Glu
AmpR

AseI
ATCCAGTCTATTAATTGTTGCCGGGAAGCTAGAGTAAGTAGTTCGCCAGTTAATAGTTTGCGCAACGTTGTTGCCATTGCTGCAGGCATC 7290
TAGGTCAGATAATTAACAACGGCCCTTCGATCTCATTCATCAAGCGGTCAATTATCAAACGCGTTGCAACAACGGTAACGACGTCCGTAG
205 200 195 190 185 180
Met Trp Asp Ile Leu Gln Gln Arg Ser Ala Leu Thr Leu Leu Glu Gly Thr Leu Leu Lys Arg Leu Thr Thr Ala Met Ala Ala Pro Met
AmpR

GTGGTGTCACGCTCGTCGTTTGGTATGGCTTCATTCAGCTCCGGTTCCCAACGATCAAGGCGAGTTACATGATCCCCCATGTTGTGCAAA 7380
CACCACAGTGCGAGCAGCAAACCATACCGAAGTAAGTCGAGGCCAAGGGTTGCTAGTTCCGCTCAATGTACTAGGGGGTACAACACGTTT
175 170 165 160 155 150
Thr Thr Asp Arg Glu Asp Asn Pro Ile Ala Glu Asn Leu Glu Pro Glu Trp Arg Asp Leu Arg Thr Val His Asp Gly Met Asn His Leu
AmpR

AAAGCGGTTAGCTCCTTCGGTCCTCCGATCGTTGTCAGAAGTAAGTTGGCCGCAGTGTTATCACTCATGGTTATGGCAGCACTGCATAAT 7470
TTTCGCCAATCGAGGAAGCCAGGAGGCTAGCAACAGTCTTCATTCAACCGGCGTCACAATAGTGAGTACCAATACCGTCGTGACGTATTA
145 140 135 130 125 120
Phe Ala Thr Leu Glu Lys Pro Gly Gly Ile Thr Thr Leu Leu Leu Asn Ala Ala Thr Asn Asp Ser Met Thr Ile Ala Ala Ser Cys Leu
AmpR

ScaI
TCTCTTACTGTCATGCCATCCGTAAGATGCTTTTCTGTGACTGGTGAGTACTCAACCAAGTCATTCTGAGAATAGTGTATGCGGCGACCG 7560
AGAGAATGACAGTACGGTAGGCATTCTACGAAAAGACACTGACCACTCATGAGTTGGTTCAGTAAGACTCTTATCACATACGCCGCTGGC
115 110 105 100 95 90
Glu Arg Val Thr Met Gly Asp Thr Leu His Lys Glu Thr Val Pro Ser Tyr Glu Val Leu Asp Asn Gln Ser Tyr His Ile Arg Arg Gly
AmpR

XmnI
AGTTGCTCTTGCCCGGCGTCAACACGGGATAATACCGCGCCACATAGCAGAACTTTAAAAGTGCTCATCATTGGAAAACGTTCTTCGGGG 7650
TCAACGAGAACGGGCCGCAGTTGTGCCCTATTATGGCGCGGTGTATCGTCTTGAAATTTTCACGAGTAGTAACCTTTTGCAAGAAGCCCC
85 80 75 70 65 60
Leu Gln Glu Gln Gly Ala Asp Val Arg Ser Leu Val Ala Gly Cys Leu Leu Val Lys Phe Thr Ser Met Met Pro Phe Arg Glu Glu Pro
AmpR

CGAAAACTCTCAAGGATCTTACCGCTGTTGAGATCCAGTTCGATGTAACCCACTCGTGCACCCAACTGATCTTCAGCATCTTTTACTTTC 7740
GCTTTTGAGAGTTCCTAGAATGGCGACAACTCTAGGTCAAGCTACATTGGGTGAGCACGTGGGTTGACTAGAAGTCGTAGAAAATGAAAG
55 50 45 40 35 30
Arg Phe Ser Glu Leu Ile Lys Gly Ser Asn Leu Asp Leu Glu Ile Tyr Gly Val Arg Ala Gly Leu Gln Asp Glu Ala Asp Lys Val Lys
AmpR

ACCAGCGTTTCTGGGTGAGCAAAAACAGGAAGGCAAAATGCCGCAAAAAAGGGAATAAGGGCGACACGGAAATGTTGAATACTCATACTC 7830
TGGTCGCAAAGACCCACTCGTTTTTGTCCTTCCGTTTTACGGCGTTTTTTCCCTTATTCCCGCTGTGCCTTTACAACTTATGAGTATGAG
25 20 15 10 5 1
Val Leu Thr Glu Pro His Ala Phe Val Pro Leu Cys Phe Ala Ala Phe Phe Pro Ile Leu Ala Val Arg Phe His Gln Ile Ser Met
signal sequence
AmpR
AmpR promoter

SspI
TTCCTTTTTCAATATTATTGAAGCATTTATCAGGGTTATTGTCTCATGAGCGGATACATATTTGAATGTATTTAGAAAAATAAACAAATA 7920
AAGGAAAAAGTTATAATAACTTCGTAAATAGTCCCAATAACAGAGTACTCGCCTATGTATAAACTTACATAAATCTTTTTATTTGTTTAT
AmpR promoter

```
GGGGTTCCGCGCACATTTCCCCGAAAAGTGCCACCTGACGTCTAAGAAACCATTATTATCATGACATTAACCTATAAAAATAGGCGTATC   8010
CCCCAAGGCGCGTGTAAAGGGGCTTTTCACGGTGGACTGCAGATTCTTTGGTAATAATAGTACTGTAATTGGATATTTTTATCCGCATAG
< AmpR promoter

ACGAGGCCCTTTCGTCTTCAAGAATTCATACCAGATCACCGAAAACTGTCCTCCAAATGTGTCCCCCTCACACTCCCAAATTCGCGGGCT   8100
TGCTCCGGGAAAGCAGAAGTTCTTAAGTATGGTCTAGTGGCTTTTGACAGGAGGTTTACACAGGGGGAGTGTGAGGGTTTAAGCGCCCGA

                                                       SacII
TCTGCCTCTTAGACCACTCTACCCTATTCCCCACACTCACCGGAGCCAAAGCCGCGGCCCTTCCGTTTCTTTGCT   3'   ••• 8175
AGACGGAGAATCTGGTGAGATGGGATAAGGGGTGTGAGTGGCCTCGGTTTCGGCGCCGGGAAGGCAAAGAAACGA   5'
```
